# Supplementary figures and images for: UBTD1 regulates ceramide balance and endolysosomal positioning to coordinate EGFR signaling
Source: eLife. 2021 Apr 22;10:e68348. doi: 10.7554/eLife.68348 (PMC8118655; doi:10.7554/eLife.68348)

**B**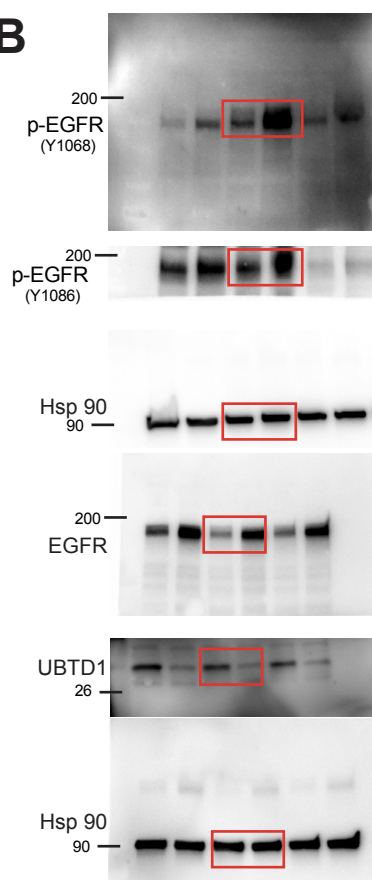**Figure 1-source data 1**

Supplement: Figure 1—source data 1. [file elife-68348-fig1-data1.pdf]

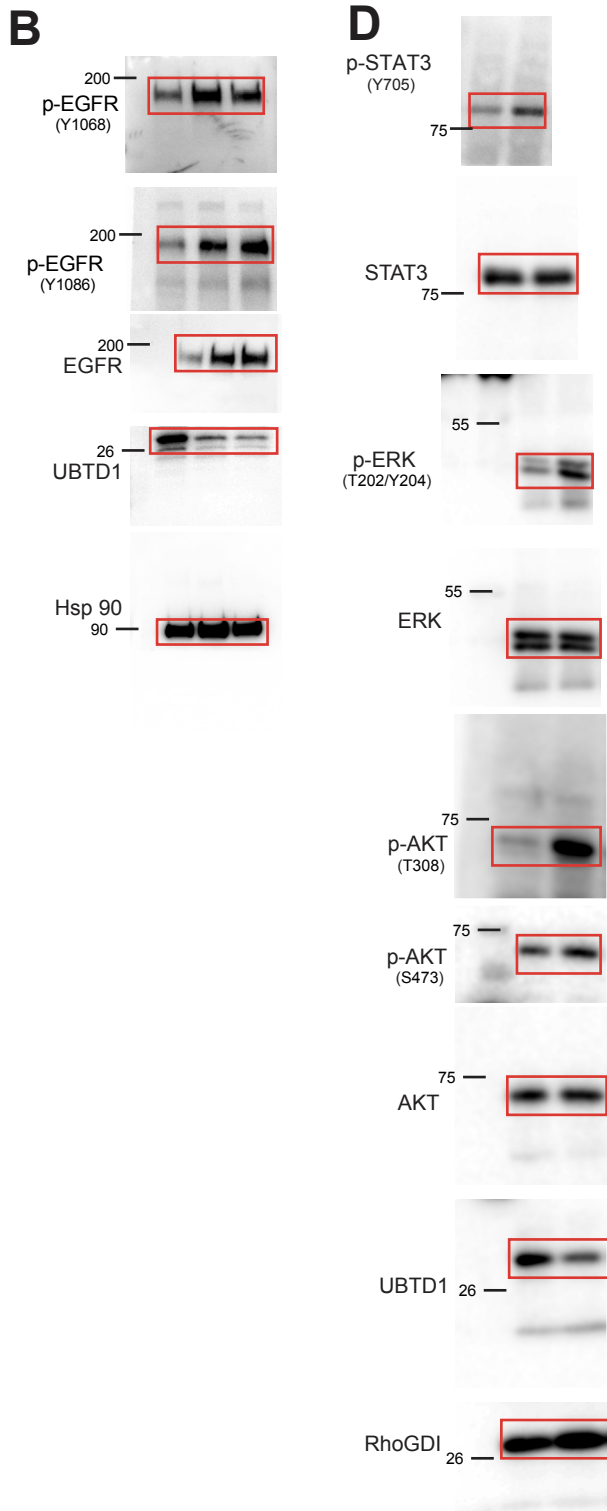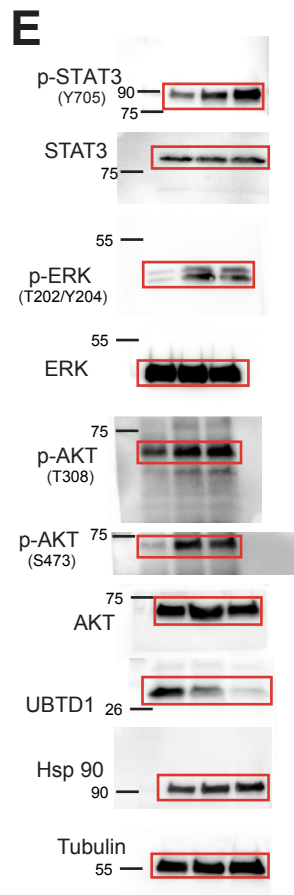

Figure 1-source data 2

Supplement: Figure 1—figure supplement 1—source data 1. [file elife-68348-fig1-figsupp1-data1.pdf]

**E**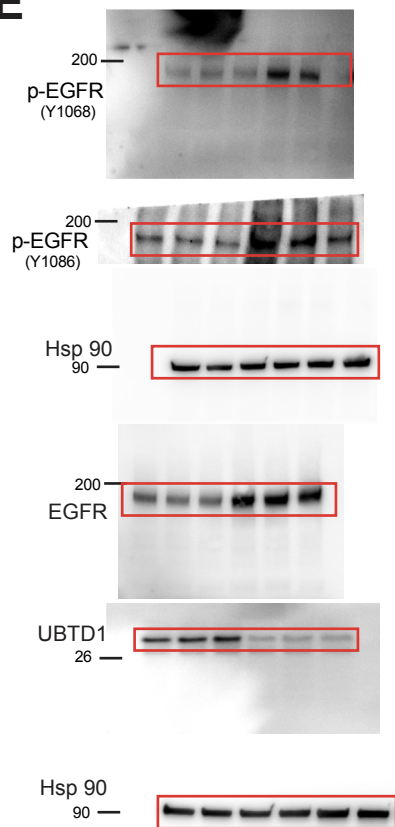**Figure 2-source data 1**

Supplement: Figure 2—source data 1. [file elife-68348-fig2-data1.pdf]

**E**

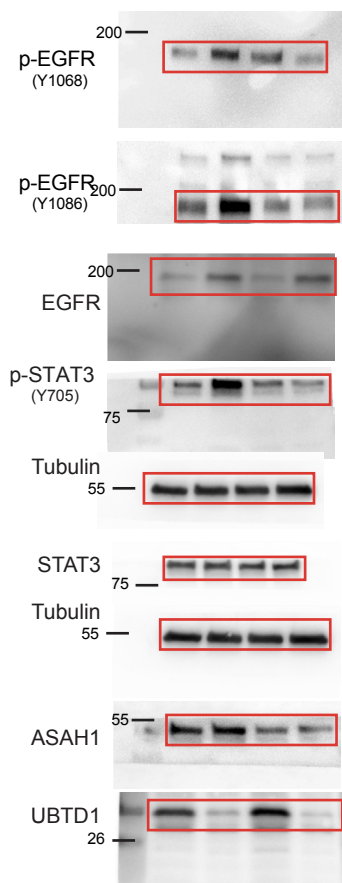

**Figure 2-source data 2**

Supplement: Figure 2—figure supplement 1—source data 1. [file elife-68348-fig2-figsupp1-data1.pdf]

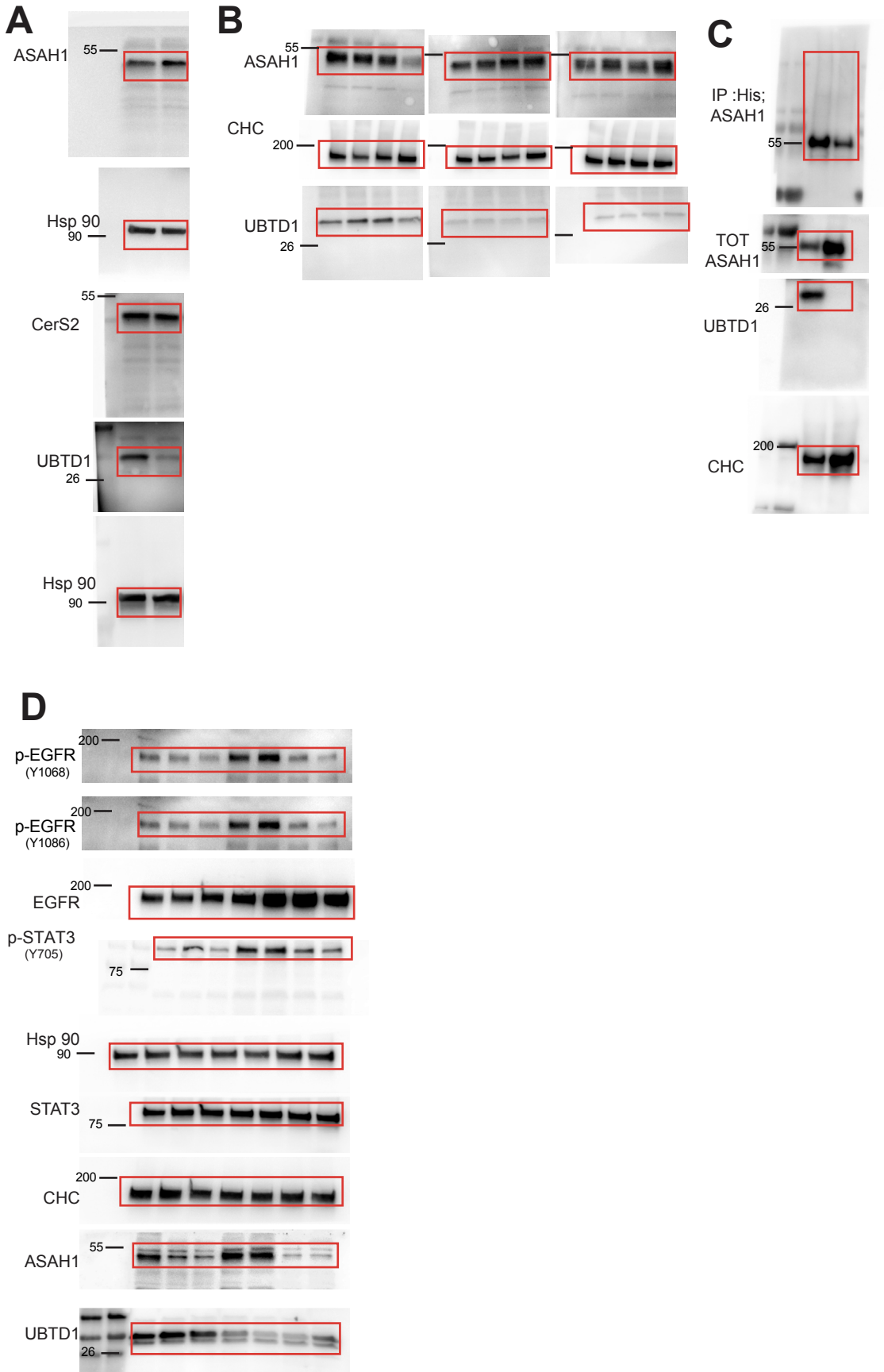

Figure 3-source data 1

Supplement: Figure 3—source data 1. [file elife-68348-fig3-data1.pdf]

**A**

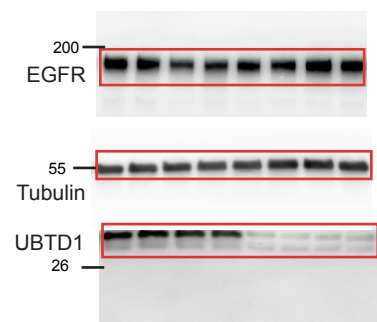

Supplement: Figure 4—source data 1. [file elife-68348-fig4-data1.pdf]

**A**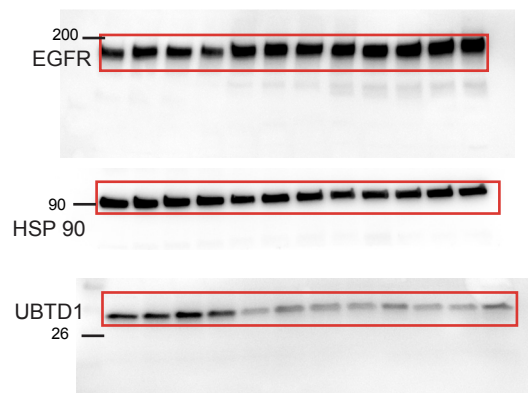**B**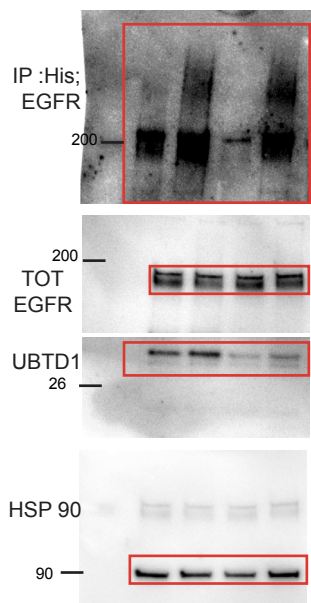**Figure 4-source data 2**

Supplement: Figure 4—figure supplement 1—source data 1. [file elife-68348-fig4-figsupp1-data1.pdf]

**C**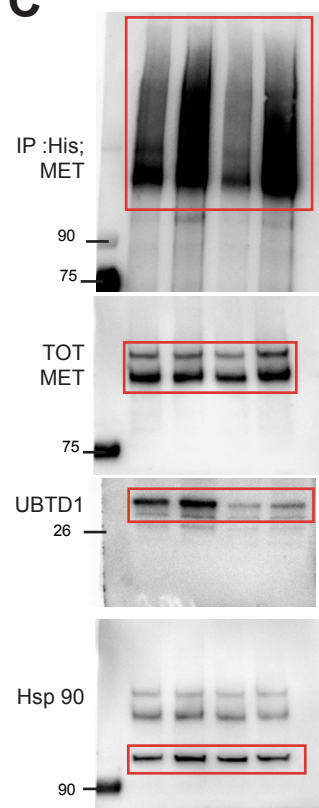**D**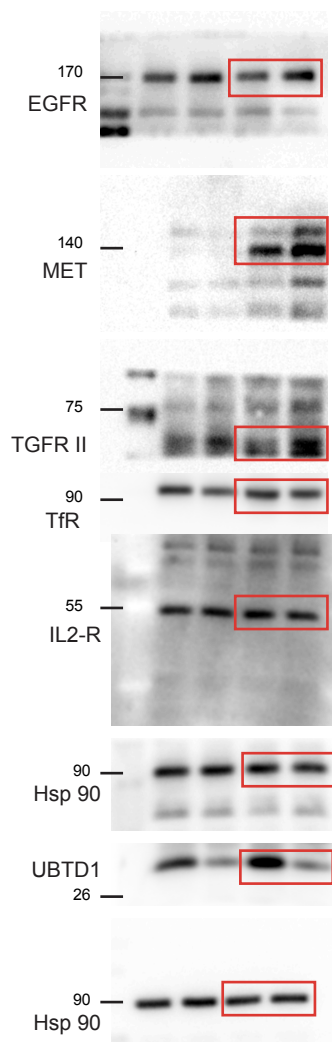**Figure 4-source data 3**

Supplement: Figure 4—figure supplement 1—source data 2. [file elife-68348-fig4-figsupp1-data2.pdf]

**D**

IP :UBTD1

75  
P62

UBTD1 26

Total

Hsp90

90

75  
P62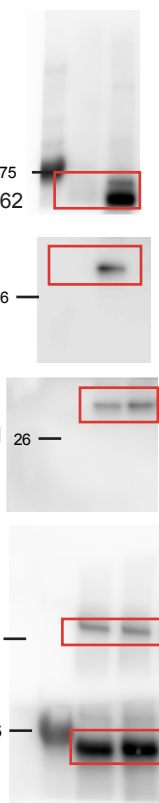

Supplement: Figure 5—source data 1. [file elife-68348-fig5-data1.pdf]

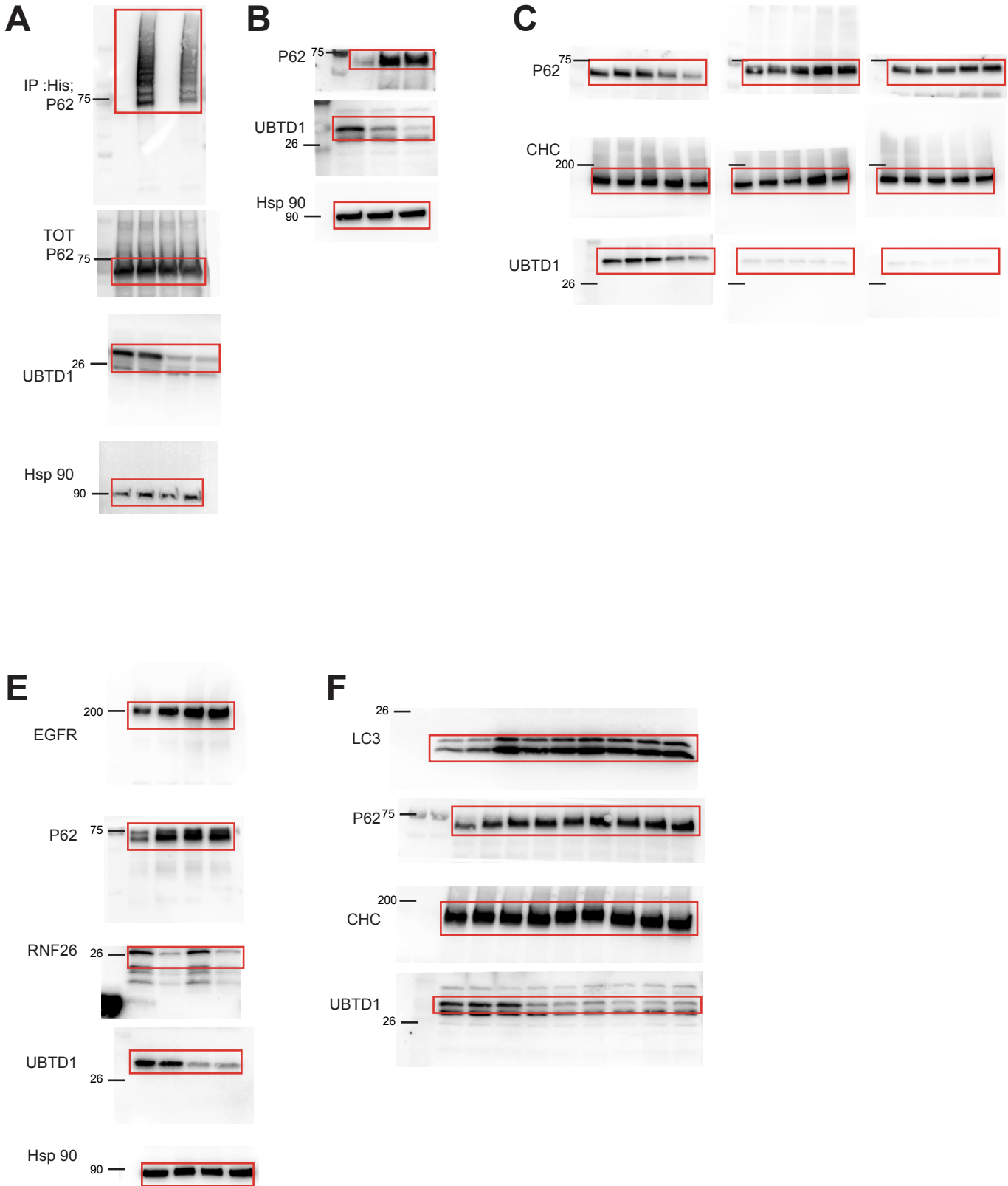

Figure 6-source data 1

Supplement: Figure 6—source data 1. [file elife-68348-fig6-data1.pdf]

**A**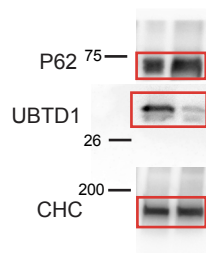**B**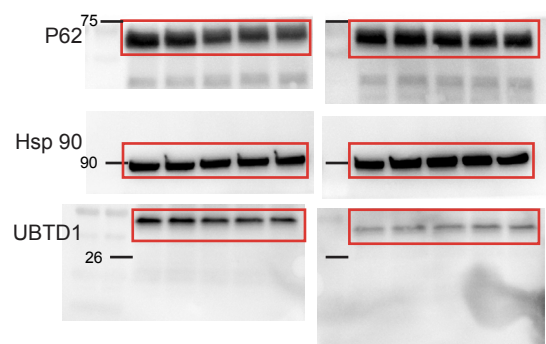**D**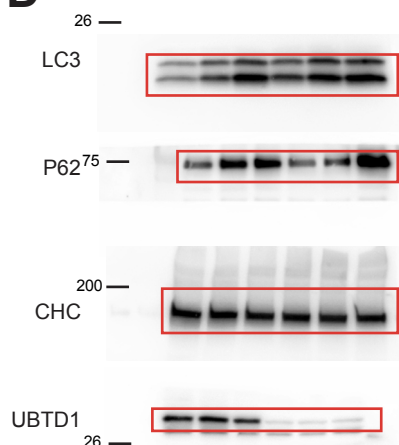

Supplement: Figure 6—figure supplement 1—source data 1. [file elife-68348-fig6-figsupp1-data1.pdf]
