# Supplementary material for: UBTD1 regulates ceramide balance and endolysosomal positioning to coordinate EGFR signaling
Source: eLife. 2021 Apr 22;10:e68348. doi: 10.7554/eLife.68348 (PMC8118655; doi:10.7554/eLife.68348)
Supplement: Supplementary file 1. [file elife-68348-supp1.docx]

**Figure 2- Supplementary file 1: Proteins identified by LC-MS/MS following UBTD1 immunoprecipitation in DU145 cell line.**

| **Accession** | **Description** | **Coverage**  **[%]** | **# Peptides** | **#**  **PSMs** | **# Unique**  **Peptides** | **Score**  **Sequest**  **HT** | **#**  **Peptides** |
| --- | --- | --- | --- | --- | --- | --- | --- |
|  |  |  |  |  |  |  |  |
| Q562R1 | Beta-actin-like protein 2 OS=Homo sapiens OX=9606 GN=ACTBL2 PE=1 SV=2 | 18 | 9 | 181 | 1 | 219,08 | 9 |
| Q9ULV0 | Unconventional myosin-Vb OS=Homo sapiens OX=9606 GN=MYO5B PE=1 SV=3 | 3 | 7 | 17 | 2 | 43,42 | 7 |
| P15170 | Eukaryotic peptide chain release factor GTP-binding subunit ERF3A OS=Homo sapiens OX=9606 GN=GSPT1 PE=1 SV=1 | 7 | 3 | 3 | 3 | 7,51 | 3 |
| P62277 | 40S ribosomal protein S13 OS=Homo sapiens OX=9606 GN=RPS13 PE=1 SV=2 | 15 | 2 | 2 | 2 | 4,99 | 2 |
| P10768 | S-formylglutathione hydrolase OS=Homo sapiens OX=9606 GN=ESD PE=1 SV=2 | 7 | 2 | 2 | 2 | 2,84 | 2 |
| Q86UP2 | Kinectin OS=Homo sapiens OX=9606 GN=KTN1 PE=1 SV=1 | 1 | 1 | 1 | 1 | 2,56 | 1 |
| Q9NYU2 | UDP-glucose:glycoprotein glucosyltransferase 1 OS=Homo sapiens OX=9606 GN=UGGT1 PE=1 SV=3 | 1 | 1 | 2 | 1 | 4,16 | 1 |
| P54727 | UV excision repair protein RAD23 homolog B OS=Homo sapiens OX=9606 GN=RAD23B PE=1 SV=1 | 9 | 2 | 3 | 2 | 6,52 | 2 |
| P09382 | Galectin-1 OS=Homo sapiens OX=9606 GN=LGALS1 PE=1 SV=2 | 15 | 2 | 3 | 2 | 6,64 | 2 |
| P51659 | Peroxisomal multifunctional enzyme type 2 OS=Homo sapiens OX=9606 GN=HSD17B4 PE=1 SV=3 | 2 | 2 | 4 | 2 | 8,15 | 2 |
| Q99439 | Calponin-2 OS=Homo sapiens OX=9606 GN=CNN2 PE=1 SV=4 | 7 | 2 | 4 | 2 | 9,56 | 2 |
| O60488 | Long-chain-fatty-acid--CoA ligase 4 OS=Homo sapiens OX=9606 GN=ACSL4 PE=1 SV=2 | 2 | 1 | 1 | 1 | 2,92 | 1 |
| O75534 | Cold shock domain-containing protein E1 OS=Homo sapiens OX=9606 GN=CSDE1 PE=1 SV=2 | 4 | 3 | 4 | 3 | 3,26 | 3 |
| P54886 | Delta-1-pyrroline-5-carboxylate synthase OS=Homo sapiens OX=9606 GN=ALDH18A1 PE=1 SV=2 | 2 | 2 | 3 | 2 | 3,65 | 2 |
| Q9BVP2 | Guanine nucleotide-binding protein-like 3 OS=Homo sapiens OX=9606 GN=GNL3 PE=1 SV=2 | 3 | 2 | 3 | 2 | 5,28 | 2 |

| Q13596 | Sorting nexin-1 OS=Homo sapiens OX=9606 GN=SNX1 PE=1 SV=3 | 4 | 2 | 2 | 2 | 2,44 | 2 |
| --- | --- | --- | --- | --- | --- | --- | --- |
| Q15637 | Splicing factor 1 OS=Homo sapiens OX=9606 GN=SF1 PE=1 SV=4 | 2 | 1 | 2 | 1 | 4,2 | 1 |
| P60953 | Cell division control protein 42 homolog OS=Homo sapiens OX=9606 GN=CDC42 PE=1 SV=2 | 5 | 1 | 3 | 1 | 1,81 | 1 |
| P24390 | ER lumen protein-retaining receptor 1 OS=Homo sapiens OX=9606 GN=KDELR1 PE=1 SV=1 | 5 | 1 | 2 | 1 | 4,73 | 1 |
| Q96CS3 | FAS-associated factor 2 OS=Homo sapiens OX=9606 GN=FAF2 PE=1 SV=2 | 2 | 1 | 1 | 1 | 1,86 | 1 |
| Q8TF05 | Serine/threonine-protein phosphatase 4 regulatory subunit 1 OS=Homo sapiens OX=9606 GN=PPP4R1 PE=1 SV=1 | 2 | 2 | 2 | 2 | 1,61 | 2 |
| Q9BWD1 | Acetyl-CoA acetyltransferase, cytosolic OS=Homo sapiens OX=9606 GN=ACAT2 PE=1 SV=2 | 5 | 2 | 2 | 2 | 2,01 | 2 |
| P39748 | Flap endonuclease 1 OS=Homo sapiens OX=9606 GN=FEN1 PE=1 SV=1 | 3 | 1 | 2 | 1 | 3,96 | 1 |
| Q69YQ0 | Cytospin-A OS=Homo sapiens OX=9606 GN=SPECC1L PE=1 SV=2 | 1 | 1 | 1 | 1 | 2,23 | 1 |
| Q9Y4B5 | Microtubule cross-linking factor 1 OS=Homo sapiens OX=9606 GN=MTCL1 PE=1 SV=5 | 31 | 53 | 121 | 53 | 323,17 | 53 |
| P46013 | Proliferation marker protein Ki-67 OS=Homo sapiens OX=9606 GN=MKI67 PE=1 SV=2 | 22 | 49 | 78 | 49 | 185,12 | 49 |
| Q13586 | Stromal interaction molecule 1 OS=Homo sapiens OX=9606 GN=STIM1 PE=1 SV=3 | 36 | 23 | 48 | 23 | 156,95 | 23 |
| Q9UQ35 | Serine/arginine repetitive matrix protein 2 OS=Homo sapiens OX=9606 GN=SRRM2 PE=1 SV=2 | 13 | 25 | 47 | 25 | 132,22 | 25 |
| Q14C86 | GTPase-activating protein and VPS9 domain-containing protein 1 OS=Homo sapiens OX=9606 GN=GAPVD1 PE=1 SV=2 | 24 | 32 | 54 | 32 | 134,61 | 32 |
| P27708 | CAD protein OS=Homo sapiens OX=9606 GN=CAD PE=1 SV=3 | 16 | 32 | 60 | 32 | 152,53 | 32 |
| O76031 | ATP-dependent Clp protease ATP-binding subunit clpX-like, mitochondrial OS=Homo sapiens OX=9606 GN=CLPX PE=1 SV=2 | 32 | 22 | 50 | 22 | 133,94 | 22 |
| Q9Y613 | FH1/FH2 domain-containing protein 1 OS=Homo sapiens OX=9606 GN=FHOD1 PE=1 SV=3 | 24 | 25 | 44 | 25 | 110,69 | 25 |
| Q13509 | Tubulin beta-3 chain OS=Homo sapiens OX=9606 GN=TUBB3 PE=1 SV=2 | 28 | 12 | 55 | 1 | 143,44 | 12 |

| Q16740 | ATP-dependent Clp protease proteolytic subunit, mitochondrial OS=Homo sapiens OX=9606 GN=CLPP PE=1 SV=1 | 43 | 11 | 32 | 11 | 97,62 | 11 |
| --- | --- | --- | --- | --- | --- | --- | --- |
| Q14315 | Filamin-C OS=Homo sapiens OX=9606 GN=FLNC PE=1 SV=3 | 8 | 19 | 30 | 11 | 71,45 | 19 |
| Q14152 | Eukaryotic translation initiation factor 3 subunit A OS=Homo sapiens OX=9606 GN=EIF3A PE=1 SV=1 | 16 | 22 | 31 | 22 | 70,14 | 22 |
| P13667 | Protein disulfide-isomerase A4 OS=Homo sapiens OX=9606 GN=PDIA4 PE=1 SV=2 | 29 | 14 | 25 | 14 | 58,51 | 14 |
| Q86VP6 | Cullin-associated NEDD8-dissociated protein 1 OS=Homo sapiens OX=9606 GN=CAND1 PE=1 SV=2 | 13 | 15 | 24 | 15 | 60,19 | 15 |
| Q92841 | Probable ATP-dependent RNA helicase DDX17 OS=Homo sapiens OX=9606 GN=DDX17 PE=1 SV=2 | 19 | 13 | 22 | 8 | 50,88 | 13 |
| Q9BUF5 | Tubulin beta-6 chain OS=Homo sapiens OX=9606 GN=TUBB6 PE=1 SV=1 | 26 | 10 | 36 | 3 | 96,65 | 10 |
| P05023 | Sodium/potassium-transporting ATPase subunit alpha-1 OS=Homo sapiens OX=9606 GN=ATP1A1 PE=1 SV=1 | 14 | 12 | 17 | 12 | 47,08 | 12 |
| Q9Y4L1 | Hypoxia up-regulated protein 1 OS=Homo sapiens OX=9606 GN=HYOU1 PE=1 SV=1 | 16 | 15 | 24 | 15 | 49,67 | 15 |
| Q14596 | Next to BRCA1 gene 1 protein OS=Homo sapiens OX=9606 GN=NBR1 PE=1 SV=3 | 13 | 10 | 17 | 10 | 48,42 | 10 |
| Q92616 | eIF-2-alpha kinase activator GCN1 OS=Homo sapiens OX=9606 GN=GCN1 PE=1 SV=6 | 8 | 21 | 28 | 21 | 50,06 | 21 |
| Q969G5 | Caveolae-associated protein 3 OS=Homo sapiens OX=9606 GN=CAVIN3 PE=1 SV=3 | 31 | 8 | 24 | 8 | 65,94 | 8 |
| P78371 | T-complex protein 1 subunit beta OS=Homo sapiens OX=9606 GN=CCT2 PE=1 SV=4 | 25 | 12 | 19 | 12 | 49,95 | 12 |
| Q04637 | Eukaryotic translation initiation factor 4 gamma 1 OS=Homo sapiens OX=9606 GN=EIF4G1 PE=1 SV=4 | 11 | 12 | 17 | 12 | 47,9 | 12 |
| O60664 | Perilipin-3 OS=Homo sapiens OX=9606 GN=PLIN3 PE=1 SV=3 | 20 | 6 | 7 | 6 | 24,13 | 6 |
| O95810 | Caveolae-associated protein 2 OS=Homo sapiens OX=9606 GN=CAVIN2 PE=1 SV=3 | 21 | 8 | 15 | 8 | 39,6 | 8 |
| P08133 | Annexin A6 OS=Homo sapiens OX=9606 GN=ANXA6 PE=1 SV=3 | 22 | 14 | 21 | 14 | 38,41 | 14 |
| P09960 | Leukotriene A-4 hydrolase OS=Homo sapiens OX=9606 GN=LTA4H PE=1 SV=2 | 19 | 10 | 17 | 10 | 36,62 | 10 |

| P22102 | Trifunctional purine biosynthetic protein adenosine-3 OS=Homo sapiens OX=9606 GN=GART PE=1 SV=1 | 13 | 11 | 15 | 11 | 31,64 | 11 |
| --- | --- | --- | --- | --- | --- | --- | --- |
| Q14764 | Major vault protein OS=Homo sapiens OX=9606 GN=MVP PE=1 SV=4 | 13 | 10 | 14 | 10 | 33,49 | 10 |
| Q04206 | Transcription factor p65 OS=Homo sapiens OX=9606 GN=RELA PE=1 SV=2 | 17 | 8 | 13 | 8 | 33,97 | 8 |
| Q9UPN4 | Centrosomal protein of 131 kDa OS=Homo sapiens OX=9606 GN=CEP131 PE=1 SV=3 | 10 | 8 | 12 | 8 | 28 | 8 |
| Q15393 | Splicing factor 3B subunit 3 OS=Homo sapiens OX=9606 GN=SF3B3 PE=1 SV=4 | 9 | 10 | 13 | 10 | 29,88 | 10 |
| Q15029 | 116 kDa U5 small nuclear ribonucleoprotein component OS=Homo sapiens OX=9606 GN=EFTUD2 PE=1 SV=1 | 11 | 9 | 17 | 8 | 38,39 | 9 |
| Q13283 | Ras GTPase-activating protein-binding protein 1 OS=Homo sapiens OX=9606 GN=G3BP1 PE=1 SV=1 | 18 | 6 | 12 | 6 | 25,14 | 6 |
| P41252 | Isoleucine--tRNA ligase, cytoplasmic OS=Homo sapiens OX=9606 GN=IARS PE=1 SV=2 | 8 | 9 | 14 | 9 | 34,32 | 9 |
| P53621 | Coatomer subunit alpha OS=Homo sapiens OX=9606 GN=COPA PE=1 SV=2 | 7 | 8 | 11 | 8 | 30,6 | 8 |
| P56192 | Methionine--tRNA ligase, cytoplasmic OS=Homo sapiens OX=9606 GN=MARS PE=1 SV=2 | 10 | 7 | 10 | 7 | 22,8 | 7 |
| Q08379 | Golgin subfamily A member 2 OS=Homo sapiens OX=9606 GN=GOLGA2 PE=1 SV=3 | 8 | 6 | 11 | 6 | 28,13 | 6 |
| Q12931 | Heat shock protein 75 kDa, mitochondrial OS=Homo sapiens OX=9606 GN=TRAP1 PE=1 SV=3 | 11 | 7 | 14 | 6 | 41,13 | 7 |
| Q86YT6 | E3 ubiquitin-protein ligase MIB1 OS=Homo sapiens OX=9606 GN=MIB1 PE=1 SV=1 | 10 | 7 | 12 | 7 | 33,45 | 7 |
| P11216 | Glycogen phosphorylase, brain form OS=Homo sapiens OX=9606 GN=PYGB PE=1 SV=5 | 11 | 8 | 10 | 4 | 23,39 | 8 |
| P49736 | DNA replication licensing factor MCM2 OS=Homo sapiens OX=9606 GN=MCM2 PE=1 SV=4 | 9 | 7 | 11 | 7 | 26,08 | 7 |
| P25205 | DNA replication licensing factor MCM3 OS=Homo sapiens OX=9606 GN=MCM3 PE=1 SV=3 | 10 | 9 | 12 | 9 | 20,09 | 9 |
| O60506 | Heterogeneous nuclear ribonucleoprotein Q OS=Homo sapiens OX=9606 GN=SYNCRIP PE=1 SV=2 | 13 | 7 | 10 | 3 | 22,18 | 7 |

| Q6P2Q9 | Pre-mRNA-processing-splicing factor 8 OS=Homo sapiens OX=9606 GN=PRPF8 PE=1 SV=2 | 4 | 12 | 16 | 12 | 23,76 | 12 |
| --- | --- | --- | --- | --- | --- | --- | --- |
| Q03135 | Caveolin-1 OS=Homo sapiens OX=9606 GN=CAV1 PE=1 SV=4 | 29 | 6 | 21 | 6 | 49,23 | 6 |
| O15067 | Phosphoribosylformylglycinamidine synthase OS=Homo sapiens OX=9606 GN=PFAS PE=1 SV=4 | 7 | 8 | 9 | 8 | 19,64 | 8 |
| O75643 | U5 small nuclear ribonucleoprotein 200 kDa helicase OS=Homo sapiens OX=9606 GN=SNRNP200 PE=1 SV=2 | 4 | 9 | 13 | 9 | 24 | 9 |
| Q16222 | UDP-N-acetylhexosamine pyrophosphorylase OS=Homo sapiens OX=9606 GN=UAP1 PE=1 SV=3 | 14 | 7 | 12 | 7 | 27,55 | 7 |
| P55265 | Double-stranded RNA-specific adenosine deaminase OS=Homo sapiens OX=9606 GN=ADAR PE=1 SV=4 | 7 | 8 | 11 | 8 | 13,69 | 8 |
| P12429 | Annexin A3 OS=Homo sapiens OX=9606 GN=ANXA3 PE=1 SV=3 | 20 | 7 | 13 | 7 | 27,77 | 7 |
| Q14CN4 | Keratin, type II cytoskeletal 72 OS=Homo sapiens OX=9606 GN=KRT72 PE=1 SV=2 | 10 | 6 | 28 | 2 | 55,95 | 6 |
| Q92973 | Transportin-1 OS=Homo sapiens OX=9606 GN=TNPO1 PE=1 SV=2 | 9 | 7 | 12 | 7 | 24,91 | 7 |
| Q16881 | Thioredoxin reductase 1, cytoplasmic OS=Homo sapiens OX=9606 GN=TXNRD1 PE=1 SV=3 | 12 | 7 | 12 | 7 | 28,61 | 7 |
| P47897 | Glutamine--tRNA ligase OS=Homo sapiens OX=9606 GN=QARS PE=1 SV=1 | 10 | 7 | 10 | 7 | 24,84 | 7 |
| P63010 | AP-2 complex subunit beta OS=Homo sapiens OX=9606 GN=AP2B1 PE=1 SV=1 | 9 | 9 | 13 | 9 | 25,18 | 9 |
| P40939 | Trifunctional enzyme subunit alpha, mitochondrial OS=Homo sapiens OX=9606 GN=HADHA PE=1 SV=2 | 11 | 8 | 11 | 8 | 23,36 | 8 |
| Q8WXW3 | Progesterone-induced-blocking factor 1 OS=Homo sapiens OX=9606 GN=PIBF1 PE=1 SV=2 | 11 | 8 | 9 | 8 | 17,49 | 8 |
| Q06210 | Glutamine--fructose-6-phosphate aminotransferase [isomerizing] 1 OS=Homo sapiens OX=9606 GN=GFPT1 PE=1 SV=3 | 10 | 7 | 10 | 7 | 18,67 | 7 |
| Q9UDY2 | Tight junction protein ZO-2 OS=Homo sapiens OX=9606 GN=TJP2 PE=1 SV=2 | 8 | 8 | 10 | 8 | 18,9 | 8 |
| O95373 | Importin-7 OS=Homo sapiens OX=9606 GN=IPO7 PE=1 SV=1 | 6 | 5 | 8 | 5 | 22,19 | 5 |
| Q15046 | Lysine--tRNA ligase OS=Homo sapiens OX=9606 GN=KARS PE=1 SV=3 | 12 | 7 | 11 | 7 | 20,24 | 7 |
| P14649 | Myosin light chain 6B OS=Homo sapiens OX=9606 GN=MYL6B PE=1 SV=1 | 26 | 5 | 17 | 3 | 36,63 | 5 |

| Q14240 | Eukaryotic initiation factor 4A-II OS=Homo sapiens OX=9606 GN=EIF4A2 PE=1 SV=2 | 18 | 7 | 10 | 1 | 23,89 | 7 |
| --- | --- | --- | --- | --- | --- | --- | --- |
| P27797 | Calreticulin OS=Homo sapiens OX=9606 GN=CALR PE=1 SV=1 | 15 | 4 | 5 | 4 | 15,99 | 4 |
| Q13310 | Polyadenylate-binding protein 4 OS=Homo sapiens OX=9606 GN=PABPC4 PE=1 SV=1 | 9 | 6 | 12 | 2 | 27,47 | 6 |
| O43143 | Pre-mRNA-splicing factor ATP-dependent RNA helicase DHX15 OS=Homo sapiens OX=9606 GN=DHX15 PE=1 SV=2 | 9 | 7 | 10 | 7 | 14,09 | 7 |
| Q6PIU2 | Neutral cholesterol ester hydrolase 1 OS=Homo sapiens OX=9606 GN=NCEH1 PE=1 SV=3 | 17 | 6 | 9 | 6 | 17,47 | 6 |
| Q9Y5B9 | FACT complex subunit SPT16 OS=Homo sapiens OX=9606 GN=SUPT16H PE=1 SV=1 | 8 | 6 | 9 | 6 | 20,13 | 6 |
| O14980 | Exportin-1 OS=Homo sapiens OX=9606 GN=XPO1 PE=1 SV=1 | 8 | 9 | 13 | 9 | 21,94 | 9 |
| P31939 | Bifunctional purine biosynthesis protein PURH OS=Homo sapiens OX=9606 GN=ATIC PE=1 SV=3 | 11 | 5 | 6 | 5 | 18,23 | 5 |
| Q96QK1 | Vacuolar protein sorting-associated protein 35 OS=Homo sapiens OX=9606 GN=VPS35 PE=1 SV=2 | 8 | 6 | 9 | 6 | 24,58 | 6 |
| P14866 | Heterogeneous nuclear ribonucleoprotein L OS=Homo sapiens OX=9606 GN=HNRNPL PE=1 SV=2 | 12 | 5 | 10 | 5 | 21,84 | 5 |
| Q15181 | Inorganic pyrophosphatase OS=Homo sapiens OX=9606 GN=PPA1 PE=1 SV=2 | 22 | 5 | 9 | 5 | 18,68 | 5 |
| P34897 | Serine hydroxymethyltransferase, mitochondrial OS=Homo sapiens OX=9606 GN=SHMT2 PE=1 SV=3 | 14 | 7 | 7 | 7 | 14,85 | 7 |
| Q8NBS9 | Thioredoxin domain-containing protein 5 OS=Homo sapiens OX=9606 GN=TXNDC5 PE=1 SV=2 | 13 | 5 | 7 | 5 | 17,17 | 5 |
| P55084 | Trifunctional enzyme subunit beta, mitochondrial OS=Homo sapiens OX=9606 GN=HADHB PE=1 SV=3 | 11 | 5 | 8 | 5 | 21,18 | 5 |
| Q14694 | Ubiquitin carboxyl-terminal hydrolase 10 OS=Homo sapiens OX=9606 GN=USP10 PE=1 SV=2 | 8 | 5 | 8 | 5 | 18,98 | 5 |
| O00469 | Procollagen-lysine,2-oxoglutarate 5-dioxygenase 2 OS=Homo sapiens OX=9606 GN=PLOD2 PE=1 SV=2 | 9 | 6 | 9 | 6 | 19,51 | 6 |
| Q9HAC8 | Ubiquitin domain-containing protein 1 OS=Homo sapiens OX=9606 GN=UBTD1 PE=1 SV=1 | 29 | 6 | 11 | 6 | 20,34 | 6 |

| Q9BSJ8 | Extended synaptotagmin-1 OS=Homo sapiens OX=9606 GN=ESYT1 PE=1 SV=1 | 5 | 5 | 9 | 5 | 23,58 | 5 |
| --- | --- | --- | --- | --- | --- | --- | --- |
| P38919 | Eukaryotic initiation factor 4A-III OS=Homo sapiens OX=9606 GN=EIF4A3 PE=1 SV=4 | 18 | 8 | 14 | 7 | 31,8 | 8 |
| Q8IU81 | Interferon regulatory factor 2-binding protein 1 OS=Homo sapiens OX=9606 GN=IRF2BP1 PE=1 SV=1 | 12 | 5 | 8 | 5 | 18,48 | 5 |
| P42224 | Signal transducer and activator of transcription 1-alpha/beta OS=Homo sapiens OX=9606 GN=STAT1 PE=1 SV=2 | 10 | 7 | 9 | 7 | 19,77 | 7 |
| P30153 | Serine/threonine-protein phosphatase 2A 65 kDa regulatory subunit A alpha isoform OS=Homo sapiens OX=9606 GN=PPP2R1A PE=1  SV=4 | 11 | 6 | 12 | 6 | 26,4 | 6 |
| Q14566 | DNA replication licensing factor MCM6 OS=Homo sapiens OX=9606 GN=MCM6 PE=1 SV=1 | 10 | 7 | 7 | 7 | 11,53 | 7 |
| P05362 | Intercellular adhesion molecule 1 OS=Homo sapiens OX=9606 GN=ICAM1 PE=1 SV=2 | 13 | 5 | 7 | 5 | 19,46 | 5 |
| P60228 | Eukaryotic translation initiation factor 3 subunit E OS=Homo sapiens OX=9606 GN=EIF3E PE=1 SV=1 | 14 | 6 | 8 | 6 | 16,08 | 6 |
| Q9Y230 | RuvB-like 2 OS=Homo sapiens OX=9606 GN=RUVBL2 PE=1 SV=3 | 14 | 5 | 7 | 5 | 12,35 | 5 |
| P33992 | DNA replication licensing factor MCM5 OS=Homo sapiens OX=9606 GN=MCM5 PE=1 SV=5 | 12 | 8 | 10 | 8 | 16,46 | 8 |
| Q8N1F7 | Nuclear pore complex protein Nup93 OS=Homo sapiens OX=9606 GN=NUP93 PE=1 SV=2 | 9 | 6 | 7 | 6 | 11,9 | 6 |
| Q92945 | Far upstream element-binding protein 2 OS=Homo sapiens OX=9606 GN=KHSRP PE=1 SV=4 | 8 | 4 | 6 | 3 | 17,32 | 4 |
| P36957 | Dihydrolipoyllysine-residue succinyltransferase component of 2oxoglutarate dehydrogenase complex, mitochondrial OS=Homo sapiens OX=9606 GN=DLST PE=1 SV=4 | 11 | 5 | 6 | 5 | 14,29 | 5 |
| Q9Y277 | Voltage-dependent anion-selective channel protein 3 OS=Homo sapiens OX=9606 GN=VDAC3 PE=1 SV=1 | 19 | 4 | 10 | 3 | 26,27 | 4 |
| Q969V6 | MKL/myocardin-like protein 1 OS=Homo sapiens OX=9606 GN=MKL1 PE=1 SV=1 | 6 | 4 | 6 | 4 | 17,76 | 4 |
| O75717 | WD repeat and HMG-box DNA-binding protein 1 OS=Homo sapiens OX=9606 GN=WDHD1 PE=1 SV=1 | 6 | 6 | 9 | 6 | 16,83 | 6 |

| Q12797 | Aspartyl/asparaginyl beta-hydroxylase OS=Homo sapiens OX=9606 GN=ASPH PE=1 SV=3 | 8 | 6 | 7 | 6 | 9,01 | 6 |
| --- | --- | --- | --- | --- | --- | --- | --- |
| Q9H3U1 | Protein unc-45 homolog A OS=Homo sapiens OX=9606 GN=UNC45A PE=1 SV=1 | 6 | 5 | 7 | 5 | 14,39 | 5 |
| O00303 | Eukaryotic translation initiation factor 3 subunit F OS=Homo sapiens OX=9606 GN=EIF3F PE=1 SV=1 | 15 | 4 | 6 | 4 | 17,5 | 4 |
| P51636 | Caveolin-2 OS=Homo sapiens OX=9606 GN=CAV2 PE=1 SV=2 | 21 | 2 | 4 | 2 | 12,36 | 2 |
| P15121 | Aldose reductase OS=Homo sapiens OX=9606 GN=AKR1B1 PE=1 SV=3 | 15 | 5 | 7 | 5 | 15,47 | 5 |
| Q58FG1 | Putative heat shock protein HSP 90-alpha A4 OS=Homo sapiens OX=9606 GN=HSP90AA4P PE=5 SV=1 | 7 | 3 | 18 | 1 | 51,14 | 3 |
| Q13435 | Splicing factor 3B subunit 2 OS=Homo sapiens OX=9606 GN=SF3B2 PE=1 SV=2 | 7 | 6 | 8 | 6 | 20,06 | 6 |
| P78347 | General transcription factor II-I OS=Homo sapiens OX=9606 GN=GTF2I PE=1 SV=2 | 7 | 6 | 9 | 6 | 19,22 | 6 |
| Q92598 | Heat shock protein 105 kDa OS=Homo sapiens OX=9606 GN=HSPH1 PE=1 SV=1 | 6 | 4 | 7 | 3 | 17,58 | 4 |
| P55010 | Eukaryotic translation initiation factor 5 OS=Homo sapiens OX=9606 GN=EIF5 PE=1 SV=2 | 16 | 6 | 8 | 6 | 10,14 | 6 |
| P31930 | Cytochrome b-c1 complex subunit 1, mitochondrial OS=Homo sapiens OX=9606 GN=UQCRC1 PE=1 SV=3 | 11 | 4 | 6 | 4 | 15,77 | 4 |
| P00533 | Epidermal growth factor receptor OS=Homo sapiens OX=9606 GN=EGFR PE=1 SV=2 | 5 | 6 | 7 | 6 | 9,05 | 6 |
| P54136 | Arginine--tRNA ligase, cytoplasmic OS=Homo sapiens OX=9606 GN=RARS PE=1 SV=2 | 7 | 4 | 7 | 4 | 18,21 | 4 |
| P52292 | Importin subunit alpha-1 OS=Homo sapiens OX=9606 GN=KPNA2 PE=1 SV=1 | 10 | 5 | 5 | 5 | 10,17 | 5 |
| Q13501 | Sequestosome-1 OS=Homo sapiens OX=9606 GN=SQSTM1 PE=1 SV=1 | 8 | 2 | 3 | 2 | 14,07 | 2 |
| Q04917 | 14-3-3 protein eta OS=Homo sapiens OX=9606 GN=YWHAH PE=1 SV=4 | 15 | 4 | 10 | 1 | 18,82 | 4 |
| P17858 | ATP-dependent 6-phosphofructokinase, liver type OS=Homo sapiens OX=9606 GN=PFKL PE=1 SV=6 | 6 | 4 | 5 | 2 | 12,55 | 4 |

| Q03001 | Dystonin OS=Homo sapiens OX=9606 GN=DST PE=1 SV=4 | 1 | 7 | 10 | 6 | 10,66 | 7 |
| --- | --- | --- | --- | --- | --- | --- | --- |
| P16189 | HLA class I histocompatibility antigen, A-31 alpha chain OS=Homo sapiens OX=9606 GN=HLA-A PE=1 SV=2 | 15 | 4 | 7 | 2 | 23,45 | 4 |
| P35998 | 26S proteasome regulatory subunit 7 OS=Homo sapiens OX=9606  GN=PSMC2 PE=1 SV=3 | 12 | 5 | 7 | 5 | 14,59 | 5 |
| P49915 | GMP synthase [glutamine-hydrolyzing] OS=Homo sapiens OX=9606 GN=GMPS PE=1 SV=1 | 6 | 4 | 5 | 4 | 11,17 | 4 |
| Q9Y265 | RuvB-like 1 OS=Homo sapiens OX=9606 GN=RUVBL1 PE=1 SV=1 | 10 | 4 | 7 | 4 | 18,53 | 4 |
| Q09028 | Histone-binding protein RBBP4 OS=Homo sapiens OX=9606 GN=RBBP4 PE=1 SV=3 | 17 | 5 | 7 | 5 | 9,17 | 5 |
| P28838 | Cytosol aminopeptidase OS=Homo sapiens OX=9606 GN=LAP3 PE=1 SV=3 | 9 | 4 | 6 | 4 | 13,3 | 4 |
| Q9Y2S7 | Polymerase delta-interacting protein 2 OS=Homo sapiens OX=9606 GN=POLDIP2 PE=1 SV=1 | 13 | 4 | 7 | 4 | 13,97 | 4 |
| Q96SB4 | SRSF protein kinase 1 OS=Homo sapiens OX=9606 GN=SRPK1 PE=1 SV=2 | 7 | 4 | 6 | 4 | 16,73 | 4 |
| P55209 | Nucleosome assembly protein 1-like 1 OS=Homo sapiens OX=9606 GN=NAP1L1 PE=1 SV=1 | 10 | 3 | 5 | 3 | 12,22 | 3 |
| P04179 | Superoxide dismutase [Mn], mitochondrial OS=Homo sapiens OX=9606 GN=SOD2 PE=1 SV=3 | 17 | 3 | 6 | 3 | 15,8 | 3 |
| Q07065 | Cytoskeleton-associated protein 4 OS=Homo sapiens OX=9606 GN=CKAP4 PE=1 SV=2 | 7 | 4 | 4 | 4 | 6,97 | 4 |
| P18085 | ADP-ribosylation factor 4 OS=Homo sapiens OX=9606 GN=ARF4 PE=1 SV=3 | 20 | 4 | 7 | 3 | 14,19 | 4 |
| Q99460 | 26S proteasome non-ATPase regulatory subunit 1 OS=Homo sapiens  OX=9606 GN=PSMD1 PE=1 SV=2 | 6 | 5 | 8 | 5 | 15 | 5 |
| P28838 | Cytosol aminopeptidase OS=Homo sapiens OX=9606 GN=LAP3 PE=1 SV=3 | 12 | 5 | 6 | 5 | 7,68 | 5 |
| P04439 | HLA class I histocompatibility antigen, A-3 alpha chain OS=Homo sapiens OX=9606 GN=HLA-A PE=1 SV=2 | 12 | 3 | 7 | 1 | 24,68 | 3 |
| P49848 | Transcription initiation factor TFIID subunit 6 OS=Homo sapiens OX=9606 GN=TAF6 PE=1 SV=1 | 5 | 3 | 5 | 3 | 13,33 | 3 |

| Q7Z3Y8 | Keratin, type I cytoskeletal 27 OS=Homo sapiens OX=9606 GN=KRT27 PE=1 SV=2 | 7 | 3 | 47 | 1 | 82,25 | 3 |
| --- | --- | --- | --- | --- | --- | --- | --- |
| P26006 | Integrin alpha-3 OS=Homo sapiens OX=9606 GN=ITGA3 PE=1 SV=5 | 6 | 6 | 8 | 6 | 17,56 | 6 |
| P22234 | Multifunctional protein ADE2 OS=Homo sapiens OX=9606 GN=PAICS PE=1 SV=3 | 8 | 3 | 4 | 3 | 9,56 | 3 |
| P51114 | Fragile X mental retardation syndrome-related protein 1 OS=Homo sapiens OX=9606 GN=FXR1 PE=1 SV=3 | 8 | 4 | 6 | 3 | 11,85 | 4 |
| P40763 | Signal transducer and activator of transcription 3 OS=Homo sapiens OX=9606 GN=STAT3 PE=1 SV=2 | 6 | 4 | 5 | 4 | 4,38 | 4 |
| Q13347 | Eukaryotic translation initiation factor 3 subunit I OS=Homo sapiens OX=9606 GN=EIF3I PE=1 SV=1 | 12 | 4 | 6 | 4 | 13,67 | 4 |
| Q9NSD9 | Phenylalanine--tRNA ligase beta subunit OS=Homo sapiens OX=9606 GN=FARSB PE=1 SV=3 | 7 | 4 | 6 | 4 | 11,92 | 4 |
| P54577 | Tyrosine--tRNA ligase, cytoplasmic OS=Homo sapiens OX=9606 GN=YARS PE=1 SV=4 | 12 | 5 | 6 | 5 | 13,53 | 5 |
| Q96RT1 | Erbin OS=Homo sapiens OX=9606 GN=ERBIN PE=1 SV=2 | 4 | 6 | 8 | 6 | 7,19 | 6 |
| P35637 | RNA-binding protein FUS OS=Homo sapiens OX=9606 GN=FUS PE=1 SV=1 | 9 | 3 | 7 | 1 | 16,42 | 3 |
| P27695 | DNA-(apurinic or apyrimidinic site) lyase OS=Homo sapiens OX=9606 GN=APEX1 PE=1 SV=2 | 9 | 2 | 2 | 2 | 6,69 | 2 |
| P37802 | Transgelin-2 OS=Homo sapiens OX=9606 GN=TAGLN2 PE=1 SV=3 | 24 | 5 | 7 | 5 | 15,46 | 5 |
| P16152 | Carbonyl reductase [NADPH] 1 OS=Homo sapiens OX=9606 GN=CBR1 PE=1 SV=3 | 16 | 3 | 4 | 3 | 6,39 | 3 |
| O75533 | Splicing factor 3B subunit 1 OS=Homo sapiens OX=9606 GN=SF3B1 PE=1 SV=3 | 2 | 3 | 6 | 3 | 18,38 | 3 |
| Q15019 | Septin-2 OS=Homo sapiens OX=9606 GN=SEPT2 PE=1 SV=1 | 13 | 4 | 6 | 4 | 7,17 | 4 |
| P53618 | Coatomer subunit beta OS=Homo sapiens OX=9606 GN=COPB1 PE=1 SV=3 | 7 | 5 | 6 | 5 | 13,53 | 5 |
| P43243 | Matrin-3 OS=Homo sapiens OX=9606 GN=MATR3 PE=1 SV=2 | 4 | 3 | 4 | 3 | 10,91 | 3 |
| P27694 | Replication protein A 70 kDa DNA-binding subunit OS=Homo sapiens OX=9606 GN=RPA1 PE=1 SV=2 | 7 | 5 | 8 | 5 | 10,14 | 5 |
| P16070 | CD44 antigen OS=Homo sapiens OX=9606 GN=CD44 PE=1 SV=3 | 4 | 3 | 5 | 3 | 12,57 | 3 |

| P36776 | Lon protease homolog, mitochondrial OS=Homo sapiens OX=9606 GN=LONP1 PE=1 SV=2 | 5 | 4 | 4 | 4 | 5,07 | 4 |
| --- | --- | --- | --- | --- | --- | --- | --- |
| Q92688 | Acidic leucine-rich nuclear phosphoprotein 32 family member B OS=Homo sapiens OX=9606 GN=ANP32B PE=1 SV=1 | 14 | 3 | 4 | 2 | 6,97 | 3 |
| Q99459 | Cell division cycle 5-like protein OS=Homo sapiens OX=9606 GN=CDC5L PE=1 SV=2 | 5 | 4 | 4 | 4 | 8,28 | 4 |
| P46782 | 40S ribosomal protein S5 OS=Homo sapiens OX=9606 GN=RPS5 PE=1 SV=4 | 15 | 4 | 6 | 4 | 14,78 | 4 |
| Q8WVM8 | Sec1 family domain-containing protein 1 OS=Homo sapiens OX=9606 GN=SCFD1 PE=1 SV=4 | 7 | 4 | 5 | 4 | 10,87 | 4 |
| Q7L576 | Cytoplasmic FMR1-interacting protein 1 OS=Homo sapiens OX=9606 GN=CYFIP1 PE=1 SV=1 | 4 | 4 | 4 | 4 | 9,85 | 4 |
| Q9NSE4 | Isoleucine--tRNA ligase, mitochondrial OS=Homo sapiens OX=9606 GN=IARS2 PE=1 SV=2 | 5 | 4 | 5 | 4 | 11,9 | 4 |
| P29317 | Ephrin type-A receptor 2 OS=Homo sapiens OX=9606 GN=EPHA2 PE=1 SV=2 | 5 | 4 | 4 | 4 | 8,43 | 4 |
| Q96P70 | Importin-9 OS=Homo sapiens OX=9606 GN=IPO9 PE=1 SV=3 | 4 | 4 | 6 | 4 | 9,98 | 4 |
| P36873 | Serine/threonine-protein phosphatase PP1-gamma catalytic subunit OS=Homo sapiens OX=9606 GN=PPP1CC PE=1 SV=1 | 14 | 4 | 5 | 1 | 5,86 | 4 |
| P50454 | Serpin H1 OS=Homo sapiens OX=9606 GN=SERPINH1 PE=1 SV=2 | 11 | 4 | 5 | 4 | 8,45 | 4 |
| Q32MZ4 | Leucine-rich repeat flightless-interacting protein 1 OS=Homo sapiens OX=9606 GN=LRRFIP1 PE=1 SV=2 | 5 | 3 | 4 | 3 | 7,71 | 3 |
| P33991 | DNA replication licensing factor MCM4 OS=Homo sapiens OX=9606 GN=MCM4 PE=1 SV=5 | 4 | 3 | 4 | 3 | 10,98 | 3 |
| P11387 | DNA topoisomerase 1 OS=Homo sapiens OX=9606 GN=TOP1 PE=1 SV=2 | 5 | 4 | 5 | 4 | 10,84 | 4 |
| P61353 | 60S ribosomal protein L27 OS=Homo sapiens OX=9606 GN=RPL27 PE=1 SV=2 | 36 | 4 | 6 | 4 | 11,05 | 4 |
| P52565 | Rho GDP-dissociation inhibitor 1 OS=Homo sapiens OX=9606 GN=ARHGDIA PE=1 SV=3 | 16 | 3 | 4 | 3 | 11 | 3 |
| Q7L1Q6 | Basic leucine zipper and W2 domain-containing protein 1 OS=Homo sapiens OX=9606 GN=BZW1 PE=1 SV=1 | 10 | 5 | 9 | 4 | 14,45 | 5 |

| P62750 | 60S ribosomal protein L23a OS=Homo sapiens OX=9606 GN=RPL23A PE=1 SV=1 | 28 | 4 | 6 | 4 | 12,08 | 4 |
| --- | --- | --- | --- | --- | --- | --- | --- |
| Q13263 | Transcription intermediary factor 1-beta OS=Homo sapiens OX=9606 GN=TRIM28 PE=1 SV=5 | 5 | 4 | 9 | 4 | 15,69 | 4 |
| P55263 | Adenosine kinase OS=Homo sapiens OX=9606 GN=ADK PE=1 SV=2 | 12 | 4 | 6 | 4 | 11,02 | 4 |
| Q9Y617 | Phosphoserine aminotransferase OS=Homo sapiens OX=9606 GN=PSAT1 PE=1 SV=2 | 11 | 4 | 7 | 4 | 13,63 | 4 |
| P05455 | Lupus La protein OS=Homo sapiens OX=9606 GN=SSB PE=1 SV=2 | 7 | 3 | 4 | 3 | 8,51 | 3 |
| P09429 | High mobility group protein B1 OS=Homo sapiens OX=9606 GN=HMGB1 PE=1 SV=3 | 22 | 3 | 3 | 3 | 8,86 | 3 |
| Q96G23 | Ceramide synthase 2 OS=Homo sapiens OX=9606 GN=CERS2 PE=1 SV=1 | 8 | 2 | 3 | 2 | 6,27 | 2 |
| Q96HE7 | ERO1-like protein alpha OS=Homo sapiens OX=9606 GN=ERO1A PE=1 SV=2 | 7 | 3 | 4 | 3 | 9,94 | 3 |
| P17301 | Integrin alpha-2 OS=Homo sapiens OX=9606 GN=ITGA2 PE=1 SV=1 | 4 | 5 | 7 | 5 | 12,67 | 5 |
| O14979 | Heterogeneous nuclear ribonucleoprotein D-like OS=Homo sapiens OX=9606 GN=HNRNPDL PE=1 SV=3 | 8 | 3 | 5 | 2 | 12,62 | 3 |
| P04632 | Calpain small subunit 1 OS=Homo sapiens OX=9606 GN=CAPNS1 PE=1 SV=1 | 13 | 4 | 7 | 4 | 11,9 | 4 |
| Q9UHB9 | Signal recognition particle subunit SRP68 OS=Homo sapiens OX=9606 GN=SRP68 PE=1 SV=2 | 7 | 4 | 6 | 4 | 12,84 | 4 |
| O75665 | Oral-facial-digital syndrome 1 protein OS=Homo sapiens OX=9606 GN=OFD1 PE=1 SV=1 | 4 | 3 | 3 | 3 | 4,14 | 3 |
| O75694 | Nuclear pore complex protein Nup155 OS=Homo sapiens OX=9606 GN=NUP155 PE=1 SV=1 | 2 | 3 | 3 | 3 | 4,57 | 3 |
| O14744 | Protein arginine N-methyltransferase 5 OS=Homo sapiens OX=9606 GN=PRMT5 PE=1 SV=4 | 5 | 3 | 5 | 3 | 11,54 | 3 |
| Q9H223 | EH domain-containing protein 4 OS=Homo sapiens OX=9606 GN=EHD4 PE=1 SV=1 | 6 | 3 | 4 | 3 | 9,69 | 3 |
| P22531 | Small proline-rich protein 2E OS=Homo sapiens OX=9606 GN=SPRR2E PE=2 SV=2 | 61 | 3 | 10 | 3 | 24,76 | 3 |
| P46977 | Dolichyl-diphosphooligosaccharide--protein glycosyltransferase subunit STT3A OS=Homo sapiens OX=9606 GN=STT3A PE=1 SV=2 | 5 | 3 | 5 | 3 | 9,27 | 3 |

| P42166 | Lamina-associated polypeptide 2, isoform alpha OS=Homo sapiens OX=9606 GN=TMPO PE=1 SV=2 | 6 | 3 | 3 | 3 | 7,1 | 3 |
| --- | --- | --- | --- | --- | --- | --- | --- |
| Q9NYF8 | Bcl-2-associated transcription factor 1 OS=Homo sapiens OX=9606 GN=BCLAF1 PE=1 SV=2 | 5 | 4 | 6 | 4 | 9,75 | 4 |
| Q00341 | Vigilin OS=Homo sapiens OX=9606 GN=HDLBP PE=1 SV=2 | 4 | 4 | 6 | 4 | 11,64 | 4 |
| Q9NVI7 | ATPase family AAA domain-containing protein 3A OS=Homo sapiens OX=9606 GN=ATAD3A PE=1 SV=2 | 7 | 5 | 7 | 5 | 12,78 | 5 |
| P50570 | Dynamin-2 OS=Homo sapiens OX=9606 GN=DNM2 PE=1 SV=2 | 5 | 4 | 5 | 4 | 8,64 | 4 |
| P52732 | Kinesin-like protein KIF11 OS=Homo sapiens OX=9606 GN=KIF11 PE=1 SV=2 | 4 | 3 | 3 | 3 | 7,97 | 3 |
| Q14157 | Ubiquitin-associated protein 2-like OS=Homo sapiens OX=9606 GN=UBAP2L PE=1 SV=2 | 3 | 2 | 3 | 2 | 9,33 | 2 |
| O76003 | Glutaredoxin-3 OS=Homo sapiens OX=9606 GN=GLRX3 PE=1 SV=2 | 13 | 4 | 5 | 4 | 9,35 | 4 |
| P04844 | Dolichyl-diphosphooligosaccharide--protein glycosyltransferase subunit 2 OS=Homo sapiens OX=9606 GN=RPN2 PE=1 SV=3 | 5 | 3 | 5 | 3 | 8,24 | 3 |
| P46777 | 60S ribosomal protein L5 OS=Homo sapiens OX=9606 GN=RPL5 PE=1 SV=3 | 8 | 2 | 3 | 2 | 5,71 | 2 |
| Q02543 | 60S ribosomal protein L18a OS=Homo sapiens OX=9606 GN=RPL18A PE=1 SV=2 | 17 | 3 | 3 | 3 | 7,26 | 3 |
| Q08945 | FACT complex subunit SSRP1 OS=Homo sapiens OX=9606 GN=SSRP1 PE=1 SV=1 | 4 | 3 | 4 | 3 | 6,91 | 3 |
| P35659 | Protein DEK OS=Homo sapiens OX=9606 GN=DEK PE=1 SV=1 | 9 | 3 | 3 | 3 | 4,97 | 3 |
| Q13247 | Serine/arginine-rich splicing factor 6 OS=Homo sapiens OX=9606 GN=SRSF6 PE=1 SV=2 | 9 | 3 | 5 | 3 | 12,26 | 3 |
| P30040 | Endoplasmic reticulum resident protein 29 OS=Homo sapiens OX=9606 GN=ERP29 PE=1 SV=4 | 14 | 3 | 4 | 3 | 8,45 | 3 |
| Q9BYT8 | Neurolysin, mitochondrial OS=Homo sapiens OX=9606 GN=NLN PE=1 SV=1 | 4 | 3 | 4 | 3 | 4,7 | 3 |
| Q9UMS4 | Pre-mRNA-processing factor 19 OS=Homo sapiens OX=9606 GN=PRPF19 PE=1 SV=1 | 7 | 3 | 4 | 3 | 8,59 | 3 |
| P36871 | Phosphoglucomutase-1 OS=Homo sapiens OX=9606 GN=PGM1 PE=1 SV=3 | 6 | 3 | 3 | 3 | 4,98 | 3 |

| P49321 | Nuclear autoantigenic sperm protein OS=Homo sapiens OX=9606 GN=NASP PE=1 SV=2 | 4 | 3 | 4 | 3 | 9,53 | 3 |
| --- | --- | --- | --- | --- | --- | --- | --- |
| P23381 | Tryptophan--tRNA ligase, cytoplasmic OS=Homo sapiens OX=9606 GN=WARS PE=1 SV=2 | 6 | 2 | 2 | 2 | 5,22 | 2 |
| P46060 | Ran GTPase-activating protein 1 OS=Homo sapiens OX=9606 GN=RANGAP1 PE=1 SV=1 | 4 | 2 | 3 | 2 | 8,35 | 2 |
| Q15645 | Pachytene checkpoint protein 2 homolog OS=Homo sapiens OX=9606 GN=TRIP13 PE=1 SV=2 | 5 | 2 | 3 | 2 | 8,2 | 2 |
| P51116 | Fragile X mental retardation syndrome-related protein 2 OS=Homo sapiens OX=9606 GN=FXR2 PE=1 SV=2 | 4 | 2 | 3 | 1 | 3,15 | 2 |
| O15371 | Eukaryotic translation initiation factor 3 subunit D OS=Homo sapiens OX=9606 GN=EIF3D PE=1 SV=1 | 6 | 4 | 5 | 4 | 7,59 | 4 |
| Q9UG63 | ATP-binding cassette sub-family F member 2 OS=Homo sapiens OX=9606 GN=ABCF2 PE=1 SV=2 | 5 | 3 | 3 | 3 | 6,81 | 3 |
| Q92900 | Regulator of nonsense transcripts 1 OS=Homo sapiens OX=9606 GN=UPF1 PE=1 SV=2 | 4 | 4 | 6 | 4 | 6,64 | 4 |
| Q14126 | Desmoglein-2 OS=Homo sapiens OX=9606 GN=DSG2 PE=1 SV=2 | 3 | 2 | 4 | 2 | 10,59 | 2 |
| Q9BY44 | Eukaryotic translation initiation factor 2A OS=Homo sapiens OX=9606 GN=EIF2A PE=1 SV=3 | 6 | 3 | 3 | 3 | 7,25 | 3 |
| O60749 | Sorting nexin-2 OS=Homo sapiens OX=9606 GN=SNX2 PE=1 SV=2 | 5 | 2 | 2 | 2 | 5,01 | 2 |
| O15355 | Protein phosphatase 1G OS=Homo sapiens OX=9606 GN=PPM1G PE=1 SV=1 | 4 | 2 | 2 | 2 | 2,92 | 2 |
| O95714 | E3 ubiquitin-protein ligase HERC2 OS=Homo sapiens OX=9606 GN=HERC2 PE=1 SV=2 | 1 | 3 | 4 | 3 | 5,58 | 3 |
| Q99623 | Prohibitin-2 OS=Homo sapiens OX=9606 GN=PHB2 PE=1 SV=2 | 13 | 4 | 5 | 4 | 9,22 | 4 |
| Q9UQE7 | Structural maintenance of chromosomes protein 3 OS=Homo sapiens OX=9606 GN=SMC3 PE=1 SV=2 | 2 | 2 | 3 | 2 | 4,96 | 2 |
| P07858 | Cathepsin B OS=Homo sapiens OX=9606 GN=CTSB PE=1 SV=3 | 10 | 3 | 3 | 3 | 6,08 | 3 |
| P46063 | ATP-dependent DNA helicase Q1 OS=Homo sapiens OX=9606 GN=RECQL PE=1 SV=3 | 5 | 3 | 5 | 3 | 12,1 | 3 |
| P21108 | Ribose-phosphate pyrophosphokinase 3 OS=Homo sapiens OX=9606 GN=PRPS1L1 PE=1 SV=2 | 8 | 2 | 4 | 2 | 9,78 | 2 |

| Q15436 | Protein transport protein Sec23A OS=Homo sapiens OX=9606 GN=SEC23A PE=1 SV=2 | 3 | 2 | 3 | 2 | 6,86 | 2 |
| --- | --- | --- | --- | --- | --- | --- | --- |
| P30419 | Glycylpeptide N-tetradecanoyltransferase 1 OS=Homo sapiens OX=9606 GN=NMT1 PE=1 SV=2 | 6 | 3 | 5 | 3 | 7,32 | 3 |
| P17174 | Aspartate aminotransferase, cytoplasmic OS=Homo sapiens OX=9606 GN=GOT1 PE=1 SV=3 | 9 | 3 | 4 | 3 | 7,6 | 3 |
| P08195 | 4F2 cell-surface antigen heavy chain OS=Homo sapiens OX=9606  GN=SLC3A2 PE=1 SV=3 | 5 | 3 | 4 | 3 | 9,29 | 3 |
| P63241 | Eukaryotic translation initiation factor 5A-1 OS=Homo sapiens OX=9606 GN=EIF5A PE=1 SV=2 | 16 | 2 | 3 | 2 | 7,7 | 2 |
| P62888 | 60S ribosomal protein L30 OS=Homo sapiens OX=9606 GN=RPL30 PE=1 SV=2 | 24 | 2 | 2 | 2 | 6,23 | 2 |
| Q15459 | Splicing factor 3A subunit 1 OS=Homo sapiens OX=9606 GN=SF3A1 PE=1 SV=1 | 6 | 3 | 3 | 3 | 4,21 | 3 |
| P12004 | Proliferating cell nuclear antigen OS=Homo sapiens OX=9606 GN=PCNA PE=1 SV=1 | 8 | 2 | 3 | 2 | 7,24 | 2 |
| Q02241 | Kinesin-like protein KIF23 OS=Homo sapiens OX=9606 GN=KIF23 PE=1 SV=3 | 3 | 2 | 2 | 2 | 5,12 | 2 |
| Q15185 | Prostaglandin E synthase 3 OS=Homo sapiens OX=9606 GN=PTGES3 PE=1 SV=1 | 19 | 2 | 3 | 2 | 5,34 | 2 |
| O00232 | 26S proteasome non-ATPase regulatory subunit 12 OS=Homo sapiens OX=9606 GN=PSMD12 PE=1 SV=3 | 5 | 2 | 3 | 2 | 6,76 | 2 |
| Q13617 | Cullin-2 OS=Homo sapiens OX=9606 GN=CUL2 PE=1 SV=2 | 4 | 4 | 5 | 3 | 7,82 | 4 |
| Q07955 | Serine/arginine-rich splicing factor 1 OS=Homo sapiens OX=9606 GN=SRSF1 PE=1 SV=2 | 12 | 3 | 3 | 3 | 5,51 | 3 |
| Q16543 | Hsp90 co-chaperone Cdc37 OS=Homo sapiens OX=9606 GN=CDC37 PE=1 SV=1 | 7 | 2 | 4 | 2 | 10,9 | 2 |
| Q96AE4 | Far upstream element-binding protein 1 OS=Homo sapiens OX=9606 GN=FUBP1 PE=1 SV=3 | 5 | 3 | 4 | 2 | 7,58 | 3 |
| Q15691 | Microtubule-associated protein RP/EB family member 1 OS=Homo sapiens OX=9606 GN=MAPRE1 PE=1 SV=3 | 10 | 2 | 3 | 2 | 7,08 | 2 |
| O43747 | AP-1 complex subunit gamma-1 OS=Homo sapiens OX=9606 GN=AP1G1 PE=1 SV=5 | 4 | 3 | 4 | 3 | 6,5 | 3 |

| P35613 | Basigin OS=Homo sapiens OX=9606 GN=BSG PE=1 SV=2 | 5 | 1 | 2 | 1 | 7,07 | 1 |
| --- | --- | --- | --- | --- | --- | --- | --- |
| Q92499 | ATP-dependent RNA helicase DDX1 OS=Homo sapiens OX=9606 GN=DDX1 PE=1 SV=2 | 3 | 2 | 4 | 2 | 9,79 | 2 |
| P54578 | Ubiquitin carboxyl-terminal hydrolase 14 OS=Homo sapiens OX=9606 GN=USP14 PE=1 SV=3 | 6 | 3 | 4 | 3 | 6,17 | 3 |
| Q96FW1 | Ubiquitin thioesterase OTUB1 OS=Homo sapiens OX=9606 GN=OTUB1 PE=1 SV=2 | 13 | 3 | 3 | 3 | 6,54 | 3 |
| P46781 | 40S ribosomal protein S9 OS=Homo sapiens OX=9606 GN=RPS9 PE=1 SV=3 | 18 | 4 | 7 | 4 | 12,47 | 4 |
| O95433 | Activator of 90 kDa heat shock protein ATPase homolog 1 OS=Homo sapiens OX=9606 GN=AHSA1 PE=1 SV=1 | 7 | 2 | 3 | 2 | 7,43 | 2 |
| Q9NR12 | PDZ and LIM domain protein 7 OS=Homo sapiens OX=9606 GN=PDLIM7 PE=1 SV=1 | 7 | 3 | 3 | 3 | 4,71 | 3 |
| P49756 | RNA-binding protein 25 OS=Homo sapiens OX=9606 GN=RBM25 PE=1 SV=3 | 2 | 1 | 2 | 1 | 6,78 | 1 |
| O75153 | Clustered mitochondria protein homolog OS=Homo sapiens OX=9606 GN=CLUH PE=1 SV=2 | 2 | 2 | 3 | 2 | 5,51 | 2 |
| P62495 | Eukaryotic peptide chain release factor subunit 1 OS=Homo sapiens OX=9606 GN=ETF1 PE=1 SV=3 | 7 | 3 | 5 | 3 | 7,34 | 3 |
| O43684 | Mitotic checkpoint protein BUB3 OS=Homo sapiens OX=9606 GN=BUB3 PE=1 SV=1 | 6 | 2 | 2 | 2 | 5,08 | 2 |
| Q9NR30 | Nucleolar RNA helicase 2 OS=Homo sapiens OX=9606 GN=DDX21 PE=1 SV=5 | 5 | 3 | 4 | 3 | 9,57 | 3 |
| Q8N684 | Cleavage and polyadenylation specificity factor subunit 7 OS=Homo sapiens OX=9606 GN=CPSF7 PE=1 SV=1 | 6 | 3 | 4 | 3 | 5,89 | 3 |
| A5YKK6 | CCR4-NOT transcription complex subunit 1 OS=Homo sapiens OX=9606 GN=CNOT1 PE=1 SV=2 | 2 | 4 | 5 | 4 | 8,39 | 4 |
| Q7RTP6 | [F-actin]-monooxygenase MICAL3 OS=Homo sapiens OX=9606  GN=MICAL3 PE=1 SV=2 | 1 | 2 | 2 | 2 | 4,44 | 2 |
| P10620 | Microsomal glutathione S-transferase 1 OS=Homo sapiens OX=9606 GN=MGST1 PE=1 SV=1 | 19 | 2 | 3 | 2 | 8,29 | 2 |
| Q9P035 | Very-long-chain (3R)-3-hydroxyacyl-CoA dehydratase 3 OS=Homo sapiens OX=9606 GN=HACD3 PE=1 SV=2 | 7 | 2 | 2 | 2 | 6,43 | 2 |

| Q92769 | Histone deacetylase 2 OS=Homo sapiens OX=9606 GN=HDAC2 PE=1 SV=2 | 6 | 3 | 6 | 3 | 10,24 | 3 |
| --- | --- | --- | --- | --- | --- | --- | --- |
| Q9BTT0 | Acidic leucine-rich nuclear phosphoprotein 32 family member E OS=Homo sapiens OX=9606 GN=ANP32E PE=1 SV=1 | 5 | 2 | 3 | 2 | 8,06 | 2 |
| Q01105 | Protein SET OS=Homo sapiens OX=9606 GN=SET PE=1 SV=3 | 8 | 2 | 3 | 2 | 8,62 | 2 |
| O60524 | Nuclear export mediator factor NEMF OS=Homo sapiens OX=9606 GN=NEMF PE=1 SV=4 | 3 | 3 | 3 | 3 | 5,45 | 3 |
| P33993 | DNA replication licensing factor MCM7 OS=Homo sapiens OX=9606 GN=MCM7 PE=1 SV=4 | 4 | 3 | 5 | 3 | 10,78 | 3 |
| P61313 | 60S ribosomal protein L15 OS=Homo sapiens OX=9606 GN=RPL15 PE=1 SV=2 | 16 | 3 | 6 | 3 | 8,11 | 3 |
| P62424 | 60S ribosomal protein L7a OS=Homo sapiens OX=9606 GN=RPL7A PE=1 SV=2 | 9 | 2 | 3 | 2 | 7,84 | 2 |
| O43491 | Band 4.1-like protein 2 OS=Homo sapiens OX=9606 GN=EPB41L2 PE=1 SV=1 | 2 | 2 | 2 | 2 | 5,38 | 2 |
| P50502 | Hsc70-interacting protein OS=Homo sapiens OX=9606 GN=ST13 PE=1 SV=2 | 6 | 2 | 3 | 2 | 7,1 | 2 |
| Q9P0L0 | Vesicle-associated membrane protein-associated protein A OS=Homo sapiens OX=9606 GN=VAPA PE=1 SV=3 | 11 | 2 | 3 | 2 | 6,73 | 2 |
| O95817 | BAG family molecular chaperone regulator 3 OS=Homo sapiens OX=9606 GN=BAG3 PE=1 SV=3 | 4 | 2 | 2 | 2 | 2,74 | 2 |
| P02794 | Ferritin heavy chain OS=Homo sapiens OX=9606 GN=FTH1 PE=1 SV=2 | 17 | 3 | 4 | 3 | 0 | 3 |
| P63000 | Ras-related C3 botulinum toxin substrate 1 OS=Homo sapiens OX=9606 GN=RAC1 PE=1 SV=1 | 13 | 2 | 2 | 2 | 4,71 | 2 |
| P62333 | 26S proteasome regulatory subunit 10B OS=Homo sapiens OX=9606  GN=PSMC6 PE=1 SV=1 | 6 | 2 | 2 | 2 | 5,07 | 2 |
| Q15717 | ELAV-like protein 1 OS=Homo sapiens OX=9606 GN=ELAVL1 PE=1 SV=2 | 6 | 2 | 3 | 2 | 5,02 | 2 |
| Q5JTV8 | Torsin-1A-interacting protein 1 OS=Homo sapiens OX=9606 GN=TOR1AIP1 PE=1 SV=2 | 4 | 2 | 3 | 2 | 7,78 | 2 |
| Q9Y2W1 | Thyroid hormone receptor-associated protein 3 OS=Homo sapiens OX=9606 GN=THRAP3 PE=1 SV=2 | 2 | 2 | 3 | 2 | 7,86 | 2 |

| P42285 | Exosome RNA helicase MTR4 OS=Homo sapiens OX=9606 GN=MTREX PE=1 SV=3 | 3 | 3 | 3 | 3 | 6,6 | 3 |
| --- | --- | --- | --- | --- | --- | --- | --- |
| O95347 | Structural maintenance of chromosomes protein 2 OS=Homo sapiens OX=9606 GN=SMC2 PE=1 SV=2 | 2 | 2 | 2 | 2 | 2,05 | 2 |
| Q9Y3I0 | tRNA-splicing ligase RtcB homolog OS=Homo sapiens OX=9606 GN=RTCB PE=1 SV=1 | 6 | 3 | 3 | 3 | 3,99 | 3 |
| Q9UHD8 | Septin-9 OS=Homo sapiens OX=9606 GN=SEPT9 PE=1 SV=2 | 7 | 3 | 4 | 3 | 6,41 | 3 |
| Q99829 | Copine-1 OS=Homo sapiens OX=9606 GN=CPNE1 PE=1 SV=1 | 5 | 3 | 4 | 3 | 4,33 | 3 |
| P55036 | 26S proteasome non-ATPase regulatory subunit 4 OS=Homo sapiens  OX=9606 GN=PSMD4 PE=1 SV=1 | 7 | 2 | 2 | 2 | 2,36 | 2 |
| P08243 | Asparagine synthetase [glutamine-hydrolyzing] OS=Homo sapiens OX=9606 GN=ASNS PE=1 SV=4 | 5 | 3 | 5 | 3 | 1,86 | 3 |
| P60866 | 40S ribosomal protein S20 OS=Homo sapiens OX=9606 GN=RPS20 PE=1 SV=1 | 23 | 2 | 3 | 2 | 8,44 | 2 |
| O43399 | Tumor protein D54 OS=Homo sapiens OX=9606 GN=TPD52L2 PE=1 SV=2 | 11 | 2 | 2 | 2 | 5,74 | 2 |
| Q14739 | Lamin-B receptor OS=Homo sapiens OX=9606 GN=LBR PE=1 SV=2 | 4 | 2 | 2 | 2 | 4,37 | 2 |
| Q8TB61 | Adenosine 3'-phospho 5'-phosphosulfate transporter 1 OS=Homo sapiens OX=9606 GN=SLC35B2 PE=1 SV=1 | 5 | 2 | 3 | 2 | 5,4 | 2 |
| Q9H6R4 | Nucleolar protein 6 OS=Homo sapiens OX=9606 GN=NOL6 PE=1 SV=2 | 2 | 2 | 4 | 2 | 8,22 | 2 |
| Q8TAQ2 | SWI/SNF complex subunit SMARCC2 OS=Homo sapiens OX=9606 GN=SMARCC2 PE=1 SV=1 | 2 | 2 | 3 | 2 | 3,76 | 2 |
| Q92888 | Rho guanine nucleotide exchange factor 1 OS=Homo sapiens OX=9606 GN=ARHGEF1 PE=1 SV=2 | 3 | 2 | 2 | 2 | 5,82 | 2 |
| P17980 | 26S proteasome regulatory subunit 6A OS=Homo sapiens OX=9606  GN=PSMC3 PE=1 SV=3 | 9 | 3 | 5 | 3 | 9,89 | 3 |
| Q5VYK3 | Proteasome adapter and scaffold protein ECM29 OS=Homo sapiens OX=9606 GN=ECPAS PE=1 SV=2 | 1 | 2 | 3 | 2 | 2,37 | 2 |
| O00268 | Transcription initiation factor TFIID subunit 4 OS=Homo sapiens OX=9606 GN=TAF4 PE=1 SV=2 | 2 | 2 | 3 | 2 | 5,65 | 2 |
| P22223 | Cadherin-3 OS=Homo sapiens OX=9606 GN=CDH3 PE=1 SV=2 | 3 | 2 | 3 | 2 | 5,73 | 2 |
| Q13162 | Peroxiredoxin-4 OS=Homo sapiens OX=9606 GN=PRDX4 PE=1 SV=1 | 7 | 2 | 4 | 1 | 9,17 | 2 |

| Q8N163 | Cell cycle and apoptosis regulator protein 2 OS=Homo sapiens OX=9606 GN=CCAR2 PE=1 SV=2 | 3 | 2 | 2 | 2 | 4,78 | 2 |
| --- | --- | --- | --- | --- | --- | --- | --- |
| P51858 | Hepatoma-derived growth factor OS=Homo sapiens OX=9606 GN=HDGF PE=1 SV=1 | 8 | 2 | 3 | 2 | 6,26 | 2 |
| Q99798 | Aconitate hydratase, mitochondrial OS=Homo sapiens OX=9606 GN=ACO2 PE=1 SV=2 | 4 | 2 | 3 | 2 | 8,68 | 2 |
| P25398 | 40S ribosomal protein S12 OS=Homo sapiens OX=9606 GN=RPS12 PE=1 SV=3 | 20 | 3 | 4 | 3 | 1,69 | 3 |
| Q92896 | Golgi apparatus protein 1 OS=Homo sapiens OX=9606 GN=GLG1 PE=1 SV=2 | 1 | 1 | 1 | 1 | 3,25 | 1 |
| P35080 | Profilin-2 OS=Homo sapiens OX=9606 GN=PFN2 PE=1 SV=3 | 10 | 1 | 1 | 1 | 3,37 | 1 |
| Q13033 | Striatin-3 OS=Homo sapiens OX=9606 GN=STRN3 PE=1 SV=3 | 3 | 2 | 2 | 2 | 5,11 | 2 |
| P31689 | DnaJ homolog subfamily A member 1 OS=Homo sapiens OX=9606 GN=DNAJA1 PE=1 SV=2 | 3 | 1 | 2 | 1 | 4,98 | 1 |
| P30044 | Peroxiredoxin-5, mitochondrial OS=Homo sapiens OX=9606 GN=PRDX5 PE=1 SV=4 | 9 | 2 | 2 | 2 | 3,66 | 2 |
| Q8IYB3 | Serine/arginine repetitive matrix protein 1 OS=Homo sapiens OX=9606 GN=SRRM1 PE=1 SV=2 | 3 | 2 | 3 | 2 | 5,17 | 2 |
| P22061 | Protein-L-isoaspartate(D-aspartate) O-methyltransferase OS=Homo sapiens OX=9606 GN=PCMT1 PE=1 SV=4 | 6 | 1 | 2 | 1 | 3,24 | 1 |
| O15427 | Monocarboxylate transporter 4 OS=Homo sapiens OX=9606 GN=SLC16A3 PE=1 SV=1 | 5 | 2 | 3 | 2 | 3,31 | 2 |
| Q9UBT2 | SUMO-activating enzyme subunit 2 OS=Homo sapiens OX=9606 GN=UBA2 PE=1 SV=2 | 3 | 2 | 3 | 2 | 6,25 | 2 |
| Q7L2E3 | Putative ATP-dependent RNA helicase DHX30 OS=Homo sapiens OX=9606 GN=DHX30 PE=1 SV=1 | 2 | 2 | 2 | 2 | 3,97 | 2 |
| Q9Y5K6 | CD2-associated protein OS=Homo sapiens OX=9606 GN=CD2AP PE=1 SV=1 | 5 | 3 | 5 | 3 | 5,86 | 3 |
| P48444 | Coatomer subunit delta OS=Homo sapiens OX=9606 GN=ARCN1 PE=1 SV=1 | 4 | 2 | 3 | 2 | 6,36 | 2 |
| Q9NZB2 | Constitutive coactivator of PPAR-gamma-like protein 1 OS=Homo sapiens OX=9606 GN=FAM120A PE=1 SV=2 | 3 | 2 | 2 | 2 | 2,8 | 2 |

| Q96DN5 | TBC1 domain family member 31 OS=Homo sapiens OX=9606 GN=TBC1D31 PE=1 SV=2 | 2 | 2 | 2 | 2 | 3,98 | 2 |
| --- | --- | --- | --- | --- | --- | --- | --- |
| P04181 | Ornithine aminotransferase, mitochondrial OS=Homo sapiens OX=9606 GN=OAT PE=1 SV=1 | 7 | 3 | 5 | 3 | 7,67 | 3 |
| P31040 | Succinate dehydrogenase [ubiquinone] flavoprotein subunit, mitochondrial OS=Homo sapiens OX=9606 GN=SDHA PE=1 SV=2 | 2 | 1 | 1 | 1 | 3,28 | 1 |
| Q9Y2A7 | Nck-associated protein 1 OS=Homo sapiens OX=9606 GN=NCKAP1 PE=1 SV=1 | 2 | 2 | 2 | 2 | 4,74 | 2 |
| Q13492 | Phosphatidylinositol-binding clathrin assembly protein OS=Homo sapiens OX=9606 GN=PICALM PE=1 SV=2 | 4 | 2 | 4 | 2 | 6,85 | 2 |
| Q9UHX1 | Poly(U)-binding-splicing factor PUF60 OS=Homo sapiens OX=9606 GN=PUF60 PE=1 SV=1 | 6 | 3 | 3 | 3 | 3,4 | 3 |
| Q9NR45 | Sialic acid synthase OS=Homo sapiens OX=9606 GN=NANS PE=1 SV=2 | 5 | 1 | 1 | 1 | 3,45 | 1 |
| Q9NZ08 | Endoplasmic reticulum aminopeptidase 1 OS=Homo sapiens OX=9606 GN=ERAP1 PE=1 SV=3 | 2 | 2 | 4 | 2 | 6,3 | 2 |
| O75821 | Eukaryotic translation initiation factor 3 subunit G OS=Homo sapiens OX=9606 GN=EIF3G PE=1 SV=2 | 4 | 1 | 2 | 1 | 6,48 | 1 |
| P30740 | Leukocyte elastase inhibitor OS=Homo sapiens OX=9606 GN=SERPINB1 PE=1 SV=1 | 5 | 2 | 3 | 2 | 1,85 | 2 |
| Q86TI2 | Dipeptidyl peptidase 9 OS=Homo sapiens OX=9606 GN=DPP9 PE=1 SV=3 | 3 | 3 | 5 | 3 | 6,03 | 3 |
| Q13428 | Treacle protein OS=Homo sapiens OX=9606 GN=TCOF1 PE=1 SV=3 | 2 | 2 | 2 | 2 | 4,35 | 2 |
| Q9UNH7 | Sorting nexin-6 OS=Homo sapiens OX=9606 GN=SNX6 PE=1 SV=1 | 5 | 2 | 2 | 2 | 2,75 | 2 |
| P43686 | 26S proteasome regulatory subunit 6B OS=Homo sapiens OX=9606  GN=PSMC4 PE=1 SV=2 | 5 | 2 | 3 | 2 | 2,76 | 2 |
| Q01844 | RNA-binding protein EWS OS=Homo sapiens OX=9606 GN=EWSR1 PE=1 SV=1 | 2 | 1 | 2 | 1 | 5,3 | 1 |
| P57088 | Transmembrane protein 33 OS=Homo sapiens OX=9606 GN=TMEM33 PE=1 SV=2 | 9 | 2 | 3 | 2 | 6,86 | 2 |
| Q96PK6 | RNA-binding protein 14 OS=Homo sapiens OX=9606 GN=RBM14 PE=1 SV=2 | 3 | 2 | 3 | 2 | 5,99 | 2 |

| Q1KMD3 | Heterogeneous nuclear ribonucleoprotein U-like protein 2 OS=Homo sapiens OX=9606 GN=HNRNPUL2 PE=1 SV=1 | 2 | 1 | 2 | 1 | 4,8 | 1 |
| --- | --- | --- | --- | --- | --- | --- | --- |
| P08134 | Rho-related GTP-binding protein RhoC OS=Homo sapiens OX=9606 GN=RHOC PE=1 SV=1 | 10 | 2 | 4 | 2 | 8,04 | 2 |
| O15118 | NPC intracellular cholesterol transporter 1 OS=Homo sapiens OX=9606 GN=NPC1 PE=1 SV=2 | 1 | 1 | 1 | 1 | 2,76 | 1 |
| P62070 | Ras-related protein R-Ras2 OS=Homo sapiens OX=9606 GN=RRAS2 PE=1 SV=1 | 6 | 1 | 1 | 1 | 2,73 | 1 |
| P39687 | Acidic leucine-rich nuclear phosphoprotein 32 family member A OS=Homo sapiens OX=9606 GN=ANP32A PE=1 SV=1 | 8 | 2 | 2 | 1 | 2,23 | 2 |
| P61289 | Proteasome activator complex subunit 3 OS=Homo sapiens OX=9606 GN=PSME3 PE=1 SV=1 | 7 | 2 | 2 | 2 | 4,13 | 2 |
| P47914 | 60S ribosomal protein L29 OS=Homo sapiens OX=9606 GN=RPL29 PE=1 SV=2 | 9 | 1 | 2 | 1 | 7,11 | 1 |
| P53985 | Monocarboxylate transporter 1 OS=Homo sapiens OX=9606 GN=SLC16A1 PE=1 SV=3 | 3 | 1 | 1 | 1 | 3,65 | 1 |
| Q9BXJ9 | N-alpha-acetyltransferase 15, NatA auxiliary subunit OS=Homo sapiens OX=9606 GN=NAA15 PE=1 SV=1 | 5 | 3 | 4 | 3 | 1,73 | 3 |
| Q15056 | Eukaryotic translation initiation factor 4H OS=Homo sapiens OX=9606 GN=EIF4H PE=1 SV=5 | 5 | 1 | 1 | 1 | 3,11 | 1 |
| A0AVT1 | Ubiquitin-like modifier-activating enzyme 6 OS=Homo sapiens OX=9606 GN=UBA6 PE=1 SV=1 | 3 | 3 | 3 | 3 | 3,57 | 3 |
| Q15907 | Ras-related protein Rab-11B OS=Homo sapiens OX=9606 GN=RAB11B PE=1 SV=4 | 10 | 2 | 2 | 2 | 4,98 | 2 |
| Q9UN86 | Ras GTPase-activating protein-binding protein 2 OS=Homo sapiens OX=9606 GN=G3BP2 PE=1 SV=2 | 3 | 1 | 1 | 1 | 2,56 | 1 |
| Q06203 | Amidophosphoribosyltransferase OS=Homo sapiens OX=9606 GN=PPAT PE=1 SV=1 | 2 | 1 | 1 | 1 | 2,34 | 1 |
| Q12874 | Splicing factor 3A subunit 3 OS=Homo sapiens OX=9606 GN=SF3A3 PE=1 SV=1 | 2 | 1 | 1 | 1 | 2,24 | 1 |
| Q15020 | Squamous cell carcinoma antigen recognized by T-cells 3 OS=Homo sapiens OX=9606 GN=SART3 PE=1 SV=1 | 2 | 2 | 2 | 2 | 2,27 | 2 |

| P00492 | Hypoxanthine-guanine phosphoribosyltransferase OS=Homo sapiens OX=9606 GN=HPRT1 PE=1 SV=2 | 6 | 1 | 2 | 1 | 1,65 | 1 |
| --- | --- | --- | --- | --- | --- | --- | --- |
| P12270 | Nucleoprotein TPR OS=Homo sapiens OX=9606 GN=TPR PE=1 SV=3 | 1 | 3 | 4 | 2 | 3,6 | 3 |
| Q15542 | Transcription initiation factor TFIID subunit 5 OS=Homo sapiens OX=9606 GN=TAF5 PE=1 SV=3 | 3 | 2 | 3 | 2 | 4,03 | 2 |
| P61254 | 60S ribosomal protein L26 OS=Homo sapiens OX=9606 GN=RPL26 PE=1 SV=1 | 14 | 2 | 3 | 2 | 3,92 | 2 |
| O15294 | UDP-N-acetylglucosamine--peptide N-acetylglucosaminyltransferase 110 kDa subunit OS=Homo sapiens OX=9606 GN=OGT PE=1 SV=3 | 2 | 2 | 2 | 2 | 3,93 | 2 |
| Q02790 | Peptidyl-prolyl cis-trans isomerase FKBP4 OS=Homo sapiens OX=9606 GN=FKBP4 PE=1 SV=3 | 4 | 2 | 3 | 2 | 5,77 | 2 |
| Q93052 | Lipoma-preferred partner OS=Homo sapiens OX=9606 GN=LPP PE=1 SV=1 | 2 | 1 | 1 | 1 | 3,27 | 1 |
| O60306 | RNA helicase aquarius OS=Homo sapiens OX=9606 GN=AQR PE=1 SV=4 | 1 | 1 | 1 | 1 | 2,57 | 1 |
| Q96HC4 | PDZ and LIM domain protein 5 OS=Homo sapiens OX=9606 GN=PDLIM5 PE=1 SV=5 | 3 | 2 | 3 | 2 | 4,94 | 2 |
| Q7Z2W4 | Zinc finger CCCH-type antiviral protein 1 OS=Homo sapiens OX=9606 GN=ZC3HAV1 PE=1 SV=3 | 2 | 1 | 1 | 1 | 3,09 | 1 |
| Q9ULT8 | E3 ubiquitin-protein ligase HECTD1 OS=Homo sapiens OX=9606 GN=HECTD1 PE=1 SV=3 | 1 | 2 | 2 | 2 | 1,76 | 2 |
| Q86V81 | THO complex subunit 4 OS=Homo sapiens OX=9606 GN=ALYREF PE=1 SV=3 | 4 | 1 | 1 | 1 | 2,78 | 1 |
| P43487 | Ran-specific GTPase-activating protein OS=Homo sapiens OX=9606 GN=RANBP1 PE=1 SV=1 | 5 | 1 | 1 | 1 | 2,32 | 1 |
| Q9BQ52 | Zinc phosphodiesterase ELAC protein 2 OS=Homo sapiens OX=9606 GN=ELAC2 PE=1 SV=2 | 2 | 2 | 4 | 2 | 6,32 | 2 |
| P08697 | Alpha-2-antiplasmin OS=Homo sapiens OX=9606 GN=SERPINF2 PE=1 SV=3 | 2 | 1 | 2 | 1 | 4,86 | 1 |
| P48637 | Glutathione synthetase OS=Homo sapiens OX=9606 GN=GSS PE=1 SV=1 | 5 | 2 | 2 | 2 | 3,3 | 2 |
| Q9UBQ5 | Eukaryotic translation initiation factor 3 subunit K OS=Homo sapiens OX=9606 GN=EIF3K PE=1 SV=1 | 5 | 1 | 1 | 1 | 2,27 | 1 |

| P45974 | Ubiquitin carboxyl-terminal hydrolase 5 OS=Homo sapiens OX=9606 GN=USP5 PE=1 SV=2 | 3 | 2 | 3 | 2 | 4,94 | 2 |
| --- | --- | --- | --- | --- | --- | --- | --- |
| P49023 | Paxillin OS=Homo sapiens OX=9606 GN=PXN PE=1 SV=3 | 4 | 2 | 2 | 2 | 2,49 | 2 |
| P48960 | CD97 antigen OS=Homo sapiens OX=9606 GN=CD97 PE=1 SV=4 | 2 | 1 | 1 | 1 | 2,26 | 1 |
| Q09161 | Nuclear cap-binding protein subunit 1 OS=Homo sapiens OX=9606 GN=NCBP1 PE=1 SV=1 | 1 | 1 | 1 | 1 | 2,85 | 1 |
| Q9NR31 | GTP-binding protein SAR1a OS=Homo sapiens OX=9606 GN=SAR1A PE=1 SV=1 | 6 | 1 | 2 | 1 | 4,16 | 1 |
| Q5JWF2 | Guanine nucleotide-binding protein G(s) subunit alpha isoforms XLas OS=Homo sapiens OX=9606 GN=GNAS PE=1 SV=2 | 1 | 1 | 1 | 1 | 2,82 | 1 |
| Q92621 | Nuclear pore complex protein Nup205 OS=Homo sapiens OX=9606 GN=NUP205 PE=1 SV=3 | 1 | 2 | 2 | 2 | 2,05 | 2 |
| O60610 | Protein diaphanous homolog 1 OS=Homo sapiens OX=9606 GN=DIAPH1 PE=1 SV=2 | 1 | 2 | 2 | 2 | 1,61 | 2 |
| Q9P258 | Protein RCC2 OS=Homo sapiens OX=9606 GN=RCC2 PE=1 SV=2 | 5 | 2 | 3 | 2 | 7,59 | 2 |
| Q9BR76 | Coronin-1B OS=Homo sapiens OX=9606 GN=CORO1B PE=1 SV=1 | 2 | 1 | 2 | 1 | 4,63 | 1 |
| P62195 | 26S proteasome regulatory subunit 8 OS=Homo sapiens OX=9606  GN=PSMC5 PE=1 SV=1 | 6 | 2 | 3 | 1 | 4,26 | 2 |
| P40261 | Nicotinamide N-methyltransferase OS=Homo sapiens OX=9606 GN=NNMT PE=1 SV=1 | 4 | 1 | 2 | 1 | 5,89 | 1 |
| Q13510 | Acid ceramidase OS=Homo sapiens OX=9606 GN=ASAH1 PE=1 SV=5 | 3 | 1 | 1 | 1 | 2,05 | 1 |
| Q6P179 | Endoplasmic reticulum aminopeptidase 2 OS=Homo sapiens OX=9606 GN=ERAP2 PE=1 SV=2 | 1 | 1 | 2 | 1 | 5,63 | 1 |
| O60884 | DnaJ homolog subfamily A member 2 OS=Homo sapiens OX=9606 GN=DNAJA2 PE=1 SV=1 | 2 | 1 | 2 | 1 | 3,38 | 1 |
| P07741 | Adenine phosphoribosyltransferase OS=Homo sapiens OX=9606 GN=APRT PE=1 SV=2 | 6 | 1 | 1 | 1 | 2,31 | 1 |
| P31431 | Syndecan-4 OS=Homo sapiens OX=9606 GN=SDC4 PE=1 SV=2 | 7 | 1 | 1 | 1 | 2,87 | 1 |
| Q13451 | Peptidyl-prolyl cis-trans isomerase FKBP5 OS=Homo sapiens OX=9606 GN=FKBP5 PE=1 SV=2 | 2 | 1 | 2 | 1 | 3,78 | 1 |
| P11166 | Solute carrier family 2, facilitated glucose transporter member 1 OS=Homo sapiens OX=9606 GN=SLC2A1 PE=1 SV=2 | 2 | 1 | 2 | 1 | 4,39 | 1 |

| P06493 | Cyclin-dependent kinase 1 OS=Homo sapiens OX=9606 GN=CDK1 PE=1 SV=3 | 5 | 1 | 1 | 1 | 2,5 | 1 |
| --- | --- | --- | --- | --- | --- | --- | --- |
| P25786 | Proteasome subunit alpha type-1 OS=Homo sapiens OX=9606 GN=PSMA1 PE=1 SV=1 | 6 | 2 | 2 | 2 | 3,42 | 2 |
| Q00653 | Nuclear factor NF-kappa-B p100 subunit OS=Homo sapiens OX=9606 GN=NFKB2 PE=1 SV=4 | 1 | 1 | 1 | 1 | 2,59 | 1 |
| P52888 | Thimet oligopeptidase OS=Homo sapiens OX=9606 GN=THOP1 PE=1 SV=2 | 1 | 1 | 1 | 1 | 2,08 | 1 |
| Q9UBB4 | Ataxin-10 OS=Homo sapiens OX=9606 GN=ATXN10 PE=1 SV=1 | 2 | 1 | 1 | 1 | 2,73 | 1 |
| P23588 | Eukaryotic translation initiation factor 4B OS=Homo sapiens OX=9606 GN=EIF4B PE=1 SV=2 | 3 | 1 | 1 | 1 | 2,71 | 1 |
| Q9P2E9 | Ribosome-binding protein 1 OS=Homo sapiens OX=9606 GN=RRBP1 PE=1 SV=5 | 1 | 1 | 2 | 1 | 5,12 | 1 |
| Q9H307 | Pinin OS=Homo sapiens OX=9606 GN=PNN PE=1 SV=5 | 2 | 1 | 1 | 1 | 2,84 | 1 |
| O43847 | Nardilysin OS=Homo sapiens OX=9606 GN=NRDC PE=1 SV=2 | 2 | 2 | 3 | 2 | 6,26 | 2 |
| P17812 | CTP synthase 1 OS=Homo sapiens OX=9606 GN=CTPS1 PE=1 SV=2 | 2 | 1 | 1 | 1 | 2,06 | 1 |
| Q9Y6E2 | Basic leucine zipper and W2 domain-containing protein 2 OS=Homo sapiens OX=9606 GN=BZW2 PE=1 SV=1 | 3 | 2 | 4 | 1 | 1,81 | 2 |
| Q9UBF2 | Coatomer subunit gamma-2 OS=Homo sapiens OX=9606 GN=COPG2 PE=1 SV=1 | 1 | 1 | 1 | 1 | 2,27 | 1 |
| P61224 | Ras-related protein Rap-1b OS=Homo sapiens OX=9606 GN=RAP1B PE=1 SV=1 | 6 | 1 | 1 | 1 | 2,51 | 1 |
| P28331 | NADH-ubiquinone oxidoreductase 75 kDa subunit, mitochondrial OS=Homo sapiens OX=9606 GN=NDUFS1 PE=1 SV=3 | 1 | 1 | 2 | 1 | 3,87 | 1 |
| P14174 | Macrophage migration inhibitory factor OS=Homo sapiens OX=9606 GN=MIF PE=1 SV=4 | 8 | 1 | 2 | 1 | 4,66 | 1 |
| Q9BR77 | Coiled-coil domain-containing protein 77 OS=Homo sapiens OX=9606 GN=CCDC77 PE=1 SV=1 | 2 | 1 | 1 | 1 | 2,27 | 1 |
| Q01650 | Large neutral amino acids transporter small subunit 1 OS=Homo sapiens OX=9606 GN=SLC7A5 PE=1 SV=2 | 4 | 1 | 1 | 1 | 2,38 | 1 |
| Q9NQW6 | Anillin OS=Homo sapiens OX=9606 GN=ANLN PE=1 SV=2 | 1 | 1 | 2 | 1 | 3,8 | 1 |
| P24752 | Acetyl-CoA acetyltransferase, mitochondrial OS=Homo sapiens OX=9606 GN=ACAT1 PE=1 SV=1 | 5 | 2 | 2 | 2 | 4,02 | 2 |

| P32322 | Pyrroline-5-carboxylate reductase 1, mitochondrial OS=Homo sapiens OX=9606 GN=PYCR1 PE=1 SV=2 | 8 | 2 | 2 | 2 | 2,42 | 2 |
| --- | --- | --- | --- | --- | --- | --- | --- |
| Q9BUJ2 | Heterogeneous nuclear ribonucleoprotein U-like protein 1 OS=Homo sapiens OX=9606 GN=HNRNPUL1 PE=1 SV=2 | 1 | 1 | 2 | 1 | 4,45 | 1 |
| Q9H5V8 | CUB domain-containing protein 1 OS=Homo sapiens OX=9606 GN=CDCP1 PE=1 SV=3 | 1 | 1 | 1 | 1 | 2,37 | 1 |
| O00203 | AP-3 complex subunit beta-1 OS=Homo sapiens OX=9606 GN=AP3B1 PE=1 SV=3 | 1 | 1 | 1 | 1 | 2,11 | 1 |
| Q9UBM7 | 7-dehydrocholesterol reductase OS=Homo sapiens OX=9606  GN=DHCR7 PE=1 SV=1 | 2 | 1 | 1 | 1 | 2,61 | 1 |
| P17676 | CCAAT/enhancer-binding protein beta OS=Homo sapiens OX=9606 GN=CEBPB PE=1 SV=2 | 3 | 1 | 2 | 1 | 4,51 | 1 |
| P51665 | 26S proteasome non-ATPase regulatory subunit 7 OS=Homo sapiens  OX=9606 GN=PSMD7 PE=1 SV=2 | 4 | 1 | 1 | 1 | 2,27 | 1 |
| P39019 | 40S ribosomal protein S19 OS=Homo sapiens OX=9606 GN=RPS19 PE=1 SV=2 | 6 | 1 | 2 | 1 | 4,77 | 1 |
| Q12765 | Secernin-1 OS=Homo sapiens OX=9606 GN=SCRN1 PE=1 SV=2 | 2 | 1 | 2 | 1 | 2 | 1 |
| Q96K62 | Zinc finger and BTB domain-containing protein 45 OS=Homo sapiens OX=9606 GN=ZBTB45 PE=2 SV=1 | 2 | 1 | 2 | 1 | 5,11 | 1 |
| O43760 | Synaptogyrin-2 OS=Homo sapiens OX=9606 GN=SYNGR2 PE=1 SV=1 | 4 | 1 | 1 | 1 | 2,44 | 1 |
| P24821 | Tenascin OS=Homo sapiens OX=9606 GN=TNC PE=1 SV=3 | 0 | 1 | 1 | 1 | 1,93 | 1 |
| P62081 | 40S ribosomal protein S7 OS=Homo sapiens OX=9606 GN=RPS7 PE=1 SV=1 | 4 | 1 | 2 | 1 | 4,63 | 1 |
| O15347 | High mobility group protein B3 OS=Homo sapiens OX=9606 GN=HMGB3 PE=1 SV=4 | 7 | 1 | 1 | 1 | 2,21 | 1 |
| P26358 | DNA (cytosine-5)-methyltransferase 1 OS=Homo sapiens OX=9606 GN=DNMT1 PE=1 SV=2 | 1 | 2 | 3 | 2 | 2,43 | 2 |
| Q16851 | UTP--glucose-1-phosphate uridylyltransferase OS=Homo sapiens OX=9606 GN=UGP2 PE=1 SV=5 | 2 | 1 | 2 | 1 | 4,08 | 1 |
| Q15075 | Early endosome antigen 1 OS=Homo sapiens OX=9606 GN=EEA1 PE=1 SV=2 | 1 | 1 | 1 | 1 | 2,07 | 1 |
| Q86XP3 | ATP-dependent RNA helicase DDX42 OS=Homo sapiens OX=9606 GN=DDX42 PE=1 SV=1 | 1 | 1 | 1 | 1 | 2,02 | 1 |

| Q9BZZ5 | Apoptosis inhibitor 5 OS=Homo sapiens OX=9606 GN=API5 PE=1 SV=3 | 2 | 1 | 2 | 1 | 3,86 | 1 |
| --- | --- | --- | --- | --- | --- | --- | --- |
| Q9BTE3 | Mini-chromosome maintenance complex-binding protein OS=Homo sapiens OX=9606 GN=MCMBP PE=1 SV=2 | 2 | 1 | 2 | 1 | 2,2 | 1 |
| P28340 | DNA polymerase delta catalytic subunit OS=Homo sapiens OX=9606 GN=POLD1 PE=1 SV=2 | 1 | 2 | 2 | 1 | 1,74 | 2 |
| Q8TCT9 | Minor histocompatibility antigen H13 OS=Homo sapiens OX=9606 GN=HM13 PE=1 SV=1 | 3 | 1 | 1 | 1 | 2,44 | 1 |
| Q8TAT6 | Nuclear protein localization protein 4 homolog OS=Homo sapiens OX=9606 GN=NPLOC4 PE=1 SV=3 | 2 | 1 | 1 | 1 | 2,35 | 1 |
| Q8TEX9 | Importin-4 OS=Homo sapiens OX=9606 GN=IPO4 PE=1 SV=2 | 1 | 1 | 2 | 1 | 4,69 | 1 |
| Q9UGP8 | Translocation protein SEC63 homolog OS=Homo sapiens OX=9606 GN=SEC63 PE=1 SV=2 | 3 | 2 | 2 | 2 | 1,72 | 2 |
| P78344 | Eukaryotic translation initiation factor 4 gamma 2 OS=Homo sapiens OX=9606 GN=EIF4G2 PE=1 SV=1 | 1 | 1 | 2 | 1 | 3,59 | 1 |
| Q96HS1 | Serine/threonine-protein phosphatase PGAM5, mitochondrial OS=Homo sapiens OX=9606 GN=PGAM5 PE=1 SV=2 | 3 | 1 | 1 | 1 | 2,17 | 1 |
| P62244 | 40S ribosomal protein S15a OS=Homo sapiens OX=9606 GN=RPS15A PE=1 SV=2 | 10 | 2 | 3 | 2 | 1,71 | 2 |
| P06703 | Protein S100-A6 OS=Homo sapiens OX=9606 GN=S100A6 PE=1 SV=1 | 9 | 1 | 2 | 1 | 4,73 | 1 |
| Q9ULE4 | Protein FAM184B OS=Homo sapiens OX=9606 GN=FAM184B PE=2 SV=3 | 1 | 1 | 2 | 1 | 5,26 | 1 |
| Q9UNM6 | 26S proteasome non-ATPase regulatory subunit 13 OS=Homo sapiens OX=9606 GN=PSMD13 PE=1 SV=2 | 2 | 1 | 1 | 1 | 1,8 | 1 |
| Q8N1G4 | Leucine-rich repeat-containing protein 47 OS=Homo sapiens OX=9606 GN=LRRC47 PE=1 SV=1 | 2 | 1 | 1 | 1 | 2,16 | 1 |
| P24932 | Shutoff protein OS=Human adenovirus C serotype 2 OX=10515 GN=L4 PE=1 SV=1 | 2 | 2 | 2 | 1 | 1,83 | 2 |
| O94906 | Pre-mRNA-processing factor 6 OS=Homo sapiens OX=9606 GN=PRPF6 PE=1 SV=1 | 1 | 1 | 1 | 1 | 1,66 | 1 |
| O96019 | Actin-like protein 6A OS=Homo sapiens OX=9606 GN=ACTL6A PE=1 SV=1 | 3 | 1 | 1 | 1 | 2,12 | 1 |
| P62829 | 60S ribosomal protein L23 OS=Homo sapiens OX=9606 GN=RPL23 PE=1 SV=1 | 11 | 1 | 1 | 1 | 2,2 | 1 |
| Q5SRE5 | Nucleoporin NUP188 homolog OS=Homo sapiens OX=9606 GN=NUP188 PE=1 SV=1 | 1 | 1 | 1 | 1 | 2,39 | 1 |
| Q9UBL6 | Copine-7 OS=Homo sapiens OX=9606 GN=CPNE7 PE=1 SV=1 | 1 | 1 | 2 | 1 | 1,94 | 1 |
| O43172 | U4/U6 small nuclear ribonucleoprotein Prp4 OS=Homo sapiens OX=9606 GN=PRPF4 PE=1 SV=2 | 3 | 1 | 1 | 1 | 2,37 | 1 |
| P19525 | Interferon-induced, double-stranded RNA-activated protein kinase OS=Homo sapiens OX=9606 GN=EIF2AK2 PE=1 SV=2 | 2 | 1 | 1 | 1 | 2,07 | 1 |
| Q15942 | Zyxin OS=Homo sapiens OX=9606 GN=ZYX PE=1 SV=1 | 3 | 1 | 1 | 1 | 1,86 | 1 |
| O94979 | Protein transport protein Sec31A OS=Homo sapiens OX=9606 GN=SEC31A PE=1 SV=3 | 1 | 1 | 1 | 1 | 2,36 | 1 |
| Q14847 | LIM and SH3 domain protein 1 OS=Homo sapiens OX=9606 GN=LASP1 PE=1 SV=2 | 5 | 1 | 1 | 1 | 2,16 | 1 |
| P53999 | Activated RNA polymerase II transcriptional coactivator p15 OS=Homo sapiens OX=9606 GN=SUB1 PE=1 SV=3 | 10 | 1 | 1 | 1 | 2,27 | 1 |
