# Supplementary material for: UBTD1 regulates ceramide balance and endolysosomal positioning to coordinate EGFR signaling
Source: eLife. 2021 Apr 22;10:e68348. doi: 10.7554/eLife.68348 (PMC8118655; doi:10.7554/eLife.68348)
Supplement: Supplementary file 2. [file elife-68348-supp2.docx]

**Figure 2- Supplementary file 2: Subcellular localization score of UBTD1 interactants in DU145 cell line.**

| **display name** | **canonical name** | **cytoskel eton** | **cytos**  **ol** | **endoplasmic reticulum** | **endos ome** | **extracel lular** | **golgi apparatus** | **lysoso me** | **mitochon drion** | **nucle us** | **peroxis ome** | **plasma membrane** |
| --- | --- | --- | --- | --- | --- | --- | --- | --- | --- | --- | --- | --- |
| **GCN1L1** | Q9UE60 | 1.21509  1 | 4.840  246 | 1.340715 | 0.5143  48 | 0.57101  6 | 0.564747 |  | 1.159666 | 1.266  87 | 0.79312  7 | 2.871718 |
| **AHSA1** | O95433 | 1.53394  1 | 4.796  758 | 3.788184 | 1.0866  02 | 4.31549  9 | 1.178179 | 1.415  888 | 1.657786 | 2.594  967 |  | 1.800087 |
| **RUVBL1** | Q9Y265 | 5.0 | 4.688  446 | 1.106869 | 0.8836  05 | 4.25956  7 | 2.1319 | 1.007  378 | 1.640725 | 5.0 |  | 1.306787 |
| **EIF3G** | O75821 | 1.20611  3 | 4.627  732 | 1.012427 |  | 0.79149  5 |  |  | 1.027945 | 3.425 |  | 0.706857 |
| **ATXN10** | Q9UBB4 | 1.46516  8 | 4.600  19 | 0.948075 | 0.7096  95 | 4.20476  9 | 0.930428 | 0.739  724 | 2.246374 | 2.173  482 |  | 4.48727 |
| **PXN** | P49023 | 5.0 | 4.847  983 | 1.920373 | 1.9830  41 | 2.88394  7 | 1.702686 | 1.783  985 | 1.868799 | 4.240  972 | 0.77956  3 | 5.0 |
| **TAF4** | O00268 | 1.24157  3 | 4.657  276 | 0.961179 |  | 1.19050  5 |  | 0.727  601 | 1.614828 | 5.0 | 0.61547  1 | 1.057419 |
| **CNOT1** | A5YKK6 | 2.05535  9 | 4.522  391 | 2.057374 | 1.5093  67 | 4.40077  4 | 1.348747 | 1.416  246 | 2.229009 | 4.359  076 | 4.31794  1 | 1.988091 |
| **CEBPB** | P17676 | 2.32164  4 | 3.149  692 | 2.355249 | 1.6115  7 | 2.71072  7 | 1.496301 | 1.950  805 | 2.490564 | 5.0 | 2.23044 | 2.392295 |
| **TNPO1** | Q92973 | 1.72585  4 | 4.809  551 | 1.192125 | 0.9846  95 | 4.23744  1 | 1.060806 | 0.785  969 | 1.532504 | 5.0 | 0.50004  2 | 1.421953 |
| **SSB** | P05455 | 1.33292  2 | 2.737  882 | 1.263703 | 1.3335  12 | 1.82575  4 | 0.704912 | 0.770  528 | 1.23202 | 4.843  777 |  | 1.462408 |
| **ARHGEF2** | Q92974 | 5.0 | 4.669  358 | 0.902016 | 1.3908  73 | 1.69436  8 | 3.460739 | 0.946  923 | 1.098076 | 3.866  915 |  | 5.0 |
| **ARHGDIA** | P52565 | 5.0 | 4.704  143 | 1.74858 | 1.7259  01 | 4.40886  6 | 1.510971 | 1.499  573 | 2.192512 | 3.149  299 | 0.57448  8 | 2.455299 |

| **LETM1** | O95202 | 1.05277  2 | 2.478  619 | 1.877258 | 0.7972  84 | 1.14933 | 0.795467 | 0.868  224 | 5.0 | 1.723  088 | 0.70537  3 | 1.707579 |
| --- | --- | --- | --- | --- | --- | --- | --- | --- | --- | --- | --- | --- |
| **VDAC3** | Q9Y277 | 1.83100  3 | 3.429  522 | 2.011313 | 1.1045  49 | 4.31930  7 | 1.1882 | 1.495  076 | 5.0 | 4.346  238 | 1.39932  3 | 1.962565 |
| **NDUFS1** | P28331 | 1.47432  9 | 2.557  521 | 1.200576 |  | 1.28687 |  | 0.966  259 | 5.0 | 1.841  555 | 0.81222  7 | 1.396299 |
| **FXR1** | P51114 | 1.75193  8 | 4.865  988 | 1.314984 | 0.7061  01 | 1.38880  6 | 1.265978 | 0.694  45 | 1.847423 | 4.590  553 |  | 1.440418 |
| **FXR2** | P51116 | 1.50891  6 | 4.690  93 | 0.849419 |  | 1.01125  4 |  |  | 0.931144 | 4.246  023 |  | 1.225587 |
| **RPS15A** | P62244 | 1.73351 | 5.0 | 1.324716 | 1.3664  02 | 4.34934  5 | 1.083321 | 1.310  609 | 1.704631 | 4.408  945 | 0.53517  3 | 1.642984 |
| **MIF** | P14174 | 2.16097  5 | 4.813  884 | 1.887971 | 1.7735  25 | 5.0 | 1.438595 | 1.827  661 | 2.159752 | 4.651  933 | 1.44343  9 | 4.46142 |
| **TOR1AIP1** | Q5JTV8 | 2.45275  9 | 1.5 | 1.450253 |  | 0.56828  6 |  |  | 0.581808 | 5.0 |  | 0.503319 |
| **DDX17** | Q92841 | 1.67342  1 | 3.998  248 | 1.280733 | 1.2410  88 | 1.83286  6 | 0.956845 | 1.051  126 | 1.398781 | 5.0 |  | 1.61063 |
| **RPA1** | P27694 | 1.68028  6 | 2.614  403 | 0.714878 |  | 1.43910  9 | 0.615655 | 0.602  929 | 1.857689 | 5.0 |  | 1.483385 |
| **RPS12** | P25398 | 1.16999  3 | 5.0 | 0.891864 |  | 1.72592  8 | 4.0 |  | 2.374403 | 4.258  529 |  | 0.852723 |
| **APEX1** | P27695 | 4.60684  2 | 3.228  833 | 5.0 | 1.5916  8 | 2.22821  3 | 1.58509 | 1.666  506 | 5.0 | 5.0 | 2.12045 | 2.361982 |
| **CCDC77** | Q9BR77 | 5.0 | 0.937  5 |  |  |  |  |  | 1.21875 | 3.360  692 |  |  |
| **BCLAF1** | Q9NYF8 | 1.58351 | 2.536  698 | 1.247691 | 0.9357  35 | 1.56503  6 | 0.920485 | 0.994  242 | 1.50569 | 5.0 |  | 1.40992 |
| **ANLN** | Q9NQW6 | 5.0 | 1.316  461 | 1.149984 | 1.4674  98 | 1.68701  5 | 0.988508 | 0.674  243 | 1.134575 | 4.818  78 |  | 2.220536 |
| **CORO1B** | Q9BR76 | 5.0 | 5.0 | 0.779074 | 1.1573  83 | 4.20950  3 | 0.72068 | 0.932  009 | 1.625663 | 1.865  665 |  | 4.67704 |

| **IPO9** | Q96P70 | 1.45886  9 | 4.303  356 |  |  | 1.21714 |  |  | 1.273886 | 4.004  228 |  | 0.867307 |
| --- | --- | --- | --- | --- | --- | --- | --- | --- | --- | --- | --- | --- |
| **ELAC2** | Q9BQ52 | 1.18439  8 | 1.932  814 |  |  | 1.20347  9 |  |  | 5.0 | 5.0 | 0.50260  6 | 1.331661 |
| **AQR** | O60306 | 1.18473  2 | 2.872  651 | 1.054282 | 0.6045  2 | 1.52443  7 |  | 0.881  513 | 1.079435 | 5.0 |  | 1.391933 |
| **SERPINB1** | P30740 | 1.64602  7 | 3.116  773 | 1.420642 | 0.7486  68 | 5.0 | 0.861177 | 1.637  115 | 2.580054 | 2.897  746 | 0.65625 | 1.951211 |
| **UBA6** | Q9H3T7 | 1.16992 | 4.466  951 | 1.27643 | 0.7624  86 | 0.75917  9 | 0.573219 | 1.341  407 | 1.487023 | 4.144  526 | 0.54468  3 | 0.797858 |
| **ETF1** | P62495 | 1.19394  2 | 4.408  919 | 1.324392 | 0.8803  43 | 1.20474  7 | 1.004263 | 0.965  93 | 1.502967 | 2.556  547 | 1.80808  7 | 1.382784 |
| **BZW1** | Q7L1Q6 | 1.08174  5 | 2.370  155 | 1.058955 | 0.6683  86 | 1.09518 | 0.583978 | 0.531  294 | 3.1544 | 1.532  288 |  | 1.035102 |
| **ANP32A** | P39687 | 1.75864  4 | 2.907  049 | 5.0 | 1.2323  71 | 1.44561  3 | 1.054389 | 1.150  555 | 1.595607 | 5.0 |  | 1.518619 |
| **PLIN3** | O60664 | 1.70243  5 | 4.813  473 | 2.086476 | 4.4478  43 | 1.72519  3 | 4.476577 | 2.062  569 | 1.92501 | 2.507  793 | 1.79735  8 | 1.779989 |
| **CPSF7** | Q8N684 | 0.86637  1 | 2.705  241 |  |  |  |  |  | 0.588496 | 5.0 |  |  |
| **PSME3** | P61289 | 1.47125  7 | 4.686  729 | 1.466626 | 0.8549  48 | 1.52632  7 | 0.786232 | 1.359  135 | 2.10389 | 4.719  91 | 0.71497  7 | 1.1951 |
| **PFN2** | P35080 | 5.0 | 3.347  903 | 1.229674 | 1.1670  17 | 4.51661  2 | 0.932085 | 0.764  917 | 1.376878 | 1.828  949 | 0.55689  1 | 1.623235 |
| **DNMT1** | P26358 | 2.38922  6 | 2.716  019 | 1.848865 | 1.3173  38 | 2.53357  7 | 1.1676 | 1.548  293 | 3.093947 | 4.899  117 | 1.47898  5 | 2.251609 |
| **ALDH18A1** | Q5T567 | 1.39526  3 | 3.997  889 | 1.42207 | 0.9865  72 | 1.63540  9 | 1.352184 | 0.860  988 | 5.0 | 1.845  315 | 1.46138  9 | 1.893035 |
| **SMC2** | O95347 | 1.33493  1 | 4.505  888 |  |  | 4.11843  7 |  |  | 1.857122 | 5.0 | 0.35937  5 | 0.733523 |
| **KHSRP** | Q92945 | 1.79789  5 | 4.478  587 | 1.301104 | 0.8976  43 | 2.27796  8 | 0.817249 | 0.681  207 | 1.408862 | 4.915  13 |  | 1.247022 |

| **SARS** | P49591 | 1.41720  6 | 4.733  514 | 1.313492 |  | 4.28133  8 | 1.283786 | 0.947  897 | 1.819621 | 3.970  884 | 0.79980  7 | 1.417017 |
| --- | --- | --- | --- | --- | --- | --- | --- | --- | --- | --- | --- | --- |
| **CTSB** | P07858 | 2.48445  8 | 2.960  273 | 2.369421 | 4.5762  82 | 4.77908  1 | 2.194314 | 5.0 | 2.661655 | 4.720  031 | 1.49327 | 4.092922 |
| **UBTD1** | Q9HAC8 |  | 1.568  182 |  |  |  |  |  | 0.663037 | 1.687  694 |  | 1.568182 |
| **PSMD1** | Q99460 | 2.57249  6 | 4.629  795 | 1.30737 |  | 4.23568  2 | 0.766967 | 4.206  422 | 1.648957 | 4.631  705 | 0.375 | 1.16397 |
| **CYFIP1** | Q7L576 | 1.97117 | 4.448  696 | 0.752275 | 0.8950  6 | 4.23454  4 | 0.805913 | 0.585  138 | 1.604654 | 2.211  781 | 0.46875 | 1.653584 |
| **CDC5L** | Q99459 | 1.99677  5 | 1.226  968 | 1.129416 | 0.5427  24 | 1.20176  6 | 0.708082 | 0.625  923 | 1.226925 | 5.0 |  | 1.122818 |
| **ITGA2** | P17301 | 2.37423  3 | 1.928  5 | 1.758858 | 1.5432  94 | 2.84883  1 | 1.382631 | 1.549  472 | 1.611963 | 3.323  366 | 0.95589  6 | 5.0 |
| **AKR1B1** | P15121 | 2.12956  6 | 4.864  753 | 2.048199 | 1.1351  22 | 4.49838  7 | 1.283371 | 2.901  939 | 2.543819 | 4.672  603 | 1.75229  7 | 2.340827 |
| **SLC16A3** | O15427 | 3.07382  5 | 2.110  607 | 1.517227 | 1.2077  67 | 2.42947 | 0.889546 | 1.572  83 | 2.603376 | 4.607  247 | 1.25183  8 | 5.0 |
| **APRT** | P07741 | 1.76292  8 | 4.775  939 | 1.449361 | 0.9872  28 | 4.40558  3 | 0.852144 | 1.052  11 | 2.002213 | 4.698  357 | 1.15168 | 1.701326 |
| **ITGA3** | P26006 | 2.19245  8 | 1.420  426 | 1.677701 | 1.3530  3 | 4.61372  1 | 2.517169 | 1.227  806 | 1.331131 | 1.850  571 |  | 5.0 |
| **POLDIP2** | Q9Y2S7 | 1.46261  9 | 1.656  751 | 1.389962 | 0.8868  01 | 1.38511  2 |  | 0.646  89 | 4.344554 | 3.640  811 | 0.52235  8 | 1.692191 |
| **PLOD2** | O00469 | 1.78386  1 | 1.817  405 | 5.0 | 0.5026  99 | 4.56975  3 | 0.81507 | 0.953  991 | 1.975028 | 2.088  053 | 1.01172  5 | 1.631725 |
| **FAM120A** | Q9NZB2 | 0.97563  3 | 4.571  185 | 0.748623 |  | 0.87977 |  |  | 1.012042 | 4.026  387 | 1.28927  6 | 3.317843 |
| **DNM2** | P50570 | 5.0 | 5.0 | 2.046428 | 3.6216  23 | 4.40718  1 | 4.61674 | 2.280  344 | 3.81244 | 3.318  263 | 1.52460  1 | 5.0 |
| **PSMD7** | P51665 | 1.42453  9 | 4.304  928 | 2.23653 | 0.9612  71 | 4.26348  9 | 0.576367 | 1.139  031 | 1.418127 | 4.696  296 | 0.28125 | 1.801707 |

| **SERPINH1** | P50454 | 2.15416  5 | 1.886  92 | 5.0 | 1.3747  31 | 4.26053  8 | 1.894827 | 1.643  268 | 2.152767 | 2.111  754 | 1.57973  1 | 2.327877 |
| --- | --- | --- | --- | --- | --- | --- | --- | --- | --- | --- | --- | --- |
| **RPS19** | P39019 | 1.55831  8 | 5.0 | 1.375284 | 0.6227  28 | 4.35637  9 | 0.61671 | 0.998  072 | 2.11033 | 5.0 |  | 1.542383 |
| **NEMF** | O60524 | 0.92241  4 | 3.260  286 | 0.791049 |  | 0.66250  3 |  |  | 0.906909 | 4.173  291 |  |  |
| **EIF2AK2** | P19525 | 1.78416  2 | 4.694  707 | 2.260054 | 1.5622  31 | 1.76886 | 1.290649 | 1.533  246 | 1.971126 | 5.0 | 0.85981  8 | 1.603396 |
| **DNAJA2** | O60884 | 2.22085 | 5.0 | 1.540465 | 0.5809  01 | 4.29847  4 | 0.824127 | 1.419  823 | 1.799726 | 3.055  84 | 0.68319  3 | 1.256668 |
| **GLG1** | Q92896 | 3.63084  3 | 1.126  602 | 1.565685 | 1.1073  88 | 4.41189  6 | 4.708799 | 1.174  195 | 1.267348 | 1.439  583 | 0.9375 | 4.459433 |
| **GSPT1** | P15170 | 1.35933  5 | 4.776  174 | 1.147713 | 0.5792  36 | 1.31894  9 | 0.548472 | 0.609  266 | 1.17055 | 2.390  879 |  | 0.950869 |
| **RUVBL2** | Q9Y230 | 4.33522  3 | 4.727  527 | 0.989691 | 0.9836  93 | 4.25473  1 | 0.667908 | 0.692  309 | 1.406729 | 5.0 |  | 2.050432 |
| **RAP1B** | Q6LES0 | 2.10702  2 | 5.0 | 1.443951 | 1.3127  21 | 4.38553  6 | 1.204965 | 4.231  078 | 1.780406 | 2.811  897 | 0.28125 | 5.0 |
| **PRMT5** | Q9UKH1 | 1.83578  8 | 5.0 | 1.244535 | 0.6705  42 | 1.89932  7 | 5.0 | 0.950  538 | 1.766597 | 5.0 | 1.29336  3 | 1.672877 |
| **SF3A3** | Q12874 | 0.98281  7 | 1.928  63 | 0.529972 |  | 0.67823 |  |  | 0.836471 | 5.0 | 0.28125 |  |
| **SRPK1** | Q96SB4 | 1.55452  6 | 4.737  298 | 3.341796 | 0.7784  77 | 1.37731  9 |  |  | 1.057186 | 5.0 |  | 4.506289 |
| **HDAC2** | Q92769 | 2.37270  3 | 3.093  234 | 2.635296 | 1.2826 | 2.23641  4 | 1.100366 | 1.564  375 | 3.815919 | 5.0 | 1.31718  5 | 2.093791 |
| **RAB11B** | Q8NI07 | 3.35840  3 | 4.687  999 | 0.815795 | 4.2694  67 | 4.18035  4 | 1.507879 | 0.695  153 | 0.837553 | 0.995  674 |  | 1.169886 |
| **COPB1** | P53618 | 2.68822  2 | 4.691  826 | 4.353219 | 1.3870  59 | 1.24202  7 | 4.629188 | 1.169  992 | 0.910765 | 1.546  777 |  | 4.316295 |
| **DDX21** | Q9NR30 | 1.59944  4 | 4.607  565 | 1.269149 | 1.3774  19 | 1.71299  3 | 0.923474 | 1.062  573 | 3.778566 | 5.0 | 0.57151  2 | 1.558367 |

| **SAR1A** | Q9NR31 | 1.18838  7 | 3.257  285 | 3.913967 | 1.4019  44 | 1.35692  7 | 3.267104 | 0.956  516 | 1.092143 | 2.387  266 |  | 1.436129 |
| --- | --- | --- | --- | --- | --- | --- | --- | --- | --- | --- | --- | --- |
| **PSMD12** | O00232 | 2.93649  1 | 4.725  545 | 1.21395 | 0.5498  49 | 4.18364  8 |  | 1.219  413 | 1.530812 | 4.630  843 | 1.125 | 0.767561 |
| **GFPT1** | Q06210 | 1.95971  5 | 4.421  596 | 1.805585 | 1.0932  43 | 4.41384  4 | 1.435292 | 1.438  096 | 2.229146 | 2.937  122 | 0.98699  2 | 1.923708 |
| **THOP1** | P52888 | 1.17665  9 | 4.803  072 | 1.530439 | 1.1167  37 | 1.69805  1 | 0.858532 | 1.365  026 | 3.562894 | 2.641  711 |  | 1.639408 |
| **RPL27** | P61353 | 1.49650  6 | 4.425  041 | 4.332587 | 0.5883  57 | 4.30063  1 | 0.748902 | 0.771  977 | 2.39398 | 4.430  736 | 0.65625 | 1.346339 |
| **CBR1** | P16152 | 1.50847  5 | 4.649  797 | 1.842172 | 0.6516  23 | 4.30272  9 | 0.690937 | 1.099  372 | 2.086707 | 2.595  692 | 1.20045  6 | 1.550443 |
| **ATP1A1** | Q9UJ21 | 1.71557 | 1.734  62 | 3.686532 | 2.8598  88 | 4.35237  6 | 3.456746 | 1.300  419 | 1.775966 | 2.288  064 | 0.58049  7 | 5.0 |
| **ARHGEF1** | Q92888 | 2.25755  4 | 4.759  597 | 1.538468 | 1.3681  15 | 2.19119  7 | 1.473319 | 1.324  643 | 2.017157 | 3.526  243 | 0.71412  7 | 4.6648 |
| **SOD2** | Q96EE6 | 2.38427  4 | 3.005  358 | 2.281861 | 1.4614  73 | 4.50116  2 | 1.264124 | 2.050  998 | 5.0 | 2.788  516 | 2.21689  8 | 2.470916 |
| **HECTD1** | Q9ULT8 | 0.61467  9 | 2.511  285 | 0.1875 |  | 0.92769  6 |  | 0.955  583 | 0.961614 | 1.424  659 | 0.28125 | 0.609375 |
| **COPA** | P53621 | 2.05224  7 | 4.624  937 | 4.266453 | 0.9201  24 | 5.0 | 5.0 |  | 1.350273 | 2.983  831 |  | 1.043442 |
| **SCRN1** | Q12765 | 1.16925  3 | 3.542  309 | 0.852341 | 0.5877  6 | 0.94622  7 |  |  | 0.822777 | 5.0 |  | 0.881086 |
| **POLD1** | P28340 | 1.40442  6 | 4.658  375 | 0.756215 |  | 1.39541  4 | 0.839842 | 0.730  801 | 1.615266 | 5.0 | 0.67413  4 | 1.261792 |
| **PPAT** | Q06203 | 1.87576  6 | 4.541  052 | 0.510736 |  | 0.91346  2 |  |  | 1.903365 | 1.399  761 | 0.64835  8 | 0.967722 |
| **CUL2** | Q13617 | 1.88986  9 | 4.698  265 | 1.586806 | 1.1669  37 | 1.66712  9 | 0.834052 | 1.365  914 | 1.833142 | 4.735  232 | 0.9375 | 1.579054 |
| **SYNCRIP** | Q96LC2 | 1.66798 | 3.512  525 | 3.502214 | 1.2576  35 | 1.63289  3 | 1.006105 | 0.881  371 | 1.451421 | 5.0 | 0.54708  8 | 1.456347 |

| **VAPA** | J3QKM9 | 3.22784  4 | 3.403  103 | 5.0 | 2.4085  29 | 1.89336  9 | 3.401545 | 4.416  93 | 1.884605 | 3.727  861 | 1.59499  7 | 5.0 |
| --- | --- | --- | --- | --- | --- | --- | --- | --- | --- | --- | --- | --- |
| **NANS** | Q9NR45 | 1.24944  7 | 4.660  525 | 1.256565 |  | 4.36085  5 | 1.451559 | 0.884  743 | 1.050589 | 3.030  387 |  | 1.451802 |
| **PSMD14** | O00487 | 1.71211  8 | 4.783  912 | 1.666736 | 1.5654  76 | 4.26209  5 | 0.994485 | 1.834  667 | 1.941262 | 4.601  046 | 0.71594  1 | 1.421006 |
| **SNX2** | O60749 | 1.59385  7 | 4.067  711 | 1.474686 | 5.0 | 1.07334  4 | 2.369762 | 4.427  684 | 1.168696 | 2.365  771 | 0.84254  1 | 1.783409 |
| **IPO7** | O95373 | 1.53436  2 | 4.761  58 | 1.094935 | 1.0807  39 | 1.38409  5 | 0.710964 | 0.871  122 | 1.801259 | 5.0 | 0.28125 | 1.96191 |
| **SLC16A1** | P53985 | 5.0 | 1.992  622 | 1.469455 | 1.2465  51 | 4.43656  3 | 1.007445 | 1.495  653 | 2.399425 | 1.997  953 | 1.37645  3 | 5.0 |
| **PSMC6** | P62333 | 2.20353 | 4.833  885 | 1.528831 | 1.2906  5 | 4.46073  4 | 0.977385 | 1.413  543 | 1.821507 | 5.0 | 1.91453  8 | 3.046232 |
| **G3BP2** | Q9UN86 | 1.38670  9 | 4.975  565 | 1.183401 | 0.9274  86 | 1.28250  6 | 0.554954 | 0.990  453 | 1.013646 | 1.739  015 |  | 1.060747 |
| **SNRNP70** | Q9UE45 | 1.65221  7 | 2.685  087 | 1.142628 | 1.1773  74 | 1.81371  4 | 1.040972 | 0.980  466 | 1.504977 | 5.0 | 0.53924  3 | 1.530754 |
| **SQSTM1** | Q13501 | 2.68420  5 | 4.739  704 | 4.119259 | 4.0372  54 | 4.45577  6 | 2.013564 | 4.688  929 | 3.871455 | 5.0 | 2.41412  8 | 2.299193 |
| **SEC24C** | P53992 | 1.21958  6 | 5.0 | 4.409911 | 1.4220  3 | 1.13505  3 | 3.19345 | 1.140  128 | 0.838294 | 2.723  901 |  | 1.515321 |
| **ACTBL2** | Q562R1 | 3.61194  8 | 3.127  334 | 0.510055 |  | 4.18184  9 |  |  | 1.032031 | 1.179  414 |  | 0.501358 |
| **NOL6** | Q9H6R4 | 0.79230  4 | 1.848  567 |  |  | 0.51760  5 |  |  | 4.4937 | 4.660  841 | 0.5625 | 0.920861 |
| **NMT1** | P30419 | 3.11686 | 4.846  783 | 1.641768 | 1.2756  91 | 1.57567  6 | 1.460263 | 1.211  933 | 3.37303 | 3.031  326 | 1.09548 | 4.588425 |
| **ENSP000003 20295** | Q13509 | 5.0 | 1.746  068 | 1.460399 | 1.2099  26 | 4.43131  2 | 1.026291 | 1.323  435 | 1.818132 | 4.484  443 | 0.62933  8 | 1.925523 |
| **RAD23B** | P54727 | 1.49504 | 4.707  969 | 1.245748 | 0.6528  08 | 1.13032  9 | 0.64885 | 0.940  247 | 1.366703 | 5.0 |  | 1.08357 |

| **NCEH1** | Q6PIU2 | 0.80306  5 | 2.622  204 | 4.379565 | 0.9797  39 | 2.30663  6 | 2.628865 | 1.591  319 | 1.607839 | 1.278  505 | 1.54073  2 | 1.779483 |
| --- | --- | --- | --- | --- | --- | --- | --- | --- | --- | --- | --- | --- |
| **SUB1** | P53999 | 1.44491  3 | 1.877  63 | 1.098251 | 0.9900  54 | 4.33970  9 | 1.26915 | 0.965  57 | 1.471803 | 5.0 |  | 1.533435 |
| **ACAT2** | Q9BWD1 | 0.86856  6 | 4.574  638 | 1.470793 |  | 4.28275  5 | 0.822557 | 0.626  289 | 3.880548 | 4.458  04 | 2.71072  2 | 1.171606 |
| **SRP72** | O76094 | 1.26607  1 | 4.751  53 | 5.0 |  | 0.81539 | 0.689462 |  | 0.943101 | 3.324  531 |  | 2.309307 |
| **TXNDC5** | Q8NBS9 | 1.11705  1 | 2.862  542 | 5.0 |  | 4.41700  9 | 0.979845 | 4.259  922 | 1.588873 | 1.385  394 | 0.82748 | 1.471812 |
| **RPL26** | P61254 | 1.37379 | 4.676  215 | 1.267232 |  | 4.27058  4 |  | 0.732  829 | 1.898711 | 4.732  252 |  | 1.081251 |
| **LTA4H** | P09960 | 1.55662 | 4.851  964 | 1.517118 | 0.9996  62 | 4.45734  3 | 0.762657 | 1.546  671 | 1.774147 | 4.751  011 | 1.48017  1 | 2.067474 |
| **NUP205** | Q92621 | 1.24821  1 | 2.700  664 | 0.830984 | 0.7890  87 | 0.76351  3 | 0.955099 |  | 0.758197 | 5.0 |  | 3.04409 |
| **EIF3L** | Q9Y262 | 0.92094 | 4.478  077 | 1.006837 |  | 0.69334  9 | 0.968747 |  | 0.976564 | 3.910  031 |  | 0.647927 |
| **ICAM1** | P05362 | 2.98836  2 | 2.938  787 | 2.407199 | 2.4108  44 | 5.0 | 1.848571 | 2.474  575 | 2.701142 | 3.286  72 | 2.01728  5 | 4.892737 |
| **MYO5B** | Q9ULV0 | 4.24666  2 | 3.398  311 | 1.390676 | 2.4504  76 | 4.27771  7 | 1.893703 | 1.763  739 | 1.810915 | 3.330  747 | 1.07688  5 | 2.345223 |
| **EPHA2** | P29317 | 2.43298 | 2.000  162 | 1.97115 | 2.0927  26 | 2.53850  4 | 1.377678 | 1.844  82 | 1.740769 | 2.363  44 | 0.94494  2 | 5.0 |
| **THRAP3** | Q9Y2W1 | 1.20475  3 | 2.172  954 | 0.544953 |  | 4.19087  7 |  |  | 0.943629 | 5.0 |  | 0.745841 |
| **SEPT2** | Q15019 | 5.0 | 3.829  339 | 1.264515 | 1.3392  33 | 4.35585  5 | 1.359584 | 1.012  463 | 1.648765 | 5.0 | 0.87368  9 | 4.393222 |
| **PCMT1** | Q99625 | 1.32826  7 | 4.829  118 | 0.775218 |  | 4.22093  5 |  | 0.687  554 | 1.097866 | 1.258  599 |  | 1.004989 |
| **CEP170** | Q5SW79 | 5.0 | 4.655  355 | 1.261037 | 0.9750  3 | 1.18083  3 | 1.334517 | 0.708  912 | 1.84924 | 3.570  748 | 0.46875 | 1.448259 |

| **CAPNS1** | P04632 | 2.06210  8 | 4.775  811 | 1.927851 | 1.5224  62 | 4.44689  5 | 1.31935 | 1.657  17 | 2.134691 | 3.565  581 | 0.53162  7 | 4.403013 |
| --- | --- | --- | --- | --- | --- | --- | --- | --- | --- | --- | --- | --- |
| **TPD52L2** | A0A087W  YR3 | 1.21501  6 | 3.034  739 | 0.989333 | 0.7117  6 | 1.76909  2 | 0.627351 | 0.661  119 | 1.150049 | 2.274  184 |  | 1.119413 |
| **ALYREF** | Q86V81 | 2.23256  6 | 4.589  757 | 1.863045 | 1.6735  64 | 4.56252  2 | 1.467854 | 1.704  709 | 2.112289 | 5.0 | 1.24820  4 | 2.257479 |
| **SLC7A5** | Q01650 | 1.56753  2 | 4.602  783 | 1.559869 | 1.6636  65 | 4.41621  1 | 1.110406 | 3.801  442 | 1.996371 | 2.809  952 | 1.10564  1 | 5.0 |
| **SLC2A1** | P11166 | 5.0 | 3.651  272 | 2.663644 | 2.0366  57 | 4.57902  6 | 4.367082 | 2.185  621 | 3.055603 | 3.704  274 | 2.00673  9 | 5.0 |
| **EIF3F** | O00303 | 1.64370  7 | 4.428  325 | 1.360193 | 0.8072  22 | 1.38963  8 | 0.739142 | 1.254  707 | 2.554755 | 2.173  37 | 0.28125 | 1.221756 |
| **LAMC1** | P11047 | 1.81195  1 | 1.065  65 | 4.510208 | 0.5846 | 5.0 | 0.735734 | 1.208  074 | 1.052782 | 1.558  916 | 0.5625 | 1.758201 |
| **RPL23A** | P62750 | 1.25244  9 | 4.617  931 | 1.350958 |  | 4.21224  8 |  | 0.613  544 | 1.294672 | 5.0 |  | 1.07173 |
| **MCM3** | P25205 | 4.55726  6 | 1.533  008 | 1.16022 | 0.6294  33 | 1.49559  9 | 0.975215 | 0.720  666 | 1.494145 | 5.0 | 0.61712 | 1.220834 |
| **LAP3** | P28838 | 1.45177  1 | 4.732  929 | 1.261971 | 0.8420  49 | 4.35526  8 | 2.690217 | 1.592  34 | 3.206505 | 4.532  251 | 0.67983  8 | 1.669987 |
| **ARCN1** | P48444 | 1.12428 | 4.478  345 | 4.395533 | 1.3610  85 | 0.98731  7 | 4.608104 | 1.006  791 | 1.963827 | 1.190  331 | 0.65625 | 0.923733 |
| **SART3** | Q15020 | 1.15702  4 | 0.886  84 |  |  | 1.03937  2 |  |  | 0.895693 | 5.0 |  | 0.827449 |
| **YWHAH** | Q04917 | 1.84038  2 | 4.699  625 | 1.419348 | 0.9478  94 | 4.30160  6 | 0.843676 | 0.910  108 | 2.991432 | 2.760  304 | 0.60074  1 | 4.319674 |
| **EFTUD2** | Q15029 | 1.00327  5 | 4.600  629 | 0.525736 |  | 0.83120  8 | 0.610268 |  | 1.36903 | 5.0 |  | 0.569253 |
| **CKAP4** | Q07065 | 5.0 | 4.580  89 | 5.0 | 1.3679  85 | 4.24797  3 | 1.710885 | 4.252  163 | 1.608283 | 4.735  392 | 1.17812  7 | 5.0 |
| **S100A6** | P06703 | 2.25166 | 5.0 | 1.707839 | 1.3145  28 | 4.44842  9 | 1.39129 | 1.311  909 | 2.481732 | 5.0 | 0.66701  1 | 5.0 |

| **ACTL6A** | O96019 | 1.80021 | 3.799  178 | 0.557901 | 0.5905  4 | 1.19949  2 |  |  | 1.161914 | 5.0 | 0.55271  3 | 4.206291 |
| --- | --- | --- | --- | --- | --- | --- | --- | --- | --- | --- | --- | --- |
| **CAV2** | P51636 | 2.07346 | 1.947  833 | 2.007559 | 1.7605  66 | 2.12439  5 | 5.0 | 1.686  908 | 1.720693 | 5.0 | 1.33701  7 | 5.0 |
| **DIAPH1** | O60610 | 4.45746  4 | 4.623  975 | 1.125616 | 1.1820  39 | 1.71387  4 | 1.148368 | 1.030  31 | 1.278893 | 4.599  525 |  | 4.359613 |
| **ERBB2IP** | Q9NW48 | 1.75525  7 | 2.438  4 | 1.194604 | 1.3880  03 | 4.32342  1 | 0.915832 | 1.215  29 | 3.127938 | 5.0 | 1.73959  5 | 5.0 |
| **PRMT1** | Q99873 | 1.80096 | 4.433  961 | 1.365455 | 0.8426  78 | 1.79402  4 | 0.797741 | 1.124  42 | 2.259798 | 5.0 | 0.88742  9 | 1.778104 |
| **TPR** | P12270 | 5.0 | 2.810  54 | 1.569615 | 1.2270  03 | 2.15639  5 | 1.715712 | 1.431  623 | 1.778372 | 5.0 | 0.81248  8 | 1.977672 |
| **RPL30** | P62888 | 1.38754  8 | 5.0 | 1.465718 |  | 4.27308  1 | 0.585986 | 0.567  188 | 2.234846 | 5.0 | 0.62355 | 1.244311 |
| **MKI67** | P46013 | 1.96263  4 | 2.082  264 | 1.148431 | 0.8665  88 | 1.95201  9 | 0.700357 | 0.684  487 | 2.724367 | 5.0 | 0.88408  5 | 1.722797 |
| **RPL15** | P61313 | 1.21578  2 | 4.603  942 | 1.040706 |  | 1.58460  4 |  |  | 1.273652 | 4.586  715 |  | 1.224816 |
| **SF3B3** | Q15393 | 1.14256  4 | 1.051  09 | 0.849846 | 0.7733  13 | 0.95908  8 | 0.714616 |  | 1.027716 | 5.0 |  | 3.133209 |
| **EIF3E** | P60228 | 1.36386  6 | 4.720  021 | 1.278224 | 0.8251  61 | 4.23722  3 | 0.726093 | 0.545  896 | 1.687211 | 5.0 | 0.26036  6 | 1.055583 |
| **PRPF4** | O43172 | 0.68735  3 | 1.406  25 | 0.630951 |  |  |  |  | 1.216593 | 5.0 |  | 0.633985 |
| **FTH1** | P02794 | 3.46426  9 | 4.669  939 | 1.771922 | 1.7995  62 | 4.45581  3 | 1.315753 | 5.0 | 2.241224 | 4.583  835 | 1.68295  7 | 2.057514 |
| **SLC35B2** | Q8TB61 | 1.08842 | 1.596  09 | 3.464394 | 0.9778  41 | 1.49310  6 | 5.0 | 0.720  485 | 1.354492 | 1.722  136 | 0.70819 | 3.628886 |
| **LRRC47** | Q8N1G4 | 0.71560  8 | 3.294  15 | 0.612712 |  |  |  |  |  | 3.411  171 |  |  |
| **ANP32E** | Q9BTT0 | 0.82795  4 | 2.448  695 |  |  | 0.61191  9 |  |  | 0.84375 | 5.0 |  |  |

| **TOP1** | P11387 | 2.47271  6 | 3.236  677 | 1.754726 | 1.5289  86 | 2.30426  7 | 1.576686 | 1.772  689 | 2.531789 | 5.0 | 1.11027  6 | 2.239303 |
| --- | --- | --- | --- | --- | --- | --- | --- | --- | --- | --- | --- | --- |
| **KIF11** | P52732 | 5.0 | 4.911  665 | 1.195031 | 1.2858  53 | 1.61476  6 | 1.287283 | 1.169  712 | 1.531915 | 4.263  099 | 0.62961  8 | 1.710259 |
| **AP3B1** | O00203 | 1.11562  1 | 2.117  938 | 1.049066 | 1.3966  76 | 1.23794  8 | 4.469618 | 4.376  497 | 1.425035 | 2.940  224 |  | 1.829239 |
| **PHB2** | Q99623 | 1.84699  9 | 3.514  288 | 1.695913 | 0.9995  7 | 1.67637  5 | 1.723064 | 1.597  385 | 5.0 | 5.0 | 1.21699  4 | 1.915935 |
| **NFKB2** | Q00653 | 2.10578  1 | 4.799  756 | 1.570268 | 1.3310  27 | 2.22608  4 | 0.956267 | 1.565  255 | 1.92885 | 5.0 | 0.99588  3 | 2.224697 |
| **AKAP12** | Q02952 | 3.83242  1 | 4.778  148 | 1.3835 | 0.6214  72 | 1.74327 | 0.814934 | 0.684  991 | 1.269167 | 3.302  746 |  | 4.716987 |
| **RBM25** | Q9UIE9 | 1.23166  2 | 2.201  867 | 0.932003 | 0.5428  02 | 0.76011  3 |  | 0.586  615 | 1.281485 | 5.0 |  | 0.872754 |
| **KARS** | Q15046 | 3.01531 | 5.0 | 1.501852 | 1.2408  23 | 4.37183  9 | 1.314465 | 1.291  209 | 5.0 | 5.0 | 0.64717  2 | 3.952817 |
| **ERAP2** | Q6P179 | 1.11661  6 | 1.961  041 | 4.490158 | 1.5160  36 | 1.57728  1 | 1.113487 | 1.314  148 | 3.738037 | 1.603  454 | 0.1875 | 1.473222 |
| **PSMA1** | P25786 | 4.52206  5 | 4.487  719 | 1.318956 | 0.9896  36 | 4.38165  1 | 0.801509 | 1.252  105 | 1.55779 | 4.702  963 | 0.83989  5 | 1.462887 |
| **DHX15** | O43143 | 1.16331  8 | 1.458  105 | 0.9156 | 0.7081  12 | 1.17723  2 | 0.623704 |  | 1.3572 | 5.0 |  | 0.956453 |
| **PRPF19** | Q9UMS4 | 3.64319  8 | 2.204  34 | 0.642537 |  | 1.01827  9 |  | 0.695  692 | 0.992889 | 5.0 |  | 1.97753 |
| **NPLOC4** | Q8TAT6 | 1.44260  6 | 4.635  963 | 5.0 | 1.7030  17 | 1.08073  9 | 1.513276 | 1.656  504 | 1.658448 | 4.657  9 | 0.93617  1 | 1.465026 |
| **HADHA** | P40939 | 1.65977  1 | 2.257  841 | 1.765878 | 0.8179  73 | 1.86274  2 | 1.065863 | 1.205  891 | 5.0 | 2.282  181 | 2.16379  5 | 1.903964 |
| **XPO1** | O14980 | 2.41598  1 | 4.811  667 | 1.967963 | 1.8285  73 | 2.05058  7 | 1.692657 | 1.656  672 | 2.276395 | 5.0 | 1.23216  7 | 2.25032 |
| **WARS** | P23381 | 2.47615  8 | 4.753  085 | 1.6828 | 1.0872  44 | 4.40997  5 | 1.06444 | 1.322  763 | 1.977334 | 4.460  272 | 1.17784  1 | 1.977612 |

| **NUP93** | Q8N1F7 | 1.51837 | 2.063  95 | 1.216001 | 0.7837  51 | 1.13072  6 | 1.068014 |  | 1.123907 | 5.0 |  | 0.90019 |
| --- | --- | --- | --- | --- | --- | --- | --- | --- | --- | --- | --- | --- |
| **GOLGA2** | Q08379 | 5.0 | 3.083  586 | 2.653354 | 2.7145  69 | 2.10599  8 | 5.0 | 2.490  496 | 2.056985 | 3.716  634 | 1.22917  7 | 2.403038 |
| **HSD17B4** | P51659 | 1.22700  2 | 4.541  556 | 1.776397 | 0.8123  07 | 1.35753  7 | 0.804068 | 1.131  273 | 2.255536 | 2.008  373 | 5.0 | 1.275286 |
| **PDLIM7** | Q9NR12 | 4.63618  6 | 4.689  046 | 1.216476 | 1.5138  09 | 1.81180  8 | 1.034699 | 1.629  25 | 1.369481 | 4.749  536 |  | 1.71026 |
| **PCNA** | P12004 | 5.0 | 2.893  203 | 0.234375 |  | 4.31328  5 |  |  | 1.009878 | 5.0 |  | 0.772902 |
| **HNRNPDL** | O14979 | 2.09346  4 | 4.440  221 | 1.543385 | 1.2784  74 | 1.84723  6 | 0.972053 | 1.170  423 | 2.059854 | 5.0 | 0.72227  6 | 1.680347 |
| **CTPS1** | P17812 | 2.69597 | 4.688  968 | 1.726509 | 0.8458 | 1.76648  6 | 1.098467 | 0.503  712 | 2.048123 | 2.296  325 | 1.4789 | 2.520081 |
| **TMEM33** | P57088 | 0.73301  7 | 0.812  025 | 5.0 |  | 0.61429  9 |  | 0.647  216 |  | 4.228  866 |  | 3.0 |
| **RPL7A** | P62424 | 1.34807  6 | 4.566  875 | 1.184763 |  | 1.16581  3 |  | 0.728  9 | 1.372286 | 4.582  922 |  | 1.310389 |
| **EIF4H** | Q15056 | 1.26093  8 | 4.758  123 | 1.034002 |  | 1.02128  8 |  | 0.633  329 | 1.118101 | 4.218  997 |  | 0.804046 |
| **UQCRC1** | P31930 | 1.53598  9 | 4.690  978 | 1.464854 |  | 1.28240  4 |  | 0.952  535 | 4.859672 | 1.690  376 | 1.03216  9 | 1.249436 |
| **XPOT** | O43592 | 1.05460  3 | 4.671  215 | 0.756409 |  | 0.88116  6 |  |  | 1.095221 | 5.0 | 0.50099  2 | 1.523724 |
| **SRRM2** | Q9UQ35 | 1.66710  7 | 1.358 | 1.330832 | 1.0835  69 | 1.69937  5 | 1.144508 | 1.000  668 | 1.308433 | 5.0 |  | 1.535978 |
| **HLA-A** | Q9TPR8 | 1.91190  4 | 2.242  929 | 5.0 | 4.3744  82 | 4.61426  2 | 5.0 | 2.178  75 | 1.980153 | 2.273  003 |  | 5.0 |
| **SPECC1L** | Q69YQ0 | 4.25828  8 | 4.0 |  |  | 0.64474  1 |  |  |  | 0.834  253 |  | 0.86222 |
| **CLPX** | O76031 | 1.5958 | 4.689  795 | 1.397168 | 1.0587  98 | 1.88077  1 | 1.356223 | 0.904  208 | 5.0 | 4.576  789 | 1.18253  1 | 1.474876 |

| **SRP68** | Q9UHB9 |  | 4.739  119 | 4.385978 |  | 0.63803  5 | 0.621073 |  | 1.171003 | 5.0 |  | 0.932855 |
| --- | --- | --- | --- | --- | --- | --- | --- | --- | --- | --- | --- | --- |
| **EWSR1** | Q01844 | 2.56381  1 | 2.715  334 | 1.360218 | 1.2525  71 | 2.04027  4 | 1.723545 | 1.281  456 | 1.651827 | 4.915  249 |  | 3.804677 |
| **PPA1** | Q15181 | 1.86138  1 | 4.732  416 | 1.710945 | 1.5597  11 | 4.35637  5 | 1.713287 | 1.468  971 | 2.320521 | 2.606  288 | 1.49641  3 | 2.151864 |
| **ATIC** | P31939 | 1.39706  5 | 4.745  884 | 0.945274 | 0.6607  87 | 4.31931  4 | 1.021767 | 0.896  559 | 2.075028 | 2.562  592 | 0.55918  6 | 4.560133 |
| **ERO1L** | Q96HE7 | 1.48787  4 | 2.093  758 | 5.0 | 0.9696  18 | 3.00248  7 | 1.729807 | 1.492  763 | 2.16399 | 1.905  324 | 1.36242  1 | 1.83103 |
| **PTGES3** | Q15185 | 1.75226 | 4.587  107 | 1.717679 | 0.9709  01 | 2.12258  6 | 1.272265 | 1.173  547 | 1.603319 | 5.0 | 0.72679  6 | 1.691182 |
| **STAT3** | P40763 | 3.17786  7 | 4.820  637 | 2.721474 | 2.4700  29 | 3.50685  4 | 2.548627 | 2.596  942 | 3.867235 | 5.0 | 2.12475 | 4.646251 |
| **RANGAP1** | P46060 | 3.28077  3 | 4.802  856 | 1.356531 | 0.9903  88 | 1.21695  6 | 1.148987 | 0.966  349 | 1.429856 | 4.783  898 | 0.58371 | 1.501435 |
| **RECQL** | P46063 | 1.32542  9 | 2.170  487 |  |  | 0.86858  1 |  |  | 1.42074 | 4.901  3 | 0.1875 | 0.704956 |
| **ST13** | P50502 | 1.76624  6 | 4.890  494 | 1.75546 | 1.4725  91 | 4.32864  2 | 1.074348 | 1.868  686 | 1.77992 | 2.052  465 | 0.77383  2 | 1.730045 |
| **PRKCDBP** | Q969G5 | 1.39666  1 | 3.745  497 | 0.913431 | 0.9604  21 | 2.16568  9 | 0.774317 | 0.563  132 | 1.25124 | 2.632  208 |  | 5.0 |
| **ACO2** | Q99798 | 1.66920  6 | 4.041  839 | 1.742609 | 1.1233  61 | 1.72036  2 | 1.18147 | 1.480  988 | 4.883309 | 1.955  953 | 1.76351  8 | 1.758566 |
| **EEA1** | Q15075 | 2.60969 | 5.0 | 2.475392 | 5.0 | 4.46209  6 | 2.870393 | 3.258  744 | 2.05033 | 4.157  553 | 1.40395  2 | 5.0 |
| **HADHB** | P55084 | 1.40113  9 | 1.711  282 | 5.0 | 1.0305  03 | 1.75215  1 | 1.169076 | 1.162  583 | 5.0 | 3.242  164 | 2.18902  2 | 1.711134 |
| **EPB41L2** | O43491 | 4.38213  3 | 4.569  582 | 1.034085 | 0.6673  6 | 4.21540  1 |  | 0.512  899 |  | 4.662  487 |  | 4.658528 |
| **CALR** | P27797 | 2.58578  4 | 5.0 | 5.0 | 2.3701  27 | 4.54638  7 | 3.639318 | 2.535  317 | 2.637619 | 5.0 | 1.77362  2 | 3.736244 |

| **CNN2** | Q99439 | 5.0 | 3.807  97 | 0.674859 |  | 4.31907  3 |  |  | 0.917465 | 3.106  665 |  | 1.03089 |
| --- | --- | --- | --- | --- | --- | --- | --- | --- | --- | --- | --- | --- |
| **EMC1** | Q8N766 | 2.28882  6 | 1.122  781 | 5.0 | 1.1754  09 | 1.57243  8 | 1.129239 | 0.812  722 | 1.223521 | 1.094  461 | 1.78711  3 | 1.899635 |
| **NASP** | Q9BTW2 | 1.35428  6 | 2.327  73 | 1.785178 |  | 1.28591  2 |  |  | 1.151541 | 5.0 |  | 1.079224 |
| **SEPT9** | Q9UHD8 | 5.0 | 1.879  484 | 1.027287 | 1.3363  48 | 1.65898 | 1.078142 | 0.956  879 | 1.67361 | 3.349  424 |  | 1.709538 |
| **PDLIM5** | Q96HC4 | 4.57284  5 | 4.783  578 | 1.481254 | 1.0529  15 | 1.85301  3 | 2.17118 | 1.087  555 | 1.566573 | 4.101  553 |  | 2.873202 |
| **KDELR1** | P24390 | 1.76088  7 | 2.008  749 | 5.0 | 1.9144  35 | 1.85093  1 | 5.0 | 1.740  375 | 1.883037 | 1.978  596 | 1.71860  6 | 2.906825 |
| **TRAP1** | Q12931 | 1.33000  3 | 2.720  31 | 1.851023 | 0.5623  42 | 1.15027 | 0.512464 | 1.099  789 | 5.0 | 4.486  932 | 0.67905  5 | 1.133752 |
| **FEN1** | P39748 | 1.98145 | 2.771  319 | 1.272842 | 0.8781  67 | 1.7592 | 0.953687 | 0.934  005 | 5.0 | 5.0 | 0.92781  6 | 2.725145 |
| **GLRX3** | O76003 | 1.33782  2 | 4.660  726 | 1.761456 | 0.8231  44 | 1.42546  2 | 1.161371 | 0.689  043 | 2.539981 | 4.352  593 | 1.07699  4 | 2.546893 |
| **HDGF** | P51858 | 1.83989  8 | 2.830  208 | 1.180976 | 0.7573  58 | 3.87986  3 | 0.935415 | 1.073  548 | 1.401424 | 5.0 |  | 1.794039 |
| **PSMC3** | P17980 | 1.34948  4 | 4.779  308 | 1.095536 |  | 4.20175  3 | 0.814808 | 1.053  185 | 1.262764 | 4.642  706 | 1.93844  7 | 0.901535 |
| **CLUH** | O75153 | 0.73307 | 2.056  361 | 0.642504 |  | 0.1875 | 0.1875 |  | 1.773641 | 2.567  859 |  | 0.532309 |
| **PFKL** | P17858 | 1.63727  3 | 5.0 | 1.039475 | 0.5011  45 | 4.32530  9 | 0.99521 | 0.819  572 | 3.03637 | 3.347  951 | 1.38816  4 | 1.542725 |
| **UPF1** | Q92900 | 1.77312  6 | 4.581  762 | 1.534359 | 0.9161  04 | 1.83327  5 | 1.001074 | 0.923  81 | 2.10764 | 5.0 | 0.75602  8 | 1.501438 |
| **MARS** | P56192 | 1.06582  6 | 5.0 | 1.317537 | 0.6667  23 | 4.33154  1 | 0.733256 | 0.750  89 | 2.051174 | 4.325  453 | 1.51219  1 | 1.568724 |
| **API5** | Q9BZZ5 | 1.14925  8 | 2.633  764 | 0.884841 |  | 1.34059  7 | 1.078125 |  | 1.394841 | 5.0 | 1.07812  5 | 1.090552 |

| **MCM6** | Q14566 | 1.78940  6 | 2.540  228 | 0.930442 | 0.5223  61 | 1.46632  9 | 0.682905 |  | 2.659747 | 5.0 | 0.52165  6 | 1.281501 |
| --- | --- | --- | --- | --- | --- | --- | --- | --- | --- | --- | --- | --- |
| **DEK** | P35659 | 1.58278  9 | 2.637  244 | 0.907746 | 0.8502  01 | 1.70193  8 | 0.853168 | 0.641  983 | 1.421682 | 5.0 |  | 1.685807 |
| **KIAA0368** | Q5VYK3 | 5.0 | 2.680  345 | 5.0 | 5.0 | 0.96339  4 |  | 1.002  624 | 1.451161 | 5.0 | 0.50983  9 | 0.823477 |
| **ADAR** | P55265 | 1.89680  7 | 3.012  176 | 1.597786 | 1.6887  87 | 2.04067  1 | 1.200445 | 1.312  228 | 1.924408 | 5.0 | 0.66399  4 | 1.94622 |
| **ARF4** | P18085 | 1.88433  7 | 5.0 | 2.132999 | 2.0177  13 | 4.32204  1 | 3.99655 | 1.420  229 | 1.972089 | 1.535  404 | 0.62366  7 | 5.0 |
| **CEP131** | Q9UPN4 | 5.0 | 4.387  684 |  | 0.5673  98 | 4.15735  6 | 0.617797 | 0.640  479 | 0.63469 | 3.530  801 |  | 0.937952 |
| **MACF1** | Q9UPN3 | 5.0 | 3.133  441 | 1.53671 | 1.0850  91 | 1.63200  1 | 5.0 | 0.719  52 | 1.762342 | 1.952  944 | 0.5625 | 5.0 |
| **RTCB** | Q9Y3I0 | 1.70974  4 | 4.705  146 | 4.350191 |  | 1.39819  9 | 1.038502 | 0.597  189 | 1.157566 | 5.0 | 0.38421  5 | 1.47091 |
| **EIF4G1** | Q6ZN21 | 2.13610  3 | 4.922  778 | 2.028381 | 1.5871  89 | 1.97494  8 | 1.266277 | 1.766  845 | 2.498465 | 4.431  566 | 0.96972  3 | 1.88236 |
| **UGGT1** | Q9NYU2 | 0.92929 | 1.521  952 | 5.0 | 0.9517  68 | 4.22628 | 1.833274 | 1.530  923 | 1.203295 | 1.055  297 | 0.28125 | 2.925659 |
| **PGM1** | Q9NTY4 | 3.64831  8 | 4.282  363 | 1.442943 | 0.8790  41 | 4.34304  2 | 1.269321 | 1.388  534 | 2.398958 | 1.914  574 | 0.67908  2 | 1.603022 |
| **DHX30** | Q7L2E3 | 0.78253  6 | 4.491  743 |  |  | 0.71065  9 |  |  | 5.0 | 3.468  44 |  | 0.519707 |
| **VPS35** | Q9NZK2 | 2.22013  1 | 5.0 | 1.836833 | 5.0 | 4.33585  3 | 2.759843 | 4.532  752 | 2.076755 | 2.466  547 | 1.44529  2 | 2.207531 |
| **TAF5** | Q15542 | 4.41825  1 | 1.640  625 |  |  |  |  |  | 0.792969 | 5.0 |  |  |
| **USP10** | Q14694 | 1.06649  8 | 4.904  351 | 0.953325 | 5.0 | 0.88027  6 |  | 1.294  297 | 0.999245 | 5.0 | 0.59658  3 | 1.070931 |
| **ACAT1** | P24752 | 1.09897  6 | 2.828  739 | 1.414688 |  | 4.31467  1 | 1.080897 | 0.876  577 | 5.0 | 1.460  19 | 1.92924  2 | 1.362983 |

| **PRPF6** | O94906 | 1.06536  5 | 2.320  801 |  |  | 0.89407  1 |  |  | 1.119166 | 5.0 |  | 0.659857 |
| --- | --- | --- | --- | --- | --- | --- | --- | --- | --- | --- | --- | --- |
| **EIF4A3** | P38919 | 1.64307  8 | 4.608  456 | 1.290301 | 0.7528  13 | 1.43432  6 | 1.25285 | 0.643  27 | 1.746323 | 5.0 |  | 1.298339 |
| **SRSF6** | Q13247 | 1.70777  6 | 1.473  52 | 1.049973 | 0.5785  4 | 1.77571  8 |  |  | 1.186863 | 5.0 |  | 1.360312 |
| **PSMD4** | P55036 | 3.68592  2 | 4.869  998 | 2.610957 | 2.5906  34 | 3.05714  1 | 2.324171 | 2.634  756 | 2.904895 | 4.844  752 | 1.87067  7 | 3.018464 |
| **CD97** | P48960 | 1.43778  8 | 2.328  836 | 0.999691 | 1.0039  54 | 4.39968  4 | 0.57694 | 0.837  733 | 0.901642 | 1.417  695 |  | 4.748173 |
| **RPS5** | P46782 | 1.45507  4 | 5.0 | 1.164305 | 0.6379  29 | 4.34461  8 | 0.529656 | 0.696  014 | 1.867869 | 4.410  869 |  | 1.611051 |
| **ANXA3** | P12429 | 2.42138  6 | 4.855  72 | 1.953803 | 2.4051  62 | 4.50583  9 | 1.702674 | 2.016  407 | 1.991668 | 3.079  33 | 1.18684  5 | 5.0 |
| **RPS9** | P46781 | 1.58203  4 | 5.0 | 1.227014 | 0.7054  77 | 4.32359  1 | 0.791954 | 0.816  902 | 1.748254 | 5.0 | 1.05022  7 | 1.368411 |
| **BUB3** | O43684 | 2.65693  2 | 4.521  355 | 0.743274 | 0.5254  26 | 1.19674  9 |  |  | 1.3688 | 4.813  444 |  | 1.156178 |
| **CAND1** | Q9P0H7 | 1.50315  6 | 4.557  867 | 1.162361 | 0.7644  14 | 4.23375  6 | 4.196055 | 0.947  59 | 1.357663 | 5.0 |  | 1.305446 |
| **PYGB** | P11216 | 1.74277  7 | 3.548  967 | 1.428574 |  | 4.32718  5 | 0.716358 | 4.148  595 | 1.714436 | 2.112  616 | 0.83459  2 | 2.221824 |
| **HYOU1** | Q9Y4L1 | 1.59589  7 | 2.019  735 | 5.0 | 1.5335  91 | 4.47633  2 | 2.34152 | 1.647  318 | 1.984197 | 1.819  641 | 1.10717  8 | 1.641425 |
| **UBA2** | Q9UBT2 | 1.53397  3 | 2.089  761 | 1.15037 | 0.7665  79 | 0.96846  3 |  | 0.846  564 | 1.192086 | 5.0 | 0.5625 | 1.868398 |
| **LGALS1** | P09382 | 1.93922  2 | 4.515  037 | 4.315603 | 1.0904  56 | 4.66032  9 | 1.375251 | 1.360  663 | 1.73228 | 4.050  106 | 0.78560  8 | 3.038988 |
| **GART** | P22102 | 1.53462  3 | 4.463  629 | 1.44302 | 0.9399  14 | 4.33963  4 | 0.666791 | 0.658  878 | 3.281145 | 3.147  094 | 0.96874  7 | 1.626599 |
| **CDH3** | P22223 | 2.51302  6 | 2.781  098 | 1.778528 | 1.0997  26 | 2.73363  9 | 1.239642 | 1.494  436 | 1.423646 | 2.230  619 | 0.1875 | 5.0 |

| **PICALM** | Q13492 | 1.72187  5 | 4.659  23 | 1.419261 | 4.4249  75 | 1.89992  2 | 3.648819 | 1.883  008 | 2.08364 | 4.526  439 | 0.62083 | 5.0 |
| --- | --- | --- | --- | --- | --- | --- | --- | --- | --- | --- | --- | --- |
| **EGFR** | Q9H2C9 | 3.35740  1 | 4.117  4 | 4.096149 | 5.0 | 5.0 | 4.025279 | 3.138  368 | 2.935627 | 5.0 | 2.12296  8 | 5.0 |
| **ESYT1** | Q9BSJ8 | 1.49153  7 | 3.538  436 | 5.0 | 1.3642  62 | 1.09938  3 | 1.333292 | 1.119  704 | 2.189365 | 1.920  011 | 1.63815  8 | 3.902833 |
| **RPL5** | P46777 | 1.49804  4 | 4.765  362 | 4.252967 | 0.5129  58 | 4.28407  1 |  | 0.775  515 | 2.303572 | 5.0 |  | 1.226905 |
| **RPS20** | P60866 | 1.52635  5 | 5.0 | 1.321719 |  | 4.27884  9 | 0.666625 | 0.569  996 | 1.684692 | 4.503  007 |  | 2.204219 |
| **TXNRD1** | Q7LA96 | 1.79829  7 | 4.694  39 | 1.989038 | 0.9210  31 | 4.40854  8 | 0.777012 | 1.489  946 | 4.280634 | 5.0 | 1.59372  9 | 2.028872 |
| **ARHGAP5** | Q6DHZ3 | 2.15029  3 | 4.639  507 | 4.0 | 0.7022  36 | 1.45943  5 | 0.782932 | 0.523  938 | 0.685065 | 2.264  807 | 0.28125 | 3.629513 |
| **SEC23A** | Q15436 | 1.49130  8 | 5.0 | 5.0 | 1.0370  65 | 1.61978  7 | 3.270567 | 1.132  117 | 0.723485 | 3.258  77 | 0.45730  4 | 1.324915 |
| **FUS** | P35637 | 2.54076  9 | 3.021  12 | 2.03268 | 1.8522  95 | 2.21638 | 1.743888 | 1.981  474 | 2.410629 | 5.0 | 1.17460  6 | 2.110969 |
| **GNL3** | Q9BVP2 | 2.68444  1 | 2.383  526 | 0.922549 |  | 4.28892  2 |  | 0.686  391 | 1.257563 | 4.895  572 |  | 1.1169 |
| **SCFD1** | Q9Y6A8 | 1.05290  4 | 4.754  713 | 3.863533 | 1.6550  18 | 0.84952  5 | 5.0 | 1.228  237 | 1.205861 | 2.171  705 | 0.5625 | 4.44077 |
| **PSMC2** | P35998 | 1.15971  4 | 4.776  195 | 1.155019 | 0.6169  83 | 4.19151  9 | 0.691076 | 1.060  707 | 1.293798 | 4.517  963 | 1.65220  5 | 0.925227 |
| **PRPS1L1** | P21108 | 1.07884 | 3.215  075 | 0.577418 |  | 1.09048  8 | 0.708812 | 0.630  572 | 1.670925 | 1.273  079 |  | 1.084608 |
| **TJP2** | Q9UDY2 | 2.63157  4 | 4.720  081 | 1.44516 | 1.5376  85 | 2.29921  2 | 1.293096 | 1.524  197 | 1.70615 | 4.620  489 | 0.64861  8 | 4.692847 |
| **GMPS** | P49915 | 1.55862 | 4.794  172 | 1.268273 | 0.9254  15 | 1.99327 | 1.042707 | 1.317  218 | 2.486677 | 3.143  578 | 0.99960  2 | 1.962284 |
| **TMPO** | P42166 | 1.33280  5 | 2.034  991 | 1.060641 |  | 1.29171  4 |  |  | 1.012167 | 5.0 |  | 1.036872 |

| **SKIV2L2** | P42285 | 0.99794  8 | 2.507  153 | 0.661737 |  | 2.34780  2 |  |  | 1.648111 | 5.0 | 0.375 |  |
| --- | --- | --- | --- | --- | --- | --- | --- | --- | --- | --- | --- | --- |
| **SDHA** | P31040 | 2.11691  1 | 2.223  162 | 1.749332 | 1.2260  5 | 2.07785  5 | 1.266133 | 1.584  548 | 5.0 | 4.487  755 | 1.45892  4 | 1.958395 |
| **SET** | Q01105 | 2.09907 | 5.0 | 5.0 | 1.4934  25 | 2.11427  4 | 1.911275 | 1.571  839 | 2.164469 | 5.0 | 1.45769  9 | 2.076436 |
| **NBR1** | Q14596 | 1.96290  3 | 5.0 | 2.158388 | 4.4416  04 | 1.56519  8 | 1.628586 | 4.094  835 | 2.387962 | 4.640  37 | 2.41152  4 | 1.737205 |
| **TRIM28** | Q13263 | 1.68840  3 | 2.258  754 | 1.130601 | 0.9900  83 | 2.02018  6 | 0.592256 | 1.138  591 | 1.521108 | 5.0 | 0.60515  9 | 1.679709 |
| **HNRNPUL2** | Q1KMD3 | 0.84487  1 | 1.546  875 |  |  |  |  |  | 0.617268 | 4.686  483 |  |  |
| **FHOD1** | Q9Y613 | 5.0 | 4.722  847 | 0.755896 | 0.9871  76 | 1.41668  9 | 0.683065 |  |  | 4.294  549 |  | 1.674821 |
| **DLST** | P36957 | 1.09955  9 | 3.424  558 | 1.123514 |  | 0.95299  6 |  |  | 5.0 | 5.0 | 0.66858  9 | 0.976446 |
| **EIF2A** | Q9BY44 | 0.96501  6 | 4.452  165 | 2.145154 |  | 4.22474  6 | 1.021464 | 0.932  882 | 2.747483 | 2.504  657 | 1.58110  3 | 1.036502 |
| **RPL23** | P62829 | 0.73027  4 | 4.746  341 | 1.828215 |  | 4.14241  3 |  |  | 2.033439 | 4.250  424 |  | 1.018974 |
| **PSMD13** | H0YD73 | 1.29510  5 | 4.300  902 | 0.984527 |  | 4.18581  4 |  | 1.273  269 | 1.420876 | 4.654  617 | 0.46875 | 0.723728 |
| **TAGLN2** | P37802 | 2.92132  4 | 4.764  03 | 1.154671 | 0.9685  8 | 4.39698  9 | 0.610382 | 0.979  539 | 1.447411 | 2.406  005 | 0.73661  9 | 1.493487 |
| **MIB1** | Q86YT6 | 5.0 | 4.540  289 |  | 0.7189  94 | 0.74798  7 |  | 0.650  045 | 1.247644 | 3.000  41 |  | 3.853382 |
| **PSAT1** | Q9Y617 | 1.77121  1 | 4.845  275 | 1.614588 | 1.1053  99 | 4.38412  8 | 1.166562 | 1.230  249 | 2.089233 | 3.129  005 | 1.36984  6 | 1.745948 |
| **SHMT2** | P34897 | 4.68693  8 | 2.321  191 | 1.283228 |  | 4.36567  7 | 1.285597 | 1.212  661 | 5.0 | 5.0 | 2.51363  3 | 1.65208 |
| **FKBP4** | Q02790 | 3.86365  5 | 4.889  343 | 1.781357 | 1.2400  01 | 4.35800  6 | 1.216925 | 1.374  619 | 3.772958 | 4.815  766 | 1.94854  3 | 1.835657 |

| **RPL18A** | Q02543 | 1.19340  6 | 5.0 | 1.05027 | 0.7729  79 | 1.47341 | 0.629329 | 0.528  29 | 1.5104 | 3.157  272 |  | 0.98611 |
| --- | --- | --- | --- | --- | --- | --- | --- | --- | --- | --- | --- | --- |
| **NLN** | Q9BYT8 | 1.07377  6 | 3.002  781 | 0.976243 | 0.5316  08 | 4.28966  5 | 0.814929 | 1.094  421 | 4.475871 | 1.820  428 |  | 2.909 |
| **EIF4A2** | Q14240 | 2.92686  5 | 4.789  2 | 1.893255 | 1.2671  1 | 1.81819  5 | 1.03855 | 1.405  924 | 1.892517 | 3.928  267 | 0.83961  1 | 1.689407 |
| **NUP188** | Q5SRE5 | 1.25633  6 | 2.726  951 | 0.913561 |  | 0.52435  2 | 0.970718 |  | 1.231352 | 4.710  614 |  | 1.116609 |
| **MATR3** | P43243 | 1.72538  2 | 1.382  324 | 1.380654 | 0.9778  49 | 1.23703  8 | 0.64122 | 0.872  249 | 1.581433 | 5.0 |  | 1.245178 |
| **CDC37** | Q16543 | 1.45119  8 | 4.747  133 | 1.11122 |  | 4.20972  3 | 0.520203 | 0.785  347 | 1.29263 | 2.607  399 |  | 1.317945 |
| **MAPRE1** | Q15691 | 5.0 | 4.834  103 | 1.285158 | 1.2391  48 | 1.32419 | 3.496014 | 0.841  28 | 1.227559 | 3.252  354 |  | 3.355133 |
| **RANBP1** | P43487 | 3.80659  5 | 4.677  68 | 1.119191 | 1.0278  28 | 1.09336  3 | 1.14954 | 0.631  419 | 1.384183 | 4.456  344 |  | 1.282948 |
| **RRBP1** | Q9P2E9 | 1.47508  8 | 1.918  869 | 4.886035 | 0.6131  55 | 2.22510  5 | 0.866827 | 0.810  815 | 1.594117 | 1.771  945 |  | 1.395688 |
| **STRN3** | Q13033 | 2.34711  9 | 3.382  647 |  |  | 0.61585  4 | 5.0 |  | 0.804869 | 5.0 |  | 5.0 |
| **SF3A1** | Q15459 | 1.19193 | 2.573  209 | 0.699952 | 0.5769  65 | 1.05372  4 | 0.627364 |  | 1.039916 | 5.0 | 1.17187  5 | 1.6956 |
| **GTF2I** | Q75M88 | 1.52971  2 | 2.937  494 | 1.318441 |  | 1.58291  6 |  | 0.557  186 | 0.93417 | 5.0 | 0.1875 | 1.558 |
| **FARSB** | Q9NSD9 | 0.81021  2 | 4.664  159 | 0.631701 |  | 0.63895  6 |  |  | 0.985431 | 2.101  771 |  | 0.529403 |
| **DSG2** | Q14126 | 2.47209  5 | 1.504  56 | 1.663891 | 1.3114  26 | 4.43501  6 | 1.006203 | 1.218  098 | 1.237531 | 1.947  884 |  | 5.0 |
| **RARS** | P54136 | 2.44474  6 | 5.0 | 1.314356 | 0.5154  92 | 4.41915  3 | 0.772033 | 0.876  851 | 2.009897 | 4.807  008 | 2.26683  7 | 1.903214 |
| **EIF4G2** | Q8NI71 | 1.47493  4 | 4.797  61 | 1.440816 | 0.6650  76 | 2.07025  7 |  | 0.930  808 | 2.319627 | 2.714  277 |  | 2.156101 |

| **PDIA4** | P13667 | 1.49682  6 | 1.875  545 | 4.870944 | 1.2072  92 | 5.0 | 1.763496 | 1.480  173 | 1.812295 | 1.706  777 | 1.03633  4 | 1.67284 |
| --- | --- | --- | --- | --- | --- | --- | --- | --- | --- | --- | --- | --- |
| **PIBF1** | Q8WXW3 | 5.0 |  |  |  | 5.0 | 0.620393 |  | 0.46875 | 5.0 |  | 0.848873 |
| **CAD** | P27708 | 1.29281  1 | 4.583  786 | 1.178369 | 0.7977  33 | 4.25396  9 | 0.790429 | 0.870  814 | 2.06987 | 4.616  133 | 1.37694  5 | 2.589036 |
| **G3BP1** | Q13283 | 2.04652  6 | 5.0 | 1.84549 | 1.6202 | 1.68153  7 | 1.356764 | 1.500  677 | 1.714358 | 4.603  436 | 0.97482  8 | 1.658191 |
| **SRRM1** | Q8IYB3 | 1.96538 | 4.284  062 | 1.318396 | 0.8461  64 | 1.63839  7 | 1.312124 | 0.826  511 | 1.238867 | 5.0 |  | 1.704075 |
| **PRDX4** | Q13162 | 1.71703  5 | 4.441  879 | 4.39614 | 1.0212  06 | 4.56580  5 | 1.143832 | 1.327  702 | 2.290859 | 4.370  847 | 2.20148  6 | 2.232333 |
| **EIF4B** | P23588 | 1.76440  4 | 4.846  995 | 1.546272 | 1.1035  86 | 1.62549  9 | 0.789329 | 1.513  07 | 1.692497 | 3.784  422 | 0.75573  7 | 2.809828 |
| **IARS2** | Q9NSE4 | 0.90384  5 | 4.696  823 | 0.882991 |  | 0.94328  2 | 0.599376 |  | 4.855773 | 1.811  834 |  | 0.96538 |
| **MCM2** | P49736 | 4.60900  3 | 2.646  924 | 1.099343 | 0.6746  52 | 1.69929 | 0.926215 | 0.733  748 | 1.608101 | 5.0 | 0.84622  6 | 1.491324 |
| **OGT** | O15294 | 3.01213  2 | 5.0 | 2.261806 | 1.4834  23 | 2.38478  8 | 1.942379 | 1.995  459 | 3.98631 | 5.0 | 1.45122  1 | 5.0 |
| **SYNGR2** | O43760 | 0.90244  3 | 0.828  91 | 1.008362 | 1.4149  41 | 4.20086  7 | 2.352875 | 0.844  856 | 0.674089 | 0.892  423 |  | 3.447152 |
| **FAF2** | Q96CS3 | 1.11125  5 | 2.857  404 | 4.436101 | 0.9708  61 | 4.23511  7 | 1.283232 | 4.245  073 | 1.44019 | 2.867  117 | 1.46753 | 1.132072 |
| **RPN2** | P04844 | 1.04372  1 | 1.136  704 | 4.655154 | 1.0584  85 | 2.38342  1 | 1.430381 | 1.277  205 | 1.049339 | 1.122  487 | 0.1875 | 1.752383 |
| **NCBP1** | Q09161 | 1.65271  2 | 4.715  256 | 1.258126 | 0.7738  33 | 1.71997  4 | 0.645895 | 0.751  59 | 4.520726 | 5.0 | 0.54716  2 | 1.517478 |
| **MYL6B** | P14649 | 4.58242  8 | 4.663  052 | 1.691152 | 0.6725  62 | 4.39173 |  | 0.944  263 | 1.991303 | 2.261  896 |  | 1.850364 |
| **LONP1** | P36776 | 1.43629  4 | 4.684  306 | 1.625665 | 0.6961  51 | 1.36778  8 | 0.825707 | 1.417  257 | 5.0 | 4.428  949 | 2.28874  6 | 1.367347 |

| **LRRFIP1** | Q32MZ4 | 4.2777 | 4.758  03 | 1.363681 | 1.7612  2 | 1.44909  4 | 1.004439 | 1.274  215 | 1.343501 | 5.0 | 0.74057  9 | 4.515626 |
| --- | --- | --- | --- | --- | --- | --- | --- | --- | --- | --- | --- | --- |
| **NUP155** | O75694 | 1.40391  6 | 1.96 | 1.327793 |  | 1.19515  1 | 0.939844 |  | 0.783714 | 5.0 | 0.1875 | 2.23897 |
| **LPP** | Q93052 | 3.77385 | 4.828  573 | 1.3488 | 1.0024  7 | 2.08285  5 | 1.099856 | 0.947  37 | 1.4117 | 5.0 | 1.09850  2 | 5.0 |
| **TAF6** | P49848 | 1.03893  8 | 4.611  88 |  |  |  |  |  | 0.91213 | 5.0 | 0.1875 |  |
| **GSS** | P48637 | 1.24166  9 | 4.471  527 | 1.487779 |  | 4.37864  3 | 0.76221 | 1.020  148 | 2.054818 | 3.286  161 | 1.78081 | 1.691749 |
| **SMARCC2** | Q8TAQ2 | 1.41574  6 | 2.251  962 | 0.740712 |  | 1.01594  4 |  |  | 1.018805 | 5.0 | 0.62426  5 | 1.015384 |
| **HPRT1** | P00492 | 2.62757  3 | 4.836  996 | 2.057679 | 1.7912  97 | 4.56952  3 | 1.592318 | 1.975  148 | 2.675348 | 3.513  154 | 1.70346 | 3.111414 |
| **PFAS** | O15067 | 1.23641  2 | 4.657  101 | 0.9758 |  | 4.31689  8 | 1.302477 | 0.790  533 | 2.567712 | 3.593  137 | 1.57341  5 | 2.472415 |
| **SDPR** | O95810 | 1.72052  6 | 5.0 | 1.725321 | 1.3411  84 | 1.97355  6 | 2.139902 | 1.336  074 | 2.404361 | 4.629  637 | 1.51850  3 | 5.0 |
| **CD2AP** | Q9Y5K6 | 5.0 | 4.806  404 | 2.455223 | 1.9644  34 | 4.56116  5 | 1.634873 | 1.920  329 | 2.50641 | 4.769  519 | 1.01459  6 | 4.762731 |
| **HDLBP** | Q00341 | 1.65068  2 | 4.717  106 | 1.384237 | 0.5294  73 | 3.60662  1 | 0.65903 | 0.916  172 | 1.306612 | 4.085  655 |  | 4.281705 |
| **GAPVD1** | Q96CZ4 | 1.17549  3 | 5.0 |  | 3.7196  12 | 0.78748  7 | 1.127593 | 1.213  927 | 0.705255 | 0.816  281 |  | 4.614155 |
| **SNX6** | Q9UNH7 | 1.62331  1 | 4.005  857 | 1.269336 | 5.0 | 1.15064  4 | 2.21362 | 1.981  235 | 1.149127 | 3.967  219 | 0.76824  5 | 1.642105 |
| **CCT2** | P78371 | 5.0 | 5.0 | 1.348304 | 1.1896  57 | 4.27464  2 | 0.860295 | 4.190  076 | 1.903795 | 1.823  439 |  | 1.422554 |
| **EIF5** | P55010 | 1.39904 | 4.809  843 | 1.509272 | 0.7258  14 | 1.23243  8 | 0.848823 | 0.877  405 | 1.746597 | 2.617  952 |  | 4.516831 |
| **CPNE1** | Q99829 | 1.28948  4 | 5.0 | 0.618114 | 1.1878  52 | 4.24066  8 | 0.801784 | 4.200  131 | 0.937746 | 5.0 |  | 3.611921 |

| **PAICS** | P22234 | 2.04042 | 4.423  189 | 2.033741 | 1.4839  15 | 4.39125 | 1.785178 | 1.442  495 | 2.49389 | 2.559  272 | 1.48831  9 | 2.277334 |
| --- | --- | --- | --- | --- | --- | --- | --- | --- | --- | --- | --- | --- |
| **RBM14** | Q96PK6 | 1.32007  2 | 1.302  563 | 0.66811 | 0.9111  72 | 1.39715  8 | 0.78635 | 0.958  713 | 1.106409 | 5.0 | 0.62744  3 | 1.242168 |
| **BAG3** | O95817 | 3.35934 | 4.852  977 | 1.898515 | 1.5352  05 | 1.89830  5 | 1.323192 | 2.198  768 | 2.068448 | 5.0 | 1.07024  1 | 4.686772 |
| **EIF3A** | Q14152 | 2.89462  2 | 4.603  363 | 0.837102 | 0.5627  5 | 1.61028  6 | 0.549498 | 0.836  221 | 1.094167 | 4.787  694 |  | 2.042615 |
| **QARS** | P47897 | 1.01585  9 | 5.0 | 0.949588 |  | 1.03925  1 | 0.584756 |  | 4.372925 | 1.525  485 | 0.52919  4 | 1.033647 |
| **EIF3K** | Q9UBQ5 | 1.29020  3 | 4.778  153 | 1.364766 |  | 1.27905  8 |  |  | 1.1122 | 3.921  03 | 0.55301  7 | 1.090134 |
| **IRF2BP1** | Q8IU81 |  | 0.166  703 |  |  |  |  | 0.533  221 |  | 5.0 |  |  |
| **UBAP2L** | Q14157 | 1.03970  5 | 3.078  259 | 0.763012 |  | 0.61487  1 |  |  | 0.817882 | 5.0 |  | 0.561261 |
| **CDC42** | P60953 | 5.0 | 4.764  546 | 4.466121 | 2.7282  23 | 4.59117  4 | 4.003702 | 2.395  196 | 2.609221 | 3.440  005 | 1.37444  8 | 5.0 |
| **OTUB1** | Q96FW1 | 1.82075  6 | 4.596  912 | 1.38712 | 1.5002  28 | 4.31920  6 | 0.926058 | 1.451  893 | 2.578059 | 4.688  281 |  | 1.447627 |
| **PPP1CC** | P36873 | 2.27949  5 | 4.727  662 | 1.942543 | 1.2929  28 | 1.95279  4 | 1.137285 | 1.005  443 | 5.0 | 5.0 | 0.55915  3 | 3.738907 |
| **HNRNPL** | P14866 | 1.67843  8 | 3.540  918 | 1.374599 | 0.7842  58 | 4.35141  1 | 0.733625 | 0.939  044 | 1.996749 | 5.0 | 0.55317  1 | 1.633779 |
| **RBBP4** | Q09028 | 1.58569  6 | 4.626  641 | 0.574144 |  | 1.33657  4 |  |  | 1.150675 | 5.0 |  | 1.103913 |
| **TNPO3** | Q9Y5L0 | 1.42440  2 | 2.513  036 | 1.066468 | 1.1478  85 | 1.47795 | 1.003613 | 0.648  107 | 1.324812 | 5.0 | 0.1875 | 1.356457 |
| **FAM184B** | Q9ULE4 |  | 1.468  753 |  |  |  | 0.578125 |  |  | 2.333  046 |  |  |
| **IPO4** | Q8TEX9 | 2.18342 | 3.596  05 | 0.695261 |  | 0.84096  5 |  |  | 1.081258 | 5.0 |  | 0.676739 |

| **AP1G1** | O43747 | 1.33716  2 | 4.721  666 | 1.110384 | 5.0 | 1.47063 | 5.0 | 4.333  683 | 2.787538 | 1.530  312 |  | 1.810704 |
| --- | --- | --- | --- | --- | --- | --- | --- | --- | --- | --- | --- | --- |
| **HM13** | A0A0C4D GU3 | 1.18043  7 | 1.563  143 | 5.0 | 1.5196  07 | 1.65569  2 | 1.366596 | 1.522  29 | 1.607533 | 1.543  705 |  | 4.298971 |
| **KTN1** | Q86UP2 | 2.01658  3 | 1.241  044 | 5.0 | 0.9395  83 | 1.54686  7 | 1.434933 | 0.956  824 | 1.566184 | 1.608  551 | 0.99331 | 4.27223 |
| **DPP3** | Q9NY33 | 1.08282  6 | 4.336  493 | 1.029274 | 0.7488  51 | 4.26139 | 0.712798 | 1.504  722 | 1.243556 | 1.395  903 | 0.73011  1 | 1.115195 |
| **SLC3A2** | P08195 | 1.83485 | 4.636  783 | 2.140655 | 1.7227  6 | 4.49478  3 | 1.264055 | 3.763  02 | 2.17733 | 4.610  428 | 1.57188  6 | 5.0 |
| **UAP1** | Q16222 | 1.12671  2 | 4.759  08 | 1.380663 |  | 1.47397  3 | 1.250216 | 0.851  657 | 1.337145 | 4.565  622 | 1.21875 | 4.496155 |
| **ADK** | P55263 | 1.75314  4 | 4.806  714 | 1.290019 | 0.8230  82 | 2.43073  4 | 0.785948 | 1.147  501 | 2.814698 | 4.689  014 | 2.00898  8 | 2.762005 |
| **SEC31A** | Q6ZU90 | 1.53674  7 | 4.684  731 | 5.0 | 1.4905  1 | 1.27245  1 | 3.310001 | 1.315  159 | 1.214027 | 3.224  081 |  | 1.477772 |
| **IDH2** | P48735 | 2.09201  2 | 3.990  391 | 1.729412 | 1.0599  81 | 4.43488  8 | 1.245724 | 1.360  698 | 5.0 | 2.599  194 | 3.35407  4 | 2.13986 |
| **USP14** | P54578 | 1.74901  4 | 4.787  265 | 1.95202 | 1.6530  19 | 4.29447  9 | 1.09193 | 2.030  246 | 1.694062 | 4.226  482 | 0.68863  5 | 3.680878 |
| **YARS** | P54577 | 1.79745  5 | 4.846  037 | 1.374366 | 0.9763  62 | 4.38446 | 1.007375 | 1.471  169 | 1.983163 | 4.607  789 |  | 1.887294 |
| **ASAH1** | Q13510 | 1.36329  9 | 3.112  093 | 1.932733 | 2.0094  15 | 5.0 | 1.821227 | 5.0 | 2.150987 | 5.0 | 1.62066  1 | 1.989739 |
| **HERC2** | Q86YY8 | 5.0 | 4.475  196 | 0.575811 |  | 0.71617  2 |  | 0.691  518 |  | 5.0 | 0.61201  1 | 4.0 |
| **LASP1** | Q14847 | 4.42964  7 | 3.972  759 | 0.814463 | 0.9082  17 | 1.56531  2 | 0.604462 | 0.595  508 | 1.072289 | 3.630  523 |  | 2.690811 |
| **OFD1** | O75665 | 5.0 | 4.468  072 | 1.00579 | 1.0065  55 | 2.68135  9 | 1.201075 | 1.130  335 | 0.954292 | 4.160  264 |  | 1.57149 |
| **MCM5** | P33992 | 1.81750  8 | 3.632  593 | 1.171045 |  | 1.52991  7 | 0.751245 | 0.754  367 | 1.630689 | 4.845  329 | 0.57372  9 | 1.353564 |

| **MCM7** | P33993 | 2.19674 | 4.542  116 | 1.348246 | 0.8807  9 | 1.90284  7 | 0.956519 | 1.028  181 | 1.799589 | 5.0 | 0.79186  1 | 1.646133 |
| --- | --- | --- | --- | --- | --- | --- | --- | --- | --- | --- | --- | --- |
| **RPS7** | P62081 | 3.56146  4 | 5.0 | 0.921274 |  | 1.23630  7 | 0.570827 |  | 1.619283 | 5.0 |  | 1.091702 |
| **PPP4R1** | Q8TF05 | 1.37850  6 | 2.878  26 |  |  | 0.59388  5 |  |  |  | 3.461  892 |  |  |
| **MCM4** | P33991 | 1.80652  3 | 2.773  606 | 0.963642 |  | 1.43590  1 | 0.665856 | 0.623  049 | 1.429843 | 4.911  639 |  | 1.308585 |
| **STAT1** | P42224 | 2.61741  8 | 4.810  753 | 2.437124 | 2.4708  59 | 3.28405  2 | 2.249912 | 2.384  636 | 2.604809 | 5.0 | 1.82991  8 | 2.870409 |
| **RPL29** | P47914 | 1.48805 | 4.425  804 | 1.17766 | 0.5704  3 | 1.34388  9 | 0.824101 |  | 1.462588 | 2.983  479 |  | 1.094561 |
| **MCMBP** | Q9BTE3 | 1.06212  8 | 4.551  707 |  |  |  |  |  | 1.321449 | 5.0 |  | 1.644135 |
| **IARS** | P41252 | 1.32703  6 | 5.0 | 1.301106 | 0.7467  79 | 4.31406  2 | 0.890424 | 1.164  845 | 1.844375 | 3.069  097 | 0.74411  1 | 1.586855 |
| **PSMC5** | P62195 | 1.13261  4 | 4.806  566 | 1.151827 | 0.8002  48 | 4.15351  1 | 0.596179 | 0.958  917 | 1.194734 | 5.0 | 1.91511  8 | 2.57017 |
| **PPM1G** | O15355 | 1.42013  1 | 1.695  582 | 1.079787 | 0.7231  52 | 1.07516  2 |  | 0.649  047 | 1.20553 | 4.923  4 | 0.28125 | 1.393002 |
| **USP5** | P45974 | 2.65262  1 | 4.676  893 | 1.684294 | 1.3611  91 | 1.45963  9 | 0.901202 | 4.311  795 | 1.460934 | 3.656  002 | 1.24136  9 | 1.707588 |
| **NPC1** | O15118 | 1.77662 | 2.061  277 | 5.0 | 4.2331  08 | 4.45925  9 | 3.27345 | 5.0 | 1.985913 | 5.0 | 1.69643  8 | 5.0 |
| **EIF3D** | O15371 | 1.08940  7 | 4.799  099 | 1.025501 |  | 0.89391  2 |  |  | 1.018581 | 2.059  026 |  | 0.788283 |
| **DNAJA1** | P31689 | 4.57525  8 | 5.0 | 4.387551 | 1.2937  01 | 4.28615  9 | 1.295579 | 1.567  862 | 3.708957 | 4.127  404 | 0.81482  9 | 1.545845 |
| **ASPH** | Q6NXR7 | 1.88708  5 | 2.602  913 | 5.0 | 0.8456  19 | 1.99443  5 | 0.773305 | 0.949  022 | 2.169927 | 2.348  856 | 0.63003  4 | 5.0 |
| **ZYX** | Q15942 | 5.0 | 4.777  086 | 1.486167 | 1.2255  34 | 2.17484  2 | 1.293666 | 0.950  475 | 1.482207 | 5.0 | 0.67953  3 | 2.324471 |

| **SF3B1** | O75533 | 1.20682  3 | 1.178  392 | 0.8584 |  | 1.01575  8 |  |  | 1.134708 | 5.0 |  | 2.978689 |
| --- | --- | --- | --- | --- | --- | --- | --- | --- | --- | --- | --- | --- |
| **ESD** | P10768 | 1.22614  7 | 4.584  958 | 4.213076 |  | 4.32019 | 2.027142 | 0.535  503 | 1.518521 | 3.195  938 | 1.44217  5 | 2.547633 |
| **CSDE1** | Q9Y2S4 | 1.36519  2 | 4.818  322 | 1.152328 | 0.6019  73 | 1.31231 | 4.299891 | 0.649  514 | 1.261634 | 3.490  461 |  | 4.502402 |
| **LBR** | Q14739 | 2.16113  3 | 2.009  644 | 5.0 | 1.4247  41 | 1.93407  1 | 1.583055 | 1.464  251 | 1.774699 | 5.0 | 1.45985  4 | 3.663412 |
| **CDCP1** | Q9H5V8 | 1.41623  2 | 1.263  714 | 1.162066 | 0.8981  05 | 5.0 |  | 0.938  255 | 0.935268 | 1.459  454 | 0.14062  5 | 4.235421 |
| **ZBTB45** | Q96K62 |  | 2.897  779 |  |  |  |  |  |  | 4.228  582 |  |  |
| **NRD1** | O43847 | 1.19249  9 | 5.0 | 1.068753 | 0.9872  44 | 1.41430  6 | 0.672233 | 0.892  428 | 5.0 | 2.660  399 |  | 1.221901 |
| **TUBB6** | Q9BUF5 | 5.0 | 3.251  997 | 1.024821 | 0.6628  4 | 4.32670  9 | 0.79992 | 0.807  196 | 1.212188 | 4.455  581 | 0.5625 | 1.278635 |
| **SSRP1** | Q08945 | 1.30051  2 | 3.057  78 | 0.629565 |  | 1.05672  3 |  |  | 0.998614 | 4.748  57 |  | 0.848876 |
| **PUF60** | Q9UHX1 | 1.02364  7 | 1.546  384 | 0.763188 |  | 0.68416  6 | 1.661078 |  | 1.886436 | 4.870  156 |  |  |
| **KPNA2** | P52292 | 1.83181  4 | 4.806  51 | 1.436209 | 1.1246  67 | 1.49353  7 | 1.303647 | 0.866  999 | 2.044798 | 4.750  441 | 0.53986  5 | 4.374342 |
| **CD44** | Q96J24 | 3.34669  9 | 4.683  148 | 2.545057 | 2.5481  3 | 4.76988  7 | 4.628298 | 2.575  43 | 2.777202 | 3.264  321 | 1.82811  3 | 5.0 |
| **CPNE7** | Q9UBL6 | 0.69865  3 | 2.962  761 | 0.532106 | 0.9857  13 | 4.18217  7 | 0.720832 |  | 0.550351 | 5.0 |  | 5.0 |
| **SERPINF2** | P08697 | 1.89633  7 | 2.209  795 | 1.581192 | 1.2281  36 | 5.0 | 1.291718 | 1.459  523 | 1.766546 | 2.053  323 | 0.98091  5 | 2.207613 |
| **ANP32B** | Q92688 | 0.94137  4 | 2.725  722 | 0.717247 | 0.7037  48 | 4.19614  1 |  |  | 1.593891 | 5.0 |  | 0.928609 |
| **SNRNP200** | O75643 | 1.10844 | 2.263  412 | 0.907283 | 0.5261  39 | 0.97689  5 |  |  | 1.252553 | 5.0 | 0.375 | 0.771937 |

| **PNN** | Q9H307 | 4.31624  7 | 1.636  716 | 0.967262 | 0.8133  85 | 1.71690  7 | 0.375 | 0.524  185 | 1.080728 | 5.0 |  | 4.257579 |
| --- | --- | --- | --- | --- | --- | --- | --- | --- | --- | --- | --- | --- |
| **CAV1** | Q03135 | 2.85009 | 3.464  3 | 4.53106 | 5.0 | 2.92325  3 | 4.657775 | 2.570  017 | 2.77728 | 2.885  229 | 2.04713  2 | 5.0 |
| **ELAVL1** | Q15717 | 2.56123  3 | 5.0 | 3.235587 | 1.7611  71 | 2.41150  5 | 1.517139 | 1.786  25 | 2.303596 | 5.0 | 1.22646  6 | 2.311492 |
| **NAP1L1** | P55209 | 3.04547  8 | 2.203  797 | 0.715197 |  | 1.02322 |  |  | 1.690292 | 4.673  307 |  | 0.792066 |
| **SDC4** | P31431 | 2.36213  5 | 1.644  32 | 2.22782 | 1.7362  01 | 5.0 | 4.49363 | 4.299  841 | 1.324129 | 1.995  224 | 0.1875 | 5.0 |
| **NCKAP1** | Q9Y2A7 | 3.73241 | 4.525  228 | 1.099747 | 0.9209  58 | 4.21186  7 | 0.91461 | 0.645  845 | 1.256332 | 1.805  886 | 0.84375 | 3.601107 |
| **ABCF2** | Q9UG63 | 1.21718  3 | 3.560  948 | 1.029436 | 0.7144  82 | 0.85182 |  |  | 2.389198 | 3.377  869 | 1.11762  2 | 1.09318 |
| **CCAR2** | Q9HD12 | 1.46312  3 | 2.084  584 | 0.925323 |  | 1.14148  8 |  | 0.813  458 | 5.0 | 5.0 | 1.05999  1 | 2.411646 |
| **NNMT** | P40261 | 1.52667  2 | 4.415  256 | 1.130303 |  | 1.99427  8 |  | 0.827  505 | 1.879627 | 2.528  309 | 0.78623  5 | 1.242415 |
| **OAT** | P04181 | 1.60950  9 | 1.853  993 | 1.477006 | 0.8184  3 | 1.75583  5 | 1.122808 | 1.430  408 | 5.0 | 4.367  11 | 1.48257  3 | 1.767764 |
| **ERAP1** | Q9NZ08 | 1.22965 | 5.0 | 5.0 | 1.6541  45 | 5.0 | 1.328327 | 1.736  277 | 1.239349 | 2.704  338 | 0.14062  5 | 3.103189 |
| **DHCR7** | Q9UBM7 | 1.55737  6 | 4.538  397 | 5.0 | 1.2370  58 | 2.13579  7 | 1.147273 | 1.492  877 | 1.833724 | 5.0 | 1.39239  4 | 3.438282 |
| **ATAD3A** | Q9NVI7 | 1.12869  9 | 2.681  936 | 1.451419 |  | 1.04526  3 | 1.15581 | 0.835  729 | 5.0 | 2.174  723 |  | 0.740599 |
| **TCOF1** | Q13428 | 3.12663 | 4.800  053 | 2.535213 | 2.5157 | 2.98616  4 | 2.359342 | 2.570  454 | 2.837355 | 4.931  148 | 1.72065  6 | 3.029914 |
| **DST** | Q96J76 | 5.0 | 4.516  979 | 4.108317 | 1.0902  61 | 4.61740  6 | 1.360471 | 1.067  398 | 2.114147 | 4.419  007 | 0.375 | 5.0 |
| **MGST1** | P10620 | 1.25839  5 | 2.985  868 | 4.519168 | 0.7688  16 | 2.08008  1 | 0.860523 | 4.242  234 | 5.0 | 3.346  214 | 4.30179  1 | 4.282346 |

| **RELA** | Q04206 | 2.43560  2 | 5.0 | 1.973861 | 1.8534  71 | 2.62893  8 | 1.45937 | 1.944  161 | 2.399557 | 5.0 | 1.63378  9 | 2.528169 |
| --- | --- | --- | --- | --- | --- | --- | --- | --- | --- | --- | --- | --- |
| **KRT72** | Q14CN4 | 2.98984  4 | 4.608  156 |  |  | 4.33714  1 |  |  | 2.717936 | 1.604  074 | 1.24999  7 | 1.350604 |
| **NAA15** | Q9BXJ9 | 1.49272  8 | 4.754  334 | 1.19017 |  | 1.15462  3 | 2.077561 |  | 1.116556 | 5.0 |  | 1.845645 |
| **SRSF1** | Q07955 | 2.12473  2 | 3.002  174 | 1.741958 | 1.2825  56 | 2.05066 | 1.234296 | 1.319  23 | 2.022234 | 5.0 | 0.81255 | 1.929156 |
| **CDK1** | P06493 | 5.0 | 4.814  872 | 4.428399 | 1.7443  89 | 4.50042  8 | 1.966536 | 1.908  507 | 5.0 | 4.745  79 | 2.29002  1 | 2.433193 |
| **PABPC4** | Q13310 | 1.09974  3 | 4.939  453 | 0.867882 |  | 1.22957  5 |  |  | 1.256512 | 5.0 |  | 0.90782 |
| **SF3B2** | Q13435 | 1.10136  6 | 2.144  516 | 0.572696 |  | 0.82828  1 |  |  | 0.850208 | 5.0 |  | 0.73472 |
| **SEC63** | Q9UGP8 | 1.36720  1 | 4.411  98 | 5.0 | 1.2519  98 | 1.28662  4 | 1.72931 | 1.324  825 | 1.696469 | 2.591  191 | 1.43444  5 | 2.576868 |
| **MVP** | Q14764 | 5.0 | 4.803  16 | 1.28684 | 1.5426  08 | 4.33813  4 | 0.976823 | 1.475  329 | 1.375899 | 5.0 | 0.57631  6 | 1.664441 |
| **COPG2** | Q9UBF2 | 0.72122  9 | 4.563  134 | 4.177956 | 0.8893  81 | 0.375 | 5.0 | 0.738  464 | 0.630033 | 2.272  622 |  | 0.553535 |
| **TBC1D31** | Q96DN5 | 5.0 | 1.718  747 |  |  |  | 0.605461 |  |  | 2.185  006 |  | 0.734375 |
| **DPP9** | Q8N3F5 | 4.20206  9 | 4.683  351 | 0.9889 | 0.5937  42 | 1.53729  7 |  | 0.899  028 | 1.066207 | 4.092  134 | 0.18699  9 | 1.505953 |
| **PRDX5** | Q9UKX4 | 1.68551  8 | 5.0 | 1.735163 | 0.7526  03 | 4.36817  3 | 1.15135 | 1.356  648 | 5.0 | 5.0 | 5.0 | 1.670643 |
| **BSG** | P35613 | 2.26381  5 | 2.184  941 | 2.772811 | 2.2712  58 | 4.58169  5 | 4.440234 | 2.687  275 | 4.40237 | 2.300  432 | 1.38464  3 | 5.0 |
| **RCC2** | Q9P258 | 5.0 | 4.492  076 | 0.779514 | 2.0 | 1.15456  6 |  | 0.512  101 | 0.868915 | 4.629  969 |  | 3.57092 |
| **RPS13** | P62277 | 1.89680  6 | 5.0 | 1.407683 | 1.2349  37 | 4.40882  6 | 1.703846 | 1.261  957 | 1.98123 | 5.0 |  | 1.859702 |

| **ACSL4** | O60488 | 1.62462  5 | 2.014  697 | 5.0 | 1.4103  17 | 4.43078  6 | 2.942257 | 1.547  858 | 4.292341 | 1.774  466 | 3.93727  4 | 5.0 |
| --- | --- | --- | --- | --- | --- | --- | --- | --- | --- | --- | --- | --- |
| **EIF5A** | P63241 | 1.70173  4 | 4.708  5 | 5.0 |  | 1.82946  8 | 0.598595 | 0.764  479 | 2.136376 | 5.0 | 0.63037  8 | 1.347981 |
| **RAC1** | P63000 | 4.17325  4 | 4.710  915 | 4.439816 | 5.0 | 4.52947  4 | 5.0 | 1.804  751 | 2.334609 | 3.047  874 | 1.41166 | 5.0 |
| **SPRR2E** | P22531 | 0.60180  6 | 4.243  75 |  |  | 2.17727  1 |  |  |  |  |  | 4.199855 |
| **DDX1** | Q92499 | 1.37962  5 | 4.748  264 | 1.349211 | 1.4622  1 | 1.29419  7 | 0.781328 | 0.919  376 | 4.289341 | 5.0 |  | 1.208428 |
| **ASNS** | Q549T9 | 1.53363  6 | 4.791  781 | 2.080928 | 0.9709  25 | 1.89562  4 | 1.393441 | 1.398  851 | 2.298289 | 2.657  754 | 1.14201  9 | 1.615399 |
| **UPP1** | Q16831 | 1.18915  9 | 4.658  458 | 1.04121 |  | 1.35886  6 | 0.1875 | 0.750  856 | 2.017436 | 4.544  756 | 1.36486  9 | 1.395195 |
| **PPP2R1A** | P30153 | 5.0 | 5.0 | 1.309014 | 0.6376  71 | 4.30201  5 | 0.960281 | 0.893  773 | 5.0 | 5.0 |  | 3.675131 |
| **DDX42** | Q86XP3 | 0.63436  7 | 4.466  315 | 0.577965 |  |  |  |  | 0.920959 | 5.0 |  |  |
| **CERS2** | Q96G23 | 1.26283  1 | 1.561  755 | 5.0 | 1.3993  96 | 1.67692  6 | 2.361457 | 1.903  781 | 1.90393 | 2.606  466 | 0.95909  3 | 3.607504 |
| **ZC3HAV1** | Q7Z2W4 | 1.36575 | 4.690  85 | 1.407057 | 1.2895  94 | 1.97290  6 | 2.440206 | 1.289  425 | 1.634765 | 4.015  271 |  | 1.453396 |
| **SMC3** | Q9UQE7 | 5.0 | 4.479  77 | 1.005914 |  | 1.41141  5 | 0.729851 | 0.548  879 | 1.549969 | 5.0 | 0.60776  2 | 1.37451 |
| **AP2B1** | P63010 | 1.75012  7 | 4.655  349 | 1.201476 | 4.3932  52 | 1.58500  8 | 1.759333 | 4.330  823 | 1.284573 | 1.787  572 | 0.68078  8 | 5.0 |
| **KRT27** | Q7Z3Y8 | 3.41234  4 | 4.498  33 |  |  | 4.12323  6 |  |  |  | 1.453  125 |  |  |
| **ERP29** | P30040 | 2.67922  5 | 1.683  302 | 5.0 | 1.2277  53 | 3.21239  6 | 1.779312 | 1.124  546 | 1.716162 | 2.554  33 | 0.69183  2 | 1.609102 |
| **RHOC** | P08134 | 2.92142  7 | 4.737  488 | 1.889998 | 2.0858  45 | 4.5203 | 1.519035 | 1.717  72 | 2.195682 | 2.891  735 | 1.05737  1 | 5.0 |

| **HNRNPUL1** | Q9BUJ2 | 1.01129  2 | 1.676  813 |  |  | 0.62427  1 |  |  | 0.637979 | 5.0 | 0.375 | 0.955569 |
| --- | --- | --- | --- | --- | --- | --- | --- | --- | --- | --- | --- | --- |
| **PRPF8** | Q6P2Q9 | 1.41150  1 | 2.476  671 | 0.805004 | 0.8912  87 | 1.25469  2 | 1.122976 |  | 1.291151 | 5.0 | 0.20138  7 | 0.919907 |
| **ANXA6** | P08133 | 2.43219  2 | 3.784  687 | 2.015201 | 5.0 | 4.50134  1 | 1.814967 | 5.0 | 3.170997 | 2.912  386 | 1.09712  8 | 2.649252 |
| **SUPT16H** | Q9Y5B9 | 1.20781  6 | 2.343  369 | 0.588828 |  | 0.95357  8 |  |  | 1.03457 | 4.832  718 |  | 0.562742 |
| **STT3A** | P46977 | 1.55857  2 | 1.501  018 | 5.0 | 0.9297  85 | 1.59019 | 1.525915 | 1.268  287 | 1.509103 | 1.781  503 |  | 3.731058 |
| **FKBP5** | Q13451 | 2.31300  6 | 4.729  239 | 1.783951 | 1.2174  03 | 4.42082  9 | 1.165325 | 1.372  89 | 1.9579 | 4.830  214 | 0.83877  2 | 2.052308 |
| **HMGB1** | P09429 | 2.32257  3 | 2.870  807 | 2.368807 | 3.8893  3 | 5.0 | 1.427624 | 2.419  643 | 2.670074 | 5.0 | 1.50392  7 | 4.539293 |
| **SF1** | Q969H7 | 1.18743  9 | 0.834  054 | 0.616764 |  | 1.09562  8 |  |  | 0.871142 | 5.0 |  | 0.79228 |
| **MICAL3** | Q7RTP6 | 3.66532 | 4.619  757 | 0.801246 | 1.1625  62 | 4.18069  5 | 1.364313 | 0.664  052 | 0.682924 | 5.0 |  | 4.251585 |
| **KIF23** | Q02241 | 5.0 | 4.604  343 | 1.15967 | 1.7455  55 | 2.99053  7 | 1.427743 | 1.141  357 | 1.315463 | 5.0 | 0.77088  8 | 1.953375 |
| **PTPLAD1** | Q9P035 | 1.18607  5 | 2.408  292 | 5.0 | 0.8718  57 | 1.26758  6 |  | 0.899  425 | 1.762489 | 4.428  7 | 0.5625 | 2.314662 |
| **PYCR1** | P32322 | 1.74192  6 | 3.241  24 | 1.690052 | 0.8851  91 | 2.20283  6 | 1.23327 | 1.037  823 | 5.0 | 2.035  65 | 1.32247  2 | 1.851711 |
| **EHD4** | Q9H223 | 1.70913  2 | 3.752  655 | 4.272192 | 5.0 | 4.43143 | 1.423384 | 1.419  906 | 1.551607 | 4.554  253 | 0.50354  9 | 3.739561 |
| **UNC45A** | Q9H3U1 | 1.68964  1 | 4.710  44 |  |  | 0.70405  8 | 4.291818 |  | 1.381496 | 4.587  589 | 0.375 | 1.191967 |
| **GOT1** | P17174 | 1.41310  2 | 4.587  469 | 0.969941 |  | 4.30594  8 | 0.777852 | 2.485  773 | 2.258153 | 4.477  789 | 1.80906  2 | 1.217366 |
| **FUBP1** | Q96AE4 | 1.52065  3 | 2.276  711 | 0.971797 | 0.6109  37 | 1.635 |  | 0.701  43 | 1.255359 | 5.0 |  | 1.042243 |

| **BZW2** | Q9Y6E2 | 2.06971  5 | 2.299  02 |  |  |  |  |  | 1.467435 | 1.785  561 | 0.51562  5 | 1.713636 |
| --- | --- | --- | --- | --- | --- | --- | --- | --- | --- | --- | --- | --- |
| **HSPH1** | Q92598 | 2.97140  7 | 5.0 | 1.85304 | 1.2514  03 | 4.29116  6 | 0.769822 | 1.095  43 | 1.873957 | 4.659  301 | 0.375 | 1.35798 |
| **UGP2** | Q16851 | 1.40209  8 | 4.699  408 | 1.705762 | 0.8787  17 | 4.38367  3 | 1.718708 | 0.774  966 | 1.888178 | 4.324  674 | 1.30062 | 1.789738 |
| **MKL1** | Q96SC6 | 2.50434 | 4.644  844 | 1.387911 | 1.0066  53 | 2.44865  2 | 1.159088 | 1.129  809 | 1.978415 | 5.0 | 0.84536  7 | 2.337131 |
| **FLNC** | Q14315 | 4.00306  7 | 4.728  898 | 1.600815 | 1.1905  25 | 2.22443  7 | 1.127049 | 1.493  978 | 2.152303 | 2.140  404 | 1.09980  9 | 4.698098 |
| **EIF3I** | Q13347 | 1.33280  6 | 4.766  212 | 0.976318 | 0.5902  07 | 4.24104  9 | 0.758257 |  | 1.108774 | 3.529  676 |  | 0.948364 |
| **TRIP13** | Q15645 | 3.69485 | 3.211  205 | 0.697585 |  | 1.06406  3 |  | 0.541  279 | 1.288576 | 3.834  46 |  | 0.849874 |
| **STIM1** | Q13586 | 3.91850  1 | 2.685  29 | 5.0 | 1.8167  42 | 2.50993  2 | 1.664284 | 1.899  93 | 2.325923 | 2.248  813 | 1.62643  5 | 5.0 |
| **RRAS2** | P62070 | 1.63646  4 | 3.833  967 | 4.224354 | 1.0918  65 | 4.29823  8 | 1.08778 | 0.872  945 | 1.59076 | 3.193  324 | 0.60121  4 | 3.723096 |
| **PGAM5** | Q96HS1 | 1.37621  9 | 2.072  171 | 1.538554 | 1.1377  31 | 1.35603  1 |  | 1.699  463 | 4.49346 | 1.708  785 | 1.11881  5 | 1.74269 |
| **MTCL1** | Q9Y4B5 | 5.0 | 1.375  003 |  |  | 2.39677  8 | 1.806957 |  |  | 3.458  012 |  | 4.0 |
| **CLPP** | Q16740 | 1.44722  4 | 2.077  835 | 1.833376 | 1.0111  05 | 1.75169  5 | 1.385056 | 1.352  396 | 5.0 | 2.236  453 | 1.22268  9 | 1.67619 |
| **TNC** | P24821 | 1.70904 | 2.350  875 | 4.231693 | 1.1925  07 | 4.72483  1 | 0.91039 | 1.156  743 | 1.168892 | 2.760  955 |  | 3.300823 |
| **HMGB3** | O15347 | 2.43085  9 | 1.396  41 | 0.703344 | 0.9172  85 | 1.69974  6 |  | 0.850  182 | 1.102388 | 4.699  977 |  | 1.337244 |
| **GNAS** | Q5JWF2 | 1.85625  5 | 5.0 | 1.485331 | 1.3059  9 | 4.42014  2 | 5.0 | 1.162  581 | 1.759657 | 5.0 | 1.41439  7 | 5.0 |
| **SNX1** | Q13596 | 1.85098  2 | 4.136  862 | 1.58483 | 5.0 | 1.38699  4 | 4.209674 | 4.489  093 | 1.19375 | 2.145  412 | 1.02656 | 2.111352 |
| **PSMC4** | P43686 | 1.53426  8 | 4.790  602 | 1.584113 | 0.7054  63 | 1.30632  2 | 0.676518 | 1.502  689 | 1.552321 | 5.0 | 1.41371  2 | 1.02438 |
| **WDHD1** | O75717 | 1.61820  3 | 2.527  361 | 0.895906 | 0.6947  48 | 1.53489  2 | 0.518121 | 0.741  379 | 1.41602 | 4.852  366 | 0.90147  4 | 1.376267 |
| **TAF1** | Q70Q87 | 1.90975  4 | 2.811  052 | 1.413375 | 1.2200  15 | 2.07800  7 | 0.97901 | 1.256  552 | 1.776316 | 5.0 | 0.81962  8 | 1.915929 |
